# Synthesis and In Vitro Studies of Photoactivatable Semisquaraine-type Pt(II) Complexes

**DOI:** 10.1021/acs.inorgchem.1c03957

**Published:** 2022-05-06

**Authors:** Kevin Morales, Sergi Rodríguez-Calado, Jordi Hernando, Julia Lorenzo, Antonio Rodríguez-Diéguez, Carlos Jaime, Pau Nolis, Mercè Capdevila, Òscar Palacios, Marta Figueredo, Pau Bayón

**Affiliations:** † Departament de Química, Universitat Autònoma de Barcelona, 08193 Cerdanyola del Vallès, Spain; ‡ Institut de Biotecnologia i Biomedicina (IBB) and Departament de Bioquímica i Biologia Molecular, Campus UAB, 08193 Cerdanyola del Vallès, Spain; § Department of Inorganic Chemistry, Faculty of Science, University of Granada, Av/Severo Ochoa s/n, 18071 Granada, Spain

## Abstract

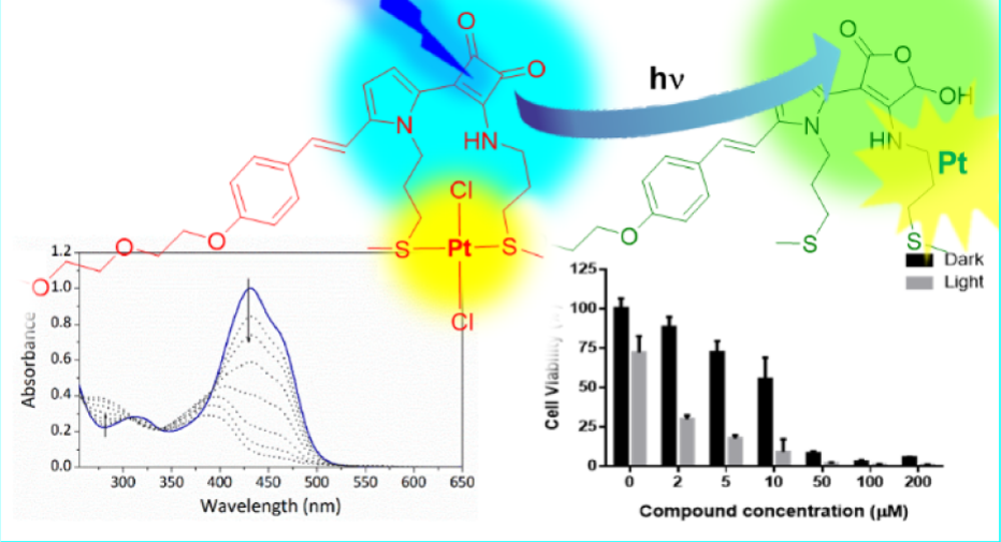

The synthesis, full characterization, photochemical properties, and cytotoxic activity toward cisplatin-resistant cancer cell lines of new semisquaraine-type Pt(II) complexes are presented. The synthesis of eight semisquaraine-type ligands has been carried out by means of an innovative, straightforward methodology. A thorough structural NMR and X-ray diffraction analysis of the new ligands and complexes has been done. Density functional theory calculations have allowed to assign the *trans* configuration of the platinum center. Through the structural modification of the ligands, it has been possible to synthesize some complexes, which have turned out to be photoactive at wavelengths that allow their activation in cell cultures and, importantly, two of them show remarkable solubility in biological media. Photodegradation processes have been studied in depth, including the structural identification of photoproducts, thus justifying the changes observed after irradiation. From biological assessment, complexes **C7** and **C8** have been demonstrated to behave as promising photoactivatable compounds in the assayed cancer cell lines. Upon photoactivation, both complexes are capable of inducing a higher cytotoxic effect on the tested cells compared with nonphotoactivated compounds. Among the observed results, it is remarkable to note that **C7** showed a PI > 50 in HeLa cells, and **C8** showed a PI > 40 in A2780 cells, being also effective over cisplatin-resistant A2780cis cells (PI = 7 and PI = 4, respectively). The mechanism of action of these complexes has been studied, revealing that these photoactivated platinum complexes would actually present a combined mode of action, a therapeutically potential advantage.

## Abstract 2

The synthesis, full characterization, photochemical properties, and cytotoxic activity toward cisplatin-resistant cancer cell lines of new semisquaraine-type Pt(II) complexes are presented. Eight semisquaraine-type ligands and their corresponding Pt(II) complexes have been studied. These complexes have turned out to be photoactive at wavelengths that allow their activation in cell cultures. Two of them display remarkable solubility in biological media showing a promising behavior as photoactivatable compounds against several cancer cell lines.

## Introduction

For decades, platinum complexes have focused a great interest because of their particular features.^1^ In recent years, these complexes have re-emerged as very motivating entities for different purposes.^2^ In particular, photoresponsive complexes have become significant,^3^ being one of the fields in cancer therapies where most efforts have been invested.^4^ In the last years, platinum(II) phototherapy has been shown as an opportunity to lessen the side effects associated to cisplatin-type drugs.^5^ Roughly, phototherapy can be classified into two categories: photodynamic therapy (PDT) and photoactivated chemotherapy (PACT). Both PDT and PACT are proving to be good options as new cancer therapies.^6^ While PDT needs the presence of molecular oxygen to elicit cell death, PACT acts by means of the species generated by a prodrug as its photoresponse.^7^ In this way, PACT allows controlling where and when the active species are generated, selectivity is thus increased, and therefore, the necessary dose is reduced. Such an approach allows minimizing side effects, hence improving the quality of life of the treated patients.

**Figure 1:**
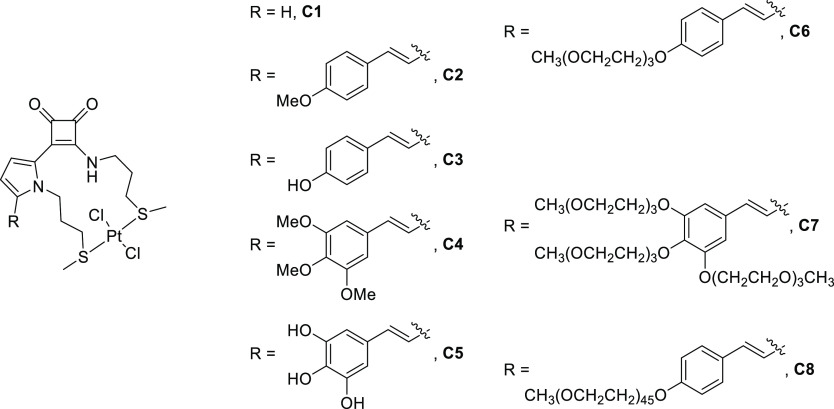
New semisquaraine-type Pt(II) complexes in this study.

However, despite all the efforts being made, there are some aspects that remain unsolved associated with the physical properties of the platinum complexes themselves. One of these limitations is, for example, the stability of the complexes in the physiological medium and the dark. This refers not only to the photostability of the complex but also to the resistance to hydrolysis.^8^ The solubility of platinum(II) species in aqueous media is another concern. In general, neutral complexes have low solubility; however, in order to make them work, they must have a certain solubility in physiological media,^9^ though keeping an equilibrium with lipophilicity, as this feature is believed to be crucial for the passive influx of drug molecules into cells.^10^ Also, the frequency of irradiation where a complex response occurs is critical, as ideally, it should take place in the frequency range known as the therapeutic window.^5e^ In this article, we want to present a new class of platinum(II) complexes and how we overcome the limitations pointed out above. Thus, herein we report the synthesis of new ligands and their corresponding platinum(II) complexes and their complete photochemical characterization. Recently, we have described the synthesis and photochemical assessment and activity against HeLa cells of new squaramide-based platinum(II) complexes.^11^ Although promising results were obtained, some limitations were noticeable. Those stable complexes showed moderate to low solubility in aqueous media and, importantly, all of them were photoresponsive in the UV range, clearly out of the therapeutic window. In the study herein reported, the aforementioned problems have been alleviated. We believe that these new complexes may have applications given their ability of photoresponse. The new ligands here presented are semisquaraine-type (Figure 1), and based on the conclusions of our previous study, they bear a thioalkylamino branch as a cooperative cyclobutenedione substituent, considering its chelation abilities. Also, unlike our former squaramide-type ligands, the other substituent of the ring has been used to increase electronic delocalization and, therefore, modify the absorption range of these new complexes. Finally, several structural modifications for the improvement of the solubility of the complexes are discussed.

## Results and Discussion

### Synthesis

In view of our previous findings, we planned to synthesize a new series of cyclobutenedione-based platinum(II) complexes fulfilling the following requirements: (i) bearing two 3-thiopropylamino appendixes, since this kind of chelators proved to be efficient for the Pt(II) release from the complex; (ii) enhanced water solubility, to improve the availability in a physiological medium; and (iii) photochemical response in a biocompatible frequency range. We visualized that the attachment of a pyrrole linker to the cyclobutenedione core could hopefully facilitate the accomplishment of these requirements since such a subunit should be amenable to subtle structural modifications, oriented to achieve the desired properties in the new photoactive complexes. Thus, an accessory phenyl residue conjugated to the heterocycle would extend the π delocalization, providing a chromophore suitable for a low-frequency photoactivation, while the addition of hydroxyl groups and their PEG derivatives would improve water solubility. Moreover, pyrrole is considered a privileged scaffold for biomedical applications.^12^ Accordingly, the synthesis of complexes **C1–8** (Figure 1) was undertaken.

**Scheme 1:**
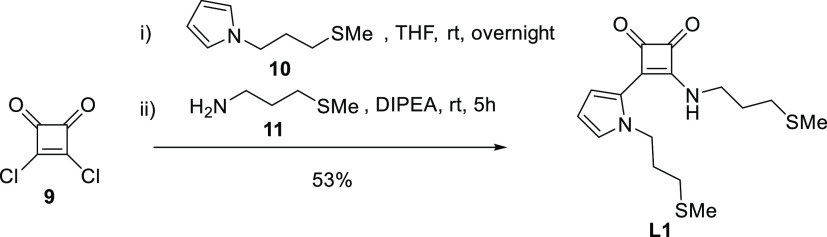
Sequential Strategy in the Synthesis of Ligand **L1**

The first endeavor was the preparation of the corresponding ligands, **L1** to **L8**. To this aim, we developed a one-pot protocol consisting of the consecutive addition of the pyrrole and the methylthiopropylamino nucleophilic moieties to the cyclobutenedione core. The proper conditions were set up by investigating, in depth, the reaction depicted in Scheme 1. The pyrrole component **10** was prepared by N-alkylation of pyrrole according to a known procedure.^13^ Successive conjugate addition of **10** (in a molar ratio **9**/**10** = 1/1) and then a mixture of thioamine **11** and diisopropylethylamine (DIPEA) in a molar ratio **9**/**11**/DIPEA = 1/1/2, to a solution of dichlorocyclobutenedione **9**(14) in tetrahydrofuran (THF), furnished the expected ligand **L1** in a single operation and a 53% isolated yield after crystallization from MeOH.

**Scheme 2:**
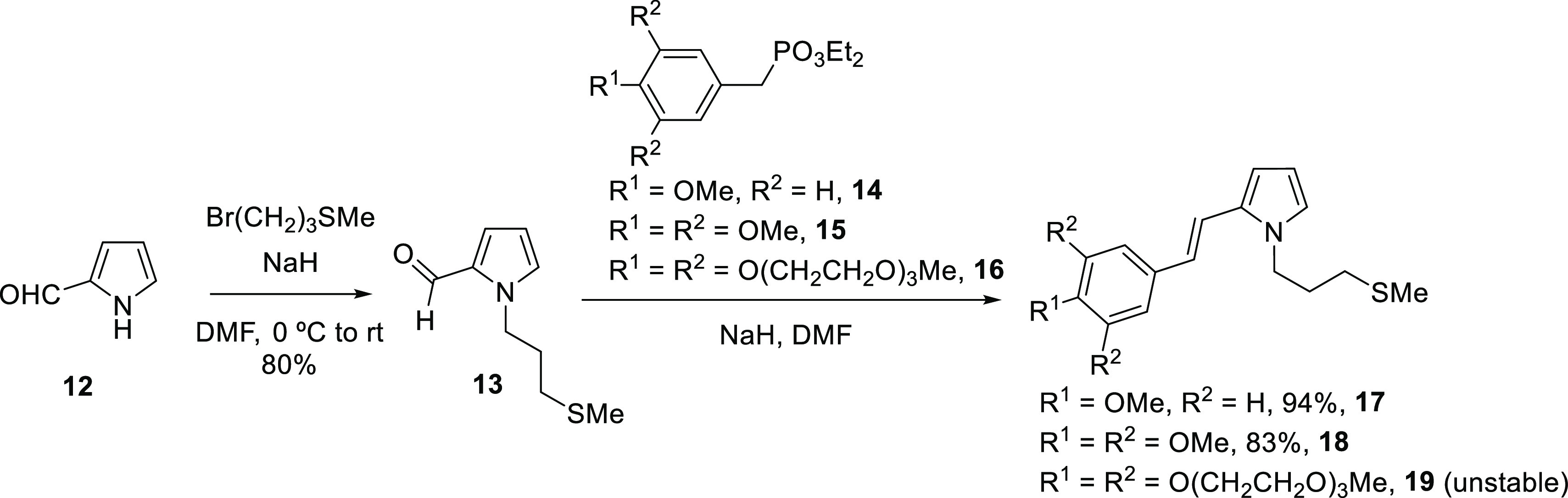
Synthesis of Substituted Pyrrole Motives

Next, we prepared the required pyrrole derivatives for the synthesis of the other targeted ligands (Scheme 2). N-alkylation of 1*H*-pyrrole-2-carbaldehyde, **12**, with (3-bromopropyl)(methyl)sulfane furnished aldehyde **13**, which was subjected to a Horner–Wadsworth–Emmons (H–W–E) alkenylation with the benzylic phosphonates **14**(15) and **15**,^16^ affording the corresponding pyrrole derivatives **17** and **18**, respectively, in good yields. It is worth mentioning that alkene **17** showed a limited stability, even at freezing temperatures, leading to degradation products after several weeks. Hence, it should be soon subjected to the next synthetic step. On the contrary, its congener **18** showed an unlimited stability.

**Figure 2:**
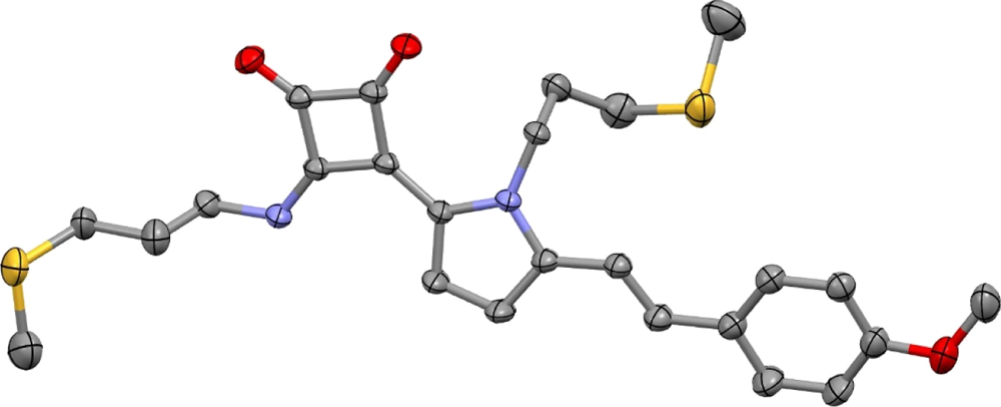
MERCURY drawing for **L2**. Thermal ellipsoids are drawn at the 30% probability level. H atoms are omitted for clarity.

**Scheme 3:**
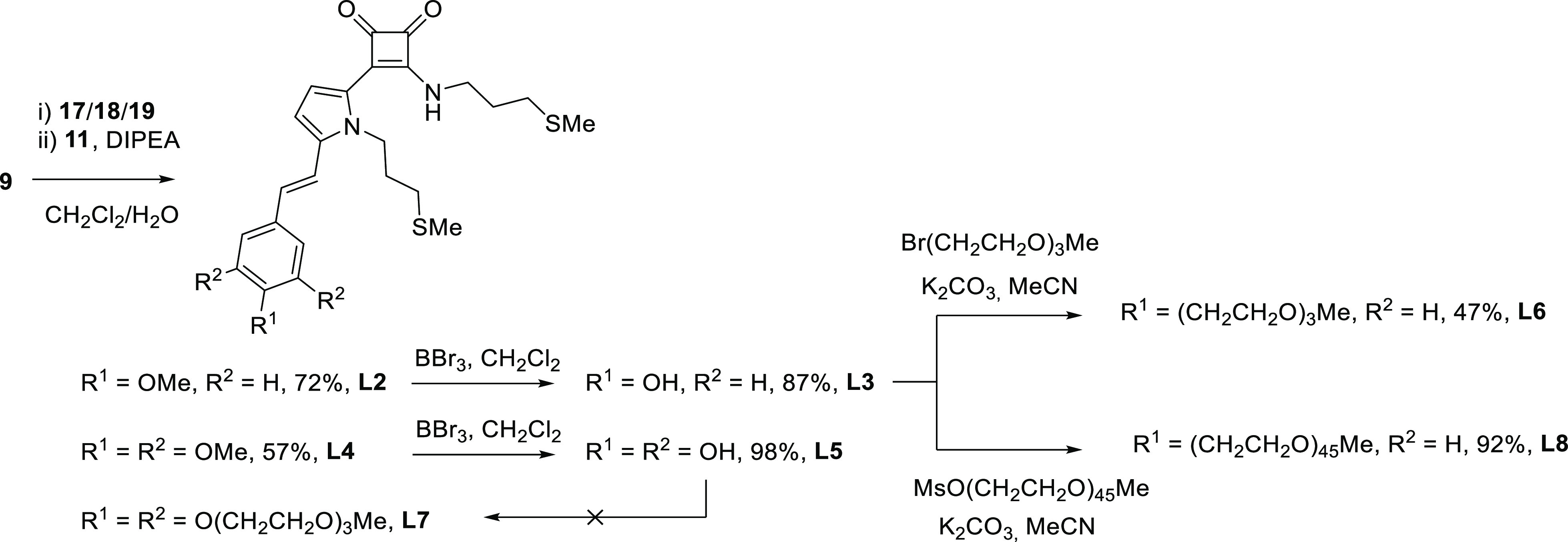
Synthesis of Conjugate Pyrrole Ligands **L2–8**

When the conditions developed for the preparation of **L1** from the simpler pyrrole **10** were applied to **17**, after chromatographic purification followed by crystallization from CH_2_Cl_2_/Et_2_O, the expected ligand **L2** was obtained as a yellow powder in a 60% yield (Scheme 3). Moreover, it was found that the same reaction performed in a two-phase CH_2_Cl_2_/H_2_O system allowed the isolation of pure **L2** in a 72% yield by straight crystallization after the addition of Et_2_O to the dried organic phase, without the need of a previous chromatographic separation. This improved protocol applied to pyrrole **18** furnished the corresponding ligand **L4** as an orange powder in a 57% yield. Eventually, crystallization of **L2** from dimethylformamide (DMF)/H_2_O provided crystal needles suitable for X-ray analysis (Figure 2), which confirmed the 1,2-disubstitution of the cyclobutenedione core, the *E* configuration of the alkene, and a preferential fully extended conformation of the two substituents, with the NH *syn* to the pyrrole moiety.

Treatment of **L2** and **L4** with tribromoborane in CH_2_Cl_2_ unveiled the hydroxyl groups, furnishing the corresponding phenols **L3** and **L5** in good yields. Phenol **L3** was readily converted into the glycol ether derivatives **L6** and **L8** in 47 and 92% yield after crystallization from methanol and CH_2_Cl_2_/Et_2_O, respectively. However, when the same methodology was intended for the synthesis of **L7**, the O-alkylation of the pyrogallol derivative **L5** failed. Alternatively, **L7** was synthesized from pyrrole **19**, prepared through an H–W–E reaction between aldehyde **13** and phosphonate **16**(17) (Scheme 2). As **19** was revealed to be very unstable, for the synthesis of **L7**, freshly prepared crude **19** was added to cyclobutenedione **9**, and after a successive addition of thioamine **11**, the expected ligand was obtained in a 62% global yield from **13**. Likewise, an alternative synthesis of **L6** starting from **13** and an appropriate phosphonate was also developed (see the Supporting Information, S3 for details), but the overall sequence yield did not improve the results of the pathway shown in Scheme 3.

All the synthesized ligands (**L1–8**) and their previously unknown precursors (**13**, **17**, and **18**) were fully characterized according to their physical and spectroscopic data.

With the ligands in hands, the next challenge was synthesizing their Pt(II) complexes. To this aim, each ligand was treated with K_2_PtCl_4_ in an appropriate solvent at room temperature under an argon atmosphere. For complexes **C1–6**, the resulting precipitate was filtered and successively washed with suitable solvents. For **C7** and **C8**, the complexes were separated from the reaction mixture by the addition of brine, followed by extraction with CH_2_Cl_2_. Table 1 summarizes conditions, yield, and physical description for each particular complex.

**Table 1:** Synthesis of Complexes **C1–8**^a^

entry	solvent	time (h)	precipitate washing^b^	complex (yield)	color, mp (from solvent)
1	MeOH/H_2_O, 1:1	17	MeOH; H_2_O	**C1** (56%)	brown, 173–178 °C (MeOH/H_2_O)
2	THF/H_2_O, 3:2	22	THF; H_2_O	**C2** (97%)	orange, 184–187 °C (THF/H_2_O)
3	THF/H_2_O, 1:1	15	H_2_O; MeOH; THF	**C3** (86%)	russet, >200 °C (THF/H_2_O)
4	THF/H_2_O, 1:1	20	THF; H_2_O	**C4** (99%)	orange, >200 °C (THF/H_2_O)
5	THF/H_2_O, 1:1	16	H_2_O; MeOH; THF	**C5** (85%)	brown, >200 °C (THF/H_2_O)
6	THF/H_2_O, 1:1	16	H_2_O; MeOH; THF	**C6** (81%)	orange, 135–139 °C (THF/H_2_O)
7	THF/H_2_O, 1:1	17	c	**C7** (65%)	brown, 92–95 °C (CH_2_Cl_2_/Et_2_O)
8	H_2_O	17	c	**C8** (95%)	brown, 50–53 °C (CH_2_Cl_2_/Et_2_O)

a All the reactions were performed by the treatment of the starting ligands with 1 M equivalent of K_2_PtCl_4_ at room temperature, under an argon atmosphere, except for entry 8, where the molar ratio K_2_PtCl_4_/**L8** was 1.3.

b Complexes precipitated from the reaction mixture were filtered and successively washed with the indicated solvents.

c Complexes **C7** and **C8** were separated by extraction with CH_2_Cl_2_.

All the synthesized complexes **C1–8** were fully characterized according to their physical and spectroscopic data. HRMS or MS of all the complexes showed the characteristic pattern of platinum compounds except for **C8**, which degraded upon ionization. Since its polydisperse polymeric nature precluded elemental analysis, the amount of Pt was determined by ICP-OES (6.7% Pt), obtaining a value similar to that expected, assuming a molecular weight of 2705 Da (7.2% Pt).

The *cis*–*trans* configuration of Pt(II) complexes usually reveals crucial for the biological response to these species. Unfortunately, all attempts to obtain crystalline complexes for their structural elucidation by means of X-ray diffraction (XRD) analysis were unsuccessful. As *tert*-butyldiphenylsilyl (TBDPS) derivatives are usually prone to form crystalline solids, a TBDPS-derivative ligand **L20** and its corresponding dichloride-Pt(II) complex, **C20**, were prepared (see the Supporting Information, S5). Despite a number of attempts at crystallization, **C20** systematically turned out as a solid not suitable for XRD analysis. In view of that, we decided to investigate this issue on the basis of theoretical calculations.

### *cis*-Pt Versus *trans*-Pt Configuration Tentative Assignment

**Figure 3:**
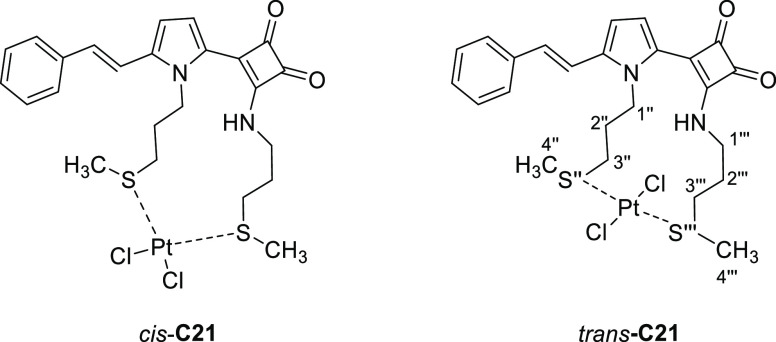
Configurations of the metal center in model complex **C21**.

Density functional theory (DFT) calculations were performed to evaluate the relative stability of the two isomers. The relative energies of both isomers were calculated for complex **C21** (Figure 3) as a model by methods based on quantum mechanics. As a starting point, the aminothiolated chains were assumed to coordinate by the sulfur atoms, based on XRD analysis for one of our previous analogs.^11^

Considering K_2_PtCl_4_ as the source of Pt(II), the *trans* effect seems to point to the *trans* configuration as the most favorable, as thioether-type ligands are rated as *trans*-director when compared to chloride ligands. With this in mind, the method of choice was the use of Gaussian program in its 2016 version (abbreviated as Gaussian 16).^18^ DFT calculations were performed with the LANL2DZ^19^ base and the M06-2X^20^ function. Structures were built with the help of graphic program GaussView,^21^ and their energies were optimized using the values defined by default in the program. Vibration frequencies were calculated for all compounds, always obtaining zero imaginary frequencies, which ensures that energy minima are considered.^22^

The complexation of sulfur atoms to the Pt(II) center causes them to become stereogenic and, therefore, may have the *R* and/or *S* configuration. The values of the obtained energies are shown in Table 2, together with the energy differences between the *cis* and *trans* isomers. The descriptors *R* and *S* refer to the absolute configuration of the sulfur atoms complexed with platinum (see the Supporting Information, Figure S1).

**Table 2:** Energetic Results of DFT Calculations with Gaussian16, M062X/LANL2DZ for *cis*- and *trans*-**C21** Considering All Possible Absolute Configurations for Sulfur Atoms^23^

entry	isomer^a^	*cis*-**C21***E* (hartree)	*trans*-**C21***E* (hartree)	Δ(*cis*–*trans*)^b^ kcal/mol	Δ(*trans*–*trans*)^b^ kcal/mol
**1**	*R*″/*S*‴	–1359.7071453	–1359.7283893	13.3	0.0
**2**	*R*″/*R*‴	–1359.7103260	–1359.7218970	11.3	4.1
**3**	*S*″/*S*‴	–1359.7082496	–1359.7247720	12.6	2.3
**4**	*S*″/*R*‴	–1359.7126554	–1359.7253195	9.9	1.9

a The configuration descriptors refer to the sulfur atom of the chain with double prime numbering (′′), and the chain with triple prime (′′′), as shown in Figure 3.

b Relative energy values referred to the most stable *trans* isomer.

The results in Table 2 indicate that structures with a *trans*-configuration at the platinum center, *trans*-**C21**, are more stable than those for *cis*-**C21** at about 10–13 kcal/mol. Thus, by extension, it was assumed that all the complexes here presented might display a *trans* configuration. Since, for these complexes, it seems that both the results of the DFT calculations and the prediction based on the *trans* effect (i.e., thermodynamics and kinetics) point in the same direction, it would be reasonable to tentatively assign these complexes a *trans* configuration.

The relative differences of the corresponding *trans*-**C21** isomers are also given. Among *trans*-**2** isomers, the most stable seems to be *R*″/*S*‴ (Table 2, entry 1), although a more complete conformational study would be needed to ensure this. The energy differences between the plausible configurations for *trans*-**C21** are less significant (less than 4.1 kcal/mol), and given the size of the Pt(II) metallocycle, very likely small conformational changes would alter the stability order.

### Photochemistry

Once the ligands and complexes were synthesized and characterized, their photochemical behavior was evaluated. First, the UV–vis spectra of both ligands and complexes were recorded (see the Supporting Information, Figure S2), whose absorption was found to be clearly bathochromically shifted relative to our previously reported squaramide-type compounds.^11^ Thus, ligand **L1** and the corresponding complex **C1** showed spectral maxima at λ_max_^abs^ ∼ 380 nm because of the introduction of a pyrrole substituent to the semisquaraine core. More interestingly, an extension of the conjugation path in the rest of the compounds by attaching an additional phenylvinyl group led to a further absorption redshift, which clearly falls in the visible range (λ_max_^abs^ ∼ 450 nm). Therefore, these results validate our strategy for the design of Pt(II) photocages that are excitable with visible light.

**Figure 4:**
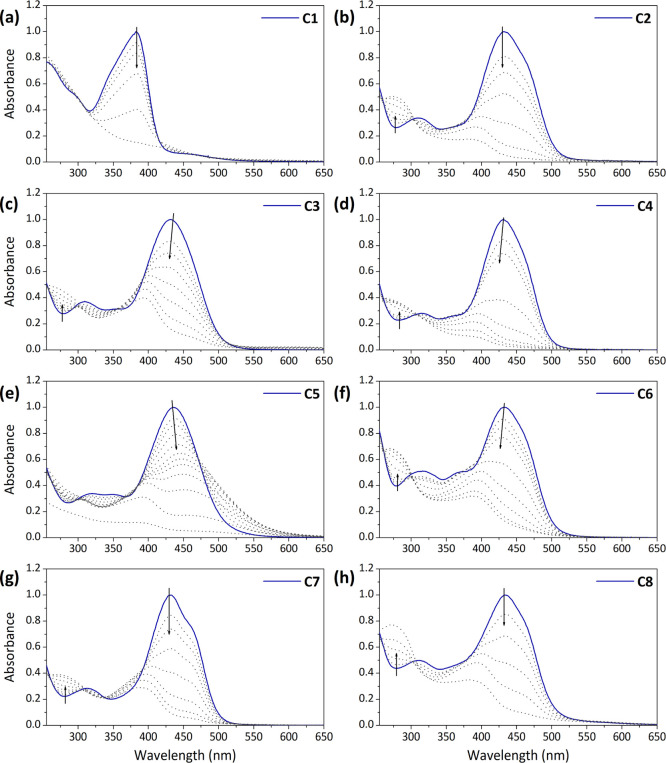
Time evolution of the UV–vis absorption spectra after irradiation of complexes (a) **C1** in water/DMSO, 98:2, (b) **C2** in water/DMF, 98:2, (c) **C3** in water/DMSO, 98:2, (d) **C4** in water/DMF, 98:2, (e) **C5** in water/DMSO, 98:2, (f) **C6** in water/DMF, 98:2, (g) **C7** in water, and (h) **C8** in water. For **C1** λ_exc_ = 365 nm (UV lamp) and for **C2–8** λ_exc_ = 450 nm (LED).

Illumination of the ligands and complexes prepared resulted in a negligible fluorescence emission regardless of the solvent used (see the Supporting Information, Table S1). Instead, concomitant irreversible changes were observed in absorption that suggest that photodegradation of these compounds occurs under irradiation. This process was investigated in detail upon photoexcitation at 365 nm for **C1** or 450 nm for **C2–8** (Figure 4). Interestingly, while for **C2–C6** the use of a cosolvent was needed, complexes **C7** and **C8** bearing PEG chains could be solubilized in pure water without the need for any additional cosolvent. For these and the rest of the compounds, very similar trends in absorption were registered upon continuous irradiation: the characteristic band at λ_max_^abs^ ∼ 380 nm or λ_max_^abs^ ∼ 450 nm rapidly faded after a few minutes, which in most of the cases resulted in the appearance of a low-intensity hypsochromically shifted signal (e.g., complexes **C2–8** in Figure 4). Interestingly, this behavior is reminiscent of that previously observed by us for squaramide-based Pt(II) complexes,^11^ which could be unambiguously ascribed to the photodegradation of the ligand.

To assess the efficiencies of the photodegradation process for all the ligands and complexes, their photoreaction quantum yields (Φ_ph_) were evaluated (see the Supporting Information, Table S2). In general, the values obtained are rather low (Φ_ph_ ∼ 10^–4^ to 10^–5^) though they are on the same order of magnitude as those reported for other visible light-active photolyzable groups.^24^ It must be noted that this effect is partially counterbalanced by the large molar extinction coefficients of our compounds (ε_max_^abs^ > 2.0 × 10^4^ M^–1^ cm^–1^, Table S2), as the efficacy of photoreactions strictly depends on the product of Φ_ph_ with the absorptivity of the irradiated molecule at the excitation wavelength. As a result, they present large enough Φ_ph_ ε_max_^abs^ values for photouncaging applications (Table S2). This is especially true for the water-soluble Pt(II) complexes developed herein, which show Φ_ph_ and Φ_ph_ ε_max_^abs^ values that are about 10-fold larger than for the corresponding free ligands and, therefore, make them especially suitable for light-induced Pt(II) release. Most probably, this is due to the restriction of conformational mobility upon metal complexation, which must decrease the efficiency of excited state relaxation through intramolecular vibrational and rotational motions.

**Scheme 4:**
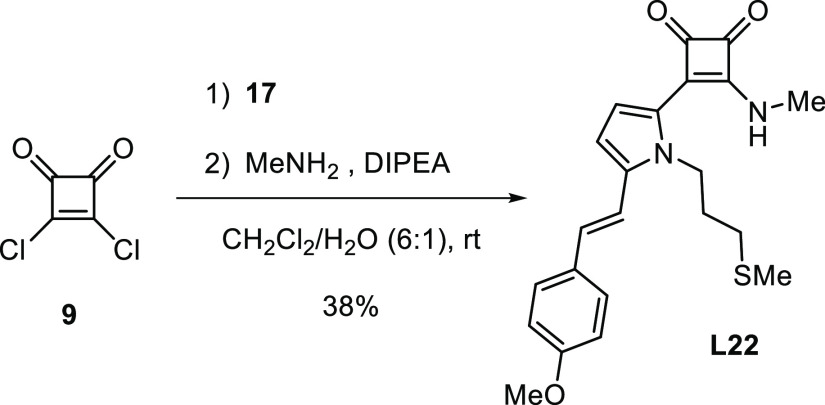
Synthesis of Simplified Ligand **L22**

At this point, knowing the nature of the photoproducts and giving a mechanistic explanation for their formation was considered as required. Initially, photodegradation of the ligands was explored. First, a 60 mM (DMF/H_2_O, 9:1) solution of ligand **L2** was irradiated (450 nm LED) until its UV–vis spectra revealed complete degradation, and the resulting mixture was analyzed by MS and NMR. Three main peaks were detected and correlated with the mass corresponding to **L2** + H_2_O {[M + H]^+^ (489.2 Da), [M + Na]^+^ (511.2 Da) and [M + K]^+^ (527.2 Da)}. Despite several purification attempts, the NMR analysis turned out to be too intricate to elucidate its structure. Therefore, as a structurally simpler model, ligand **L22** was prepared on purpose through a synthetic route similar to that of its analogs (Scheme 4).

**Scheme 5:**
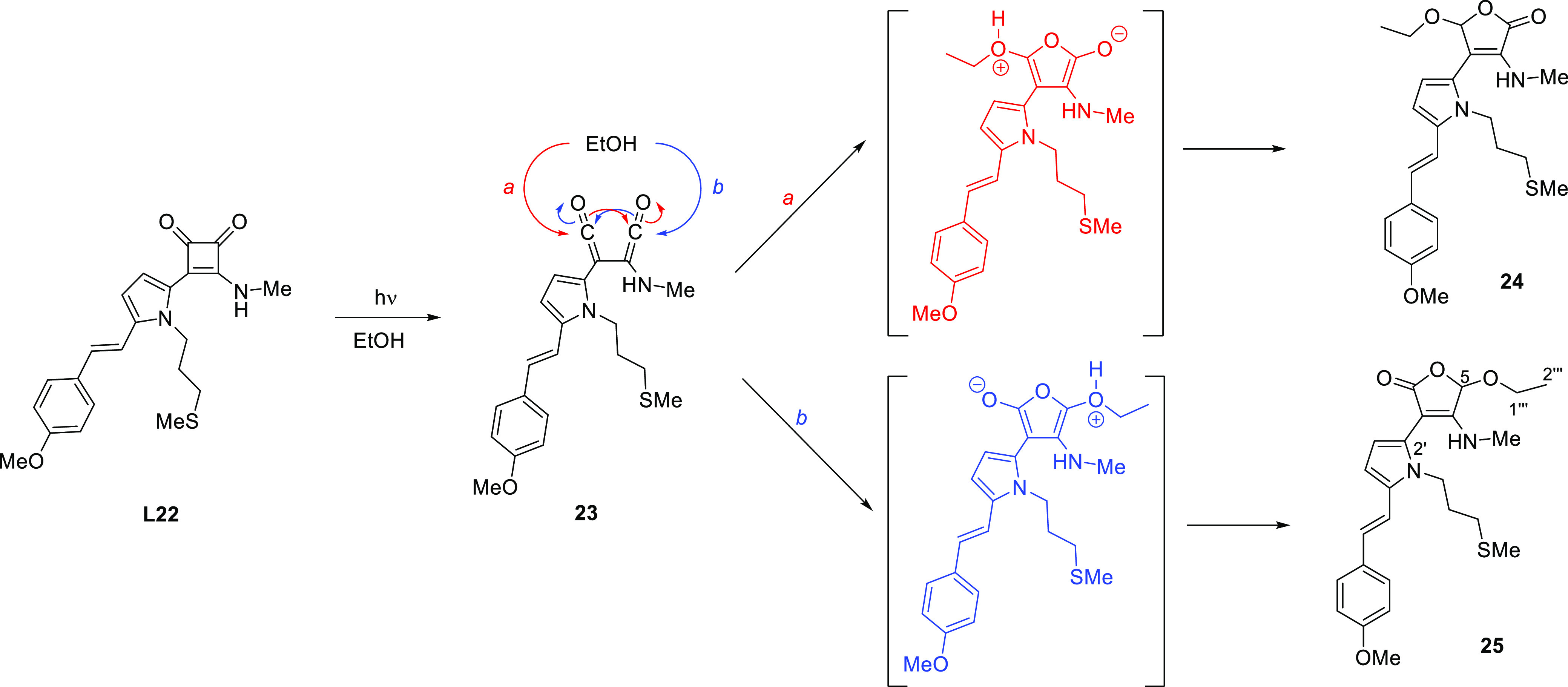
Mechanistic Proposal for the Formation of Butenolide **24** or **25** as the Major Photoproduct

We hypothesized that the photodegradation products would likely derive from bisketene intermediates, which are known to be formed upon irradiation of cyclobutenediones.^25^ With this idea in mind, the irradiation of **L22** was assayed in DMF/EtOH (6/4) instead of DMF/water, considering that the addition of the nucleophilic solvent to the ketene functionality would give us more information (particularly for NMR analysis) if an ethoxy moiety, instead of a hydroxy, was incorporated to the final degradation product. The irradiation experiment was monitored by UV spectroscopy (see the Supporting Information, Figure S3). After irradiation, a major photoproduct was detected by HRMS [443.2 Da (+H^+^), 465.2 Da (+Na^+^) and 481.2 Da (+K^+^)]. These peaks were correlated with the mass corresponding to **L22** + EtOH (see the Supporting Information, Figure S4). Also, a carbonyl group was confirmed by IR analysis (see the Supporting Information, Figure S5). After careful ^1^H NMR and ^13^C NMR analyses of a pure sample of the major degradation product (see the Supporting Information, Figures S6 and S7 respectively), one of the two regioisomeric butenolides, **24** or **25,** was assigned as the most plausible structure (Scheme 5), consisting of a mixture of 3 different conformers. This hypothesis was also consistent with a similar process previously described for bisketenes.^25,26^ Ultimately, the structure for the major photoproduct was assigned by additional NOE and HMBC experiments of the mixture of conformers. NOE Experiments were performed at 250 K (see the Supporting Information, Figure S8), to get a good resolution of the signals corresponding to each conformer at play. For one of the conformers, the spectrum showed an NOE interaction between methyl 2‴ and the NH*Me* group. For the other two conformers, NOE effects between 2‴ and the S*Me* group were also noticeable. In addition, an HMBC experiment (see the Supporting Information, Figure S9) revealed no cross signal C2′-H5. With all those pieces of evidence, the major photoproduct was assigned as butenolide **25**. The formation of **25** was envisioned as triggered by the nucleophilic attack of the nucleophilic solvent to a ketene functionality as expected (Scheme 5). A careful HRMS analysis of an irradiated sample of complex **C2** suggests that the photodegradation pathway in Scheme 5 is also effective from this Pt(II) complex. Hence, following the Pt(II) traceability, a peak [721.1 Da [**C2** + H_2_O–Cl]^+^] was detected and assigned to the corresponding butenolide Pt(II) complex (see the Supporting Information, Figure S10).

### Biological Assessment

**Figure 5:**
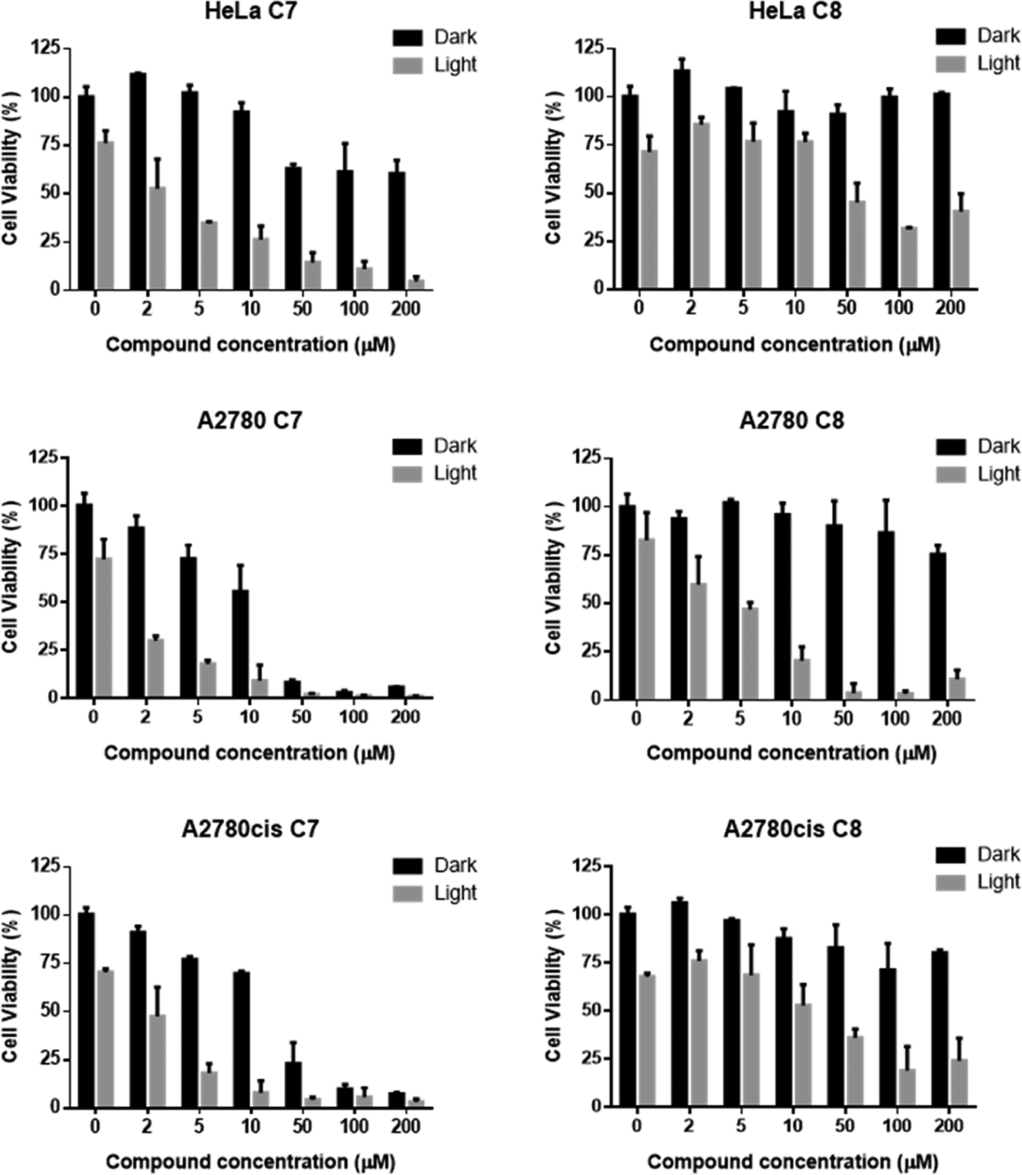
Evaluation of cytotoxicity of compounds **C7** and **C8** in the absence or presence of blue light (dark/light) in HeLa, A2780, and A2780cis human carcinoma cells after 72 h (24 h internalization) using PrestoBlue assay. Results are representative of three independent experiments with a minimum of three replicates per experiment.

Prior to initiating the evaluation of the biological activity of the synthesized complexes, a study of their solubility in an aqueous medium was performed. It was observed that, among all, complexes wearing PEG motives, **C7** and **C8**, presented excellent water solubility, as expected and, therefore, they were finally chosen as the candidates to investigate their biological properties. Accordingly, complexes **C7** and **C8** were tested against three cancer cell lines of different origins without illumination and under blue light (light dose of 32 J/cm^2^) (Table 3). Thus, HeLa, A2780, and cisplatin-resistant A2780cis cells were incubated for 24 h with stock solutions of **C7** and **C8** at different concentrations, and cell viability was evaluated at 72 h. The effect of the compound cytotoxicity was expressed as a decrease in cell viability (Figure 5),^27^ and the half inhibitory compound concentration (IC_50_) values were also calculated (Table 3).

**Table 3:** IC_50_ Determination of **C7**, **C8** Complexes and Cisplatin after 72 h (24 h Internalization) in Light and Dark in HeLa, A2780, and A2780cis Cells

			IC_50_ (μM)		
entry	complex	cell line	light^a^	dark	PI
1	**C7**	HeLa	4 ± 3	>200	>50
2	**C7**	A2780	1.4 ± 0.8	18 ± 9	13
3	**C7**	A2780cis	2.7 ± 0.7	19 ± 8	7
4	**C8**	HeLa	>50	>200	n.d.
5	**C8**	A2780	5 ± 3	>200	>40
6	**C8**	A2780cis	48 ± 22	>200	4
7	cisplatin	HeLa	15.5 ± 3.4	14.7 ± 2.6	
8	cisplatin	A2780	2.3 ± 0.3	2.8 ± 0.2	
9	cisplatin	A2780cis	15.1 ± 1.5	13.8 ± 0.2	

a λ_exc_ = 450 nm and dose ca. 32 J/cm^2^. PI = photoactive index (obtained by dividing the baseline IC_50_ value by the photoactivated IC_50_ value).

In the absence of light, **C7** displayed considerable cytotoxicity toward A2780 and A2780cis cell lines (IC_50_ of 18 ± 9 and 19 ± 8 μM, respectively) (entries 2 and 3), while in the same conditions, complex **C8** revealed no toxicity against any of the cancer cell lines. To our delight, when the complexes were illuminated, the resulting cytotoxicity increased significantly in all cases, being the most cytotoxic **C7** irradiated within A2780 cell line (IC_50_ = 1.4 ± 0.8 μM) (entry 2). Remarkably, when comparing the light effect, both complexes showed noticeable PI (photoactive index) values. In particular, **C7** in HeLa (PI > 50) and **C8** in A2780 (PI > 40) displayed remarkable results (entries 1 and 5), comparable or even improving examples for Pt(II) complexes in the PACT literature.^5c,28^

**Figure 6:**
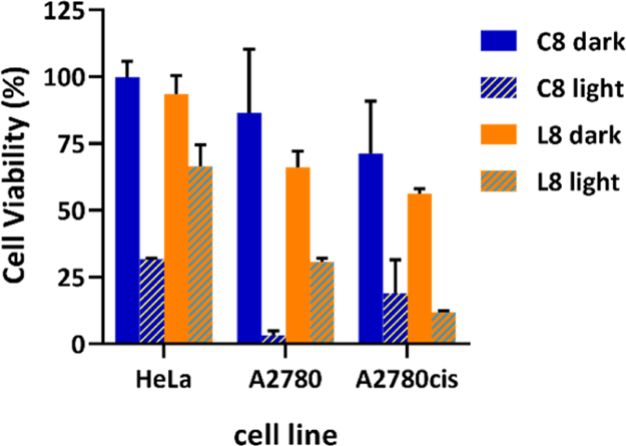
Cytotoxicity assays after 72 h incubation (24 h internalization) in the light and dark in HeLa, A2780, and A2780cis cells with complex **C8** and ligand **L8**, at 100 μM each.

Using 100 μM concentration as a reference, the photocytotoxicity of the ligands was also evaluated and compared with their corresponding Pt(II) complexes. Although for **L7**, this concentration was too high to evaluate the light effect, for **L8**, it was observed how the cytotoxicity was also enhanced upon illumination. Nevertheless, the effect is lower compared with the corresponding Pt(II) complex **C8** (Figure 6).

### Cellular Mechanism of Action of Compounds **C7** and **C8**

#### DNA Interaction

The interaction of these complexes with DNA was also investigated. To this aim, 50 μM calf thymus DNA (ct-DNA) was incubated with different molar ratios (ri) of Pt(II) complexes **C7** and **C8** and stock solutions of their corresponding irradiation products **C7′** and **C8′** (see the Supporting Information, Figure S11). Interestingly, the CD spectra for both complexes showed an interaction before and after irradiation. In their nonirradiated forms, **C7** and **C8** displayed a similar behavior with a decrease in both positive and negative bands of ct-DNA (Figure S11a,b, respectively). Conversely, the irradiation products, **C7′** and **C8′**, showed a differentiated interaction between them. Whereas for **C7′**, the positive band remains almost unaltered with a considerable decrease in the negative band, for **C8′**, the positive band decreases, with the negative one being unaffected (Figure S11c,d, respectively). Hence, although the complexes seem to change the morphology of the DNA similarly upon incubation in their nonirradiated form, there is a different type of interaction when the complexes are irradiated: **C7′** causing a perturbation in the helicity of the DNA and **C8′** destabilizing the base stacking. Hypothetically, these different interactions should lead to different in vitro activities toward tumor cells.

Since the target molecule for most Pt drugs is primarily the nuclear DNA, once we assessed binding by CD, a specific determination of Pt in DNA was conducted by extracting DNA from the exposed A2780 cells. Pt concentration was normalized to the DNA concentration, which was initially measured spectrophotometrically at 260 nm. Figure S11e shows the obtained percentage of Pt bound to DNA for the A2780 cell line exposed to **C7** and **C8** complexes at their respective IC_50_. In the light of these results, we concluded that Pt incorporation into DNA is much more efficient in the case of compound **C7**, about 2-fold compared to **C8**. This result could also explain why complex **C7** is capable of exerting a greater cytotoxicity than **C8**.

#### Apoptosis Detection

**Figure 7:**
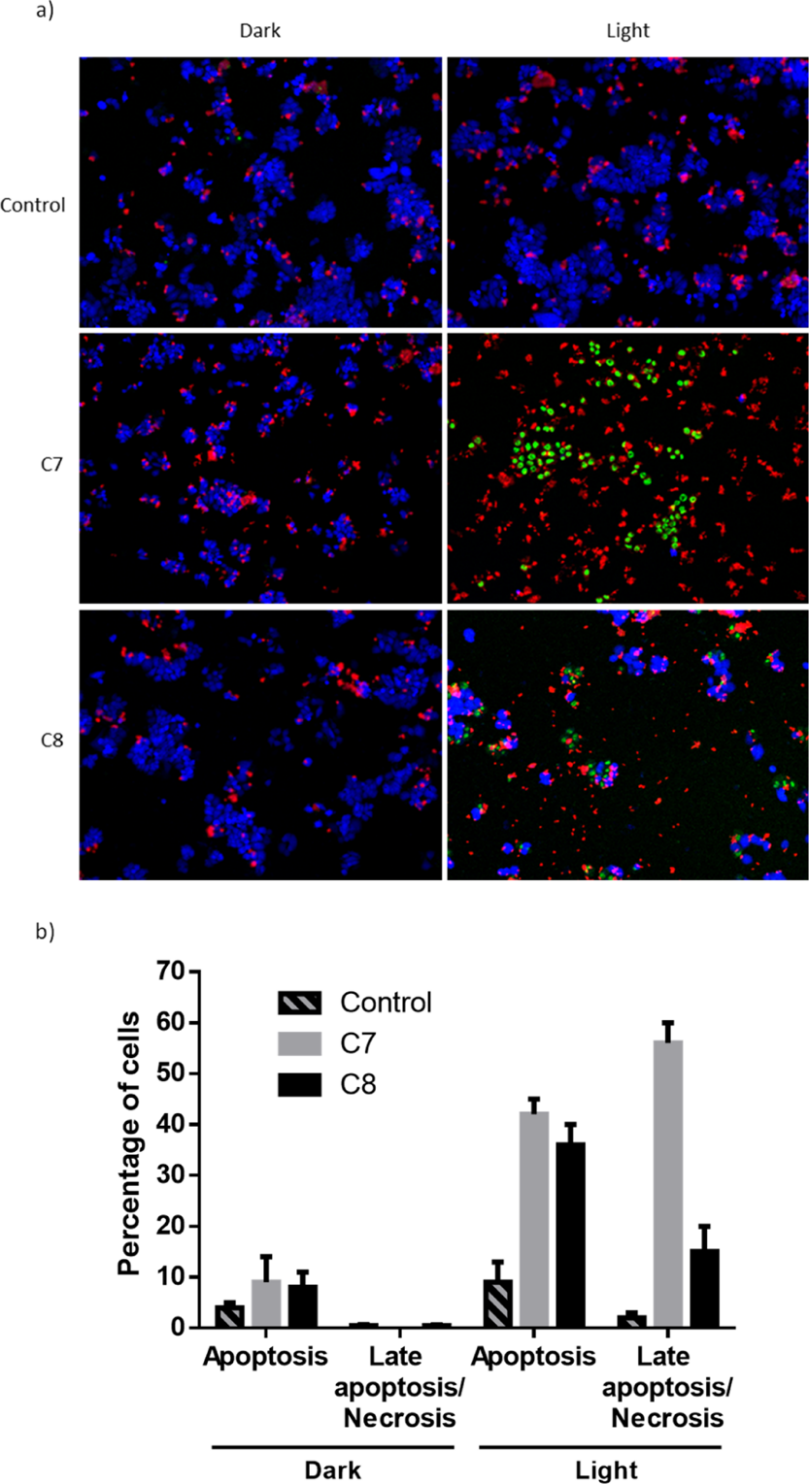
(a) Necrosis and apoptosis assays of A2780 cells untreated (control dark), treated with light only (control light), 1.5 μM **C7** (**C7** dark), light irradiation in the presence of 1.5 μM **C7** (**C7** light), 5 μM **C8** (**C8** dark), and light irradiation in the presence of 5 μM **C8** (**C8** light). Healthy viable cells were stained with CytoCalcein Violet 450 (blue), late apoptotic/necrotic cells with DNA nuclear green DCS1 (green), and apoptotic cells with phosphatidylserine (red). (b) Representative histograms of stained cells. Results are shown as a percentage of total cells.

To date, different biological action mechanisms for Pt(II) PACT-based antitumor agents have been proposed.^29^ It is well-established that the biological action of Pt(II) is ultimately related with DNA binding between the water-activated drug and two adjacent guanine–cytosine base pairs,^30^ generating a Pt-DNA cross-link adduct which impedes cell propagation.^31^ Nevertheless, proteins can also be platinated, inducing direct cell damage and immune response activation, inducing a mitochondrial-reactive oxygen species (ROS) response.^32^ The damage in the mitochondrial membrane is enough to hamper apoptosis after cytochrome *c* release into the cytosol.^33^ In addition to apoptosis, Pt(II) PACT complexes could potentially lead to cell death via necrosis, with a combination of both apoptosis and necrosis involved in the final cell cytotoxic effect. Generally, the prevalent cell death type is dependent not only on the structure, intracellular localization, and concentration of the complex but also on the light dose applied and on cell origin. Thus, further biological investigations were warranted to gain insights into the molecular mechanisms of the complexes, which will provide helpful insights into the rational design of photoactivatable platinum-based antitumor complexes. To verify the mechanism of cell death induced by **C7** and **C8**, A2780 cells were assayed for apoptosis or late apoptosis/necrosis detection. Cells were treated for 24 h with **C7** and **C8** at their corresponding IC_50_ after light activation (1.5 and 5 μM, respectively) and then photoactivated (light dose of 32 J/cm^2^). After a final 72 h of incubation, cells were stained with an apoptosis/necrosis assay kit which senses the phosphatidylserine exposure on cells as a hallmark of apoptosis. Cells were examined under a fluorescent microscope to differentiate between apoptotic (red), late apoptotic/necrotic (green), and healthy (blue) cells (Figure 7). The controls (untreated photoactivated and nonphotoactivated cells) displayed a physiological level of ca. 10% of apoptotic cells but exhibited no late apoptosis/necrosis. Similar results were detected for complexes **C7** and **C8** without photoactivation. More interestingly, after photoactivation, the proportion of apoptotic cells among the A2780 cells treated with compound **C7** rose to 42% at its IC_50_, 1.5 μM (Figure 7). Under the same conditions, compound **C8** induced apoptosis in 36% of cells. The observed results also showed a significantly increased level of late/apoptotic/necrotic cells after photoactivation of the complexes, with a special incidence in the **C7** compound (56%).

The obtained data evaluating the cellular death mechanism induced by complexes **C7** and **C8** present promising cancer-targeting properties by activation of apoptotic pathways. As shown, the analyzed carcinoma cell line presented apoptosis only after photoactivation, allowing a better selectivity toward irradiated cancer cells, with minimal effect over those kept in the dark.

#### ROS Generation Study

**Figure 8:**
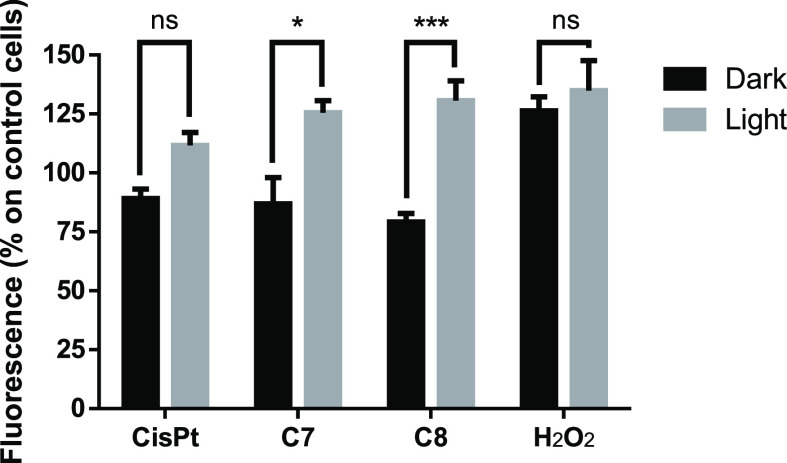
ROS formation measured with the DCFDA assay in A2780 cells for cisplatin (CisPt) and complexes **C7** and **C8** at the corresponding IC_50_ values for each complex (Table 1) after treatment for 4 h. Results are represented as the percentage over untreated cells. H_2_O_2_ (100 μM) was used as the positive control.

As previously mentioned, Pt(II) PACT-based complexes can be related with an oxidative-dependent mechanism to trigger cell death. In order to detect the formation of intracellular ROS in A2780 cells in the presence of the complexes while observing the effect of the photoactivation, the 2′,7′-dichlorofluorescein diacetate (DCFDA) assay was performed. In brief, DCFDA is cleaved and oxidized by intracellular esterases and ROS, generating the fluorescent compound dichlorofluorescein. Treatment with both complexes followed by light activation resulted in an elevation in cellular ROS release when compared to control cells (3-fold increase), highlighting the significant ROS production capabilities of these two photoactivatable complexes (Figure 8). On the contrary, cells treated with complexes but kept in the dark or the control molecules used (cisplatin and H_2_O_2_) in the dark or after light activation did not show a significant difference in ROS release. These results are in concordance with the significant cytotoxicity levels previously found for these complexes (Table 3).

Thus, upon photoactivation, both complexes **C7** and **C8** are capable of inducing ROS production and cell death through apoptosis. Having low cytotoxicity in the dark, the present complexes exert a multimechanistic chemotherapeutic effect that may serve for a targeted cancer chemotherapy.

## Summary and Conclusions

Pursuing the improvement of our previous Pt(II) complexes already published^11^ in reference to the solubility and the photoresponse wavelength, in this study, we have succeeded in synthesizing, characterizing, and studying the photochemical properties of eight new semisquaraine-type Pt(II) complexes. The synthesis of semisquaraine ligands has been carried out by means of a new straightforward approach. Also, XRD analysis of ligand **L2** revealed a 1,2-semisquaraine-type constitution which has been consequently extended to all the ligands presented. DFT calculations have allowed us to propose the *trans* configuration of the platinum center. Importantly, by structural modification of the ligands, the corresponding complexes are photoactive at wavelengths such that they allow their activation in cell cultures. Photodegradation processes have been studied. Water-soluble complexes **C7** and **C8** present quantum yields increased with respect to the corresponding free ligands **L7** and **L8**. By means of NMR techniques, it has been also possible to determine the structure of photoproducts of the presented semisquaraines, concluding that what is generated are butenolide-type derivatives, thus justifying the changes observed before and after irradiation.

Among all the complexes, two have been selected for their biological evaluation, mainly because both have an excellent solubility in physiological media. Thus, complexes **C7** and **C8** have been demonstrated to behave as promising photoactivatable compounds in the presented cancer cell lines, showing a potential amelioration over cisplatin-resistance in A2780cis cells. Upon photoactivation, both complexes were capable of inducing a higher cytotoxic effect on the tested cells compared with nonphotoactivated compounds. Among the observed results, it is remarkable to note that **C7** showed a PI > 50 in HeLa cells and **C8** showed a PI > 40 in A2780 cells, being also effective over cisplatin-resistant A2780cis cells (PI = 7 and PI = 4, respectively), a finding consistent with the previously reported Pt(II)-based compounds. Moreover, after photoactivation, these complexes were also shown to interact with DNA (PACT-likely) and further cause ROS production (PDT-likely) and ultimately cell death through apoptosis in the A2780 cancer cell line. We envision this dual effect^34^ as an opportunity to overcome one of the current PDT drawbacks, as PDT is based on the conversion of ground-state triplet into excited-state singlet oxygen. Photo-activated platinum complexes would not require the presence of oxygen, a potential advantage since tumor cells are often hypoxic.

Having a low cytotoxicity in the dark, complexes **C7** and **C8** may serve as a prodrug that upon photoactivation in a targeted cancer tissue, exert a potent multimechanistic chemotherapeutic effect. The controlled photoactivation of these platinum complexes could allow a targeted cell death in regions of cancer growth and avoidance of toxic effects on normal cells, and thus may be considered a potential lead molecule for a targeted cancer chemotherapy.

In all, we have been able to design, synthesize, and characterize new platinum complexes that have been shown to have a powerful cytotoxic activity, solving problems concerning their solubility and their photoresponse range. Ultimately, we hope that this study contributes to the development of new cytotoxic Pt(II) photoactive photocages. More studies in this line continue to be completed.

## Experimental Section

### Materials

K_2_PtCl_4_ was purchased from Strem Chemicals. Different organic reagents used for ligands synthesis were purchased from Sigma-Aldrich or Alfa Aesar. Organic solvents were dried before use when required. Compounds **9**,^14^**10**,^13^**14**,^15^**15**,^16^ and **16**(17) were synthesized according to previous descriptions.

### Synthesis of Ligands

#### 3-(1-{3-[Methylthio]propyl}-1*H*-pyrrol-2-yl)-4-({3-[methylthio]propyl}amino)cyclobut-3-ene-1,2-dione, **L1**

A solution of **9** (521.3 mg, 3.45 mmol) in dry THF (8 mL) was added dropwise to a solution of **10** (527.7 mg, 3.40 mmol) in dry THF (12 mL). The resulting pink solution was stirred at room temperature under an argon atmosphere overnight. After this time, **11** (374 μL, 3.41 mmol) and DIPEA (1.2 mL, 6.9 mmol) were added. The resulting mixture was stirred at room temperature for 5 h. Then, 120 mL of CH_2_Cl_2_ was added, and the organic layer was washed with H_2_O (3 × 60 mL), dried over anhydrous Na_2_SO_4_, and filtered. The solvent was removed under reduced pressure, and the crude was purified by column chromatography (silica gel, hexane to hexane/EtOAc, 2:3) to give **L1** (813.0 mg, 2.40 mmol, 71%). Recrystallization in MeOH gave pure **L1** (612.3 mg, 1.81 mmol, 53%) as a yellow powder. mp 116–119 °C (from MeOH). IR (ATR): 3281, 2955, 2916, 1769, 1613, 1580, 1530, 1474, 1435, 1420, 1384, 1353, 1257, 1226, 1157, 1126, 1093, 1063, 1048, 1021, 983, 955, 906, 868, 853, 810 cm^–1^. ^1^H NMR (400 MHz, CDCl_3_): δ 6.98 (dd, *J* = 2.6 Hz, 1.5 Hz, 1H), 6.58–6.47 (m, 1H), 6.47–6.42 (m, 1H), 6.32 (dd, *J* = 4.0 Hz, 2.6 Hz, 1H), 4.63 (t, *J* = 7.2 Hz, 2H), 4.03–3.96 (q, *J* = 6.7 Hz, 2H), 2.66 (t, *J* = 6.7 Hz, 2H), 2.42 (t, *J* = 7.2 Hz, 2H), 2.14 (s, 3H), 2.07 (s, 3H), 2.03 (qn, *J* = 6.7 Hz, 2H), 2.00 (qn, *J* = 7.2 Hz, 2H). ^13^C NMR (101 MHz, CDCl_3_): δ 189.4, 185.7, 177.1, 157.4, 128.7, 123.8, 112.0, 111.0, 48.8, 45.1, 31.9, 31.5, 31.0, 29.4, 15.6, 15.5. HRMS (ESI^+^): calcd for [C_16_H_22_N_2_O_2_S_2_]: 339.1195 [M + H]^+^, 361.1015 [M + Na]^+^; found, 339.1205 [M + H]^+^, 361.1025 [M + Na]^+^. UV (DMSO) λ_max_, nm (ε, M^–1^ cm^–1^): 281 (1.21 × 10^4^), 367 (2.50 × 10^4^).

#### 1-(3-{Methylthio}propyl)-1*H*-pyrrole-2-carbaldehyde, **13**

A solution of **12** (2.13 g, 22.4 mmol) in anhydrous DMF (10 mL) was added dropwise to a suspension of NaH (60% in mineral oil, 887 mg, 22.2 mmol) in anhydrous DMF (10 mL) previously cooled to 0 °C. The resulting suspension was stirred under argon for 30 min, and (3-bromopropyl)(methyl)sulfane^35^ (3.73 g, 22.1 mmol) was added dropwise. The suspension was stirred at room temperature for 4 h and then poured into water (40 mL). The mixture was extracted with Et_2_O (3 × 20 mL), and each extract was washed with water (3 × 10 mL). The combined organic layers were dried over anhydrous Na_2_SO_4_ and filtered, and the solvent was removed under reduced pressure to give a yellow oil. The product was purified by column chromatography (silica gel, hexane/Et_2_O, 9:1 to hexane/Et_2_O, 4:1) to give **13** (3.23 g, 17.6 mmol, 80%) as a yellow oil. IR (ATR): 3105, 2915, 2801, 2721, 1657, 1526, 1480, 1403, 1369, 1321, 1264, 1217, 1078, 1031, 957, 887 cm^–1^. ^1^H NMR (400 MHz, CDCl_3_): δ 7.26 (d, *J* = 1.1 Hz, 1H), 6.99–6.97 (ddd, *J* = 2.5 Hz, 1.7 Hz, 1.1 Hz, 1H), 6.94 (dd, *J* = 4.0 Hz, 1.7 Hz, 1H), 6.22 (dd, *J* = 4.0 Hz, 2.5 Hz, 1H), 4.42 (t, *J* = 6.8 Hz, 2H), 2.43 (t, *J* = 6.8 Hz, 2H), 2.09 (s, 3H), 2.05 (qn, *J* = 6.8 Hz, 2H). ^13^C NMR (101 MHz, CDCl_3_): δ 179.4, 131.8, 131.4, 125.2, 109.7, 47.7, 30.9, 30.1, 15.5. HRMS (ESI^+^): calcd for [C_9_H_13_NOS]: 184.0791 [M + H]^+^, 206.0610 [M + Na]^+^; found, 184.0795 [M + H]^+^, 206.0616 [M + Na]^+^.

#### (*E*)-2-(4-Methoxystyryl)-1-(3-{methylthio}propyl)-1*H*-pyrrole, **17**

A solution of phosphonate **14** (749 mg, 2.90 mmol) in anhydrous DMF (2.5 mL) was added to a suspension of NaH (60% in mineral oil, 273 mg, 6.83 mmol) in anhydrous DMF (2.5 mL) previously cooled to 0 °C under an argon atmosphere. Then, a solution of 13 (533 mg, 2.91 mmol) in anhydrous DMF (1 mL) was added, and the cooled mixture was heated progressively until 50 °C for 1 h. After this time, the mixture was cooled to 0 °C, 12 mL of H_2_O was added dropwise, and the product was extracted with EtOAc (3 × 12 mL). The combined organic extracts were dried over anhydrous Na_2_SO_4_ and filtered, the solvent was removed under reduced pressure, and the crude product was purified by column chromatography (silica gel, hexane/Et_2_O, 9:1) to give **17** (787.7 mg, 2.74 mmol, 94%) as a yellow oil. IR (ATR): 2913, 2834, 1737, 1628, 1604, 1574, 1508, 1474, 1440, 1358, 1299, 1278, 1243, 1173, 1125, 1109, 1077, 1031, 951, 847, 814 cm^–1^. ^1^H NMR (360 MHz, CD_3_OD): δ 7.43–7.39 (m, 2H), 6.98 (dt, *J* = 16.1 Hz, 0.7 Hz, 1H), 6.90–6.85 (m, 2H), 6.81 (d, *J*_trans_ = 16.1 Hz, 1H), 6.70 (dd, *J* = 2.7 Hz, 1.7 Hz, 1H), 6.39 (ddd, *J* = 3.8 Hz, 1.7 Hz, 0.7 Hz, 1H), 6.06 (ddd, *J* = 3.8 Hz, 2.7 Hz, 0.7 Hz, 1H), 4.14 (t, *J* = 6.8 Hz, 2H), 3.79 (s, 3H) 2.42 (t, *J* = 6.8 Hz, 2H), 2.05 (s, 3H), 1.98 (qn, *J* = 6.8 Hz, 2H). ^13^C NMR (91 MHz, CD_3_OD): δ 160.3, 132.8, 132.2, 128.1, 126.3, 123.3, 116.4, 115.1, 109.1, 106.7, 55.7, 45.8, 31.7, 31.7, 15.3. HRMS (ESI^+^): calcd for [C_17_H_21_NOS]: 288.1417 [M + H]^+^, 310.1236 [M + Na]^+^; found, 288.1423 [M + H]^+^, 310.1239 [M + Na]^+^.

#### (*E*)-1-(3-{Methylthio}propyl)-2-(3,4,5,-trimethoxystyryl)-1*H*-pyrrole, **18**

Phosphonate **15** (774.9 mg, 2.4 mmol) was cooled to 0 °C under an argon atmosphere. Then, anhydrous DMF (5 mL), NaH (60% dispersion in mineral oil, 203.7 mg, 5.1 mmol), and 13 (452.0 mg, 2.5 mmol) were added. The reaction mixture was stirred at room temperature for 1 h and then heated to 100 °C for 3 h. After this time, 25 mL of ice/water were added, and the product was extracted with EtOAc (2 × 30 mL). The combined organic extracts were dried over anhydrous Na_2_SO_4_ and filtered, the solvent was removed under reduced pressure, and the residue was purified by column chromatography (silica gel, hexane/EtOAc, 8:2) to give 18 (696.9 mg, 2.0 mmol, 83%) as a yellow oil. The oil was cooled in the fridge to give a pale-yellow solid. mp 66–68 °C (from CH_2_Cl_2_). IR (ATR): 2921, 2852, 1737, 1575, 1505, 1460, 1444, 1425, 1412, 1354, 1337, 1315, 1297, 1277, 1232, 1188, 1116, 996, 950, 856, 816 cm^–1^. ^1^H NMR (400 MHz, CD_3_OD): δ 7.09 (dt, *J* = 16.0 Hz, 0.6 Hz, 1H), 6.81 (d, *J*_trans_ = 16.0 Hz, 1H), 6.80 (s, 2H), 6.73 (dd, *J* = 2.7 Hz, 1.7 Hz, 1H), 6.44 (ddd, *J* = 3.7 Hz, 2.7 Hz, 0.6 Hz, 1H), 6.08 (ddd, *J* = 3.7 Hz, 2.7 Hz, 0.6 Hz, 1H), 4.17 (t, *J* = 6.7 Hz, 2H), 3.87 (s, 6H), 3.76 (s, 3H), 2.44 (t, *J* = 6.7 Hz, 2H), 2.05 (s, 3H), 1.99 (qn, *J* = 6.7 Hz, 2H). ^13^C NMR (101 MHz, CD_3_OD): δ 154.6, 138.3, 135.7, 132.5, 126.4, 123.7, 118.2, 109.4, 107.3, 104.3, 61.2, 56.6, 45.6, 31.8, 15.3. HRMS (ESI^+^): calcd for [C_19_H_25_NO_3_S]: 348.1628 [M + H]^+^, 370.1447 [M + Na]^+^; found, 348.1636 [M + H]^+^, 370.1458 [M + Na]^+^.

#### (*E*)-3-(5-{4-Methoxystyryl}-1-{3-[methylthio]propyl}-1*H*-pyrrol-2-yl)-4-({3-[methylthio]propyl}amino)cyclobut-3-ene-1,2-dione, **L2**

A solution of **17** (2.73 g, 9.5 mmol) in CH_2_Cl_2_ (90 mL) was added dropwise to a mixture of a solution of **9** (1.43 g, 9.5 mmol) in CH_2_Cl_2_ (200 mL) and H_2_O (50 mL). The organic layer turned dark red, and the resulting mixture was stirred at room temperature under an argon atmosphere for 1 h. After this time, a solution of thioamine **11** (1.1 mL, 9.8 mmol) and DIPEA (3.3 mL, 18.9 mmol) in CH_2_Cl_2_ (40 mL) was added, and the mixture was stirred for 30 min. Then, the organic layer was separated, dried over anhydrous Na_2_SO_4_, and filtered. The resulting filtrate was cooled to 0 °C, and Et_2_O was added. A yellow solid precipitated, which was filtered and washed with Et_2_O to give pure **L2** (3.19 mg, 6.8 mmol, 72%) as a yellow powder. mp 197–200 °C (from CH_2_Cl_2_/Et_2_O). IR (ATR): 3247, 2910, 1766, 1702, 1601, 1562, 1535, 1509, 1459, 1425, 1390, 1368, 1348, 1294, 1253, 1202, 1176, 1137, 1110, 1060, 1028, 963, 814, 799, 765 cm^–1^. ^1^H NMR (400 MHz, CDCl_3_): δ 7.48–7.43 (m, 2H), 7.04 (d, *J*_trans_ = 16.0 Hz, 1H), 6.98 (d, *J*_trans_ = 16.0 Hz, 1H), 6.93–6.88 (m, 2H), 6.67 (d, *J* = 4.3 Hz, 1H), 6.47 (d, *J* = 4.3 Hz, 1H), 6.44 (t, *J* = 6.6 Hz, 1H), 4.76 (t, *J* = 7.3 Hz, 2H), 4.02 (q, *J* = 6.6 Hz, 2H), 3.84 (s, 3H), 2.68 (t, *J* = 6.6 Hz, 2H), 2.52 (t, *J* = 7.3 Hz, 2H), 2.15 (s, 3H), 2.07 (s, 3H), 2.04 (qn, *J* = 6.6 Hz, 2H), 2.00 (qn, *J* = 7.3 Hz, 2H). ^13^C NMR (101 MHz, CDCl_3_): δ 188.5, 185.4, 176.6, 159.9, 156.6, 139.2, 130.9, 129.7, 128.0, 125.0, 114.4, 113.6, 112.5, 109.1, 55.5, 45.2, 45.0, 32.0, 31.3, 31.2, 29.3, 15.7, 15.6. HRMS (ESI^+^): calcd for [C_25_H_30_N_2_O_3_S_2_]: 471.1771 [M + H]^+^, 493.1590 [M + Na]^+^; found, 471.1781 [M + H]^+^, 493.1603 [M + Na]^+^. UV (DMF) λ_max_, nm (ε, M^–1^ cm^–1^): 303 (1.69 × 10^4^), 431 (7.32 × 10^4^), 447 (7.74 × 10^4^).

#### (*E*)-3-(1-{3-[Methylthio]propyl}-5-{3,4,5-trimethoxystyryl}-1*H*-pyrrol-2-yl)-4-({3-[methylthio]propyl}amino)cyclobut-3-ene-1,2-dione, **L4**

A solution of **18** (1.04 g, 3.0 mmol) in CH_2_Cl_2_ (40 mL) was added dropwise to a mixture of a solution of **9** (456 mg, 3.0 mmol) in CH_2_Cl_2_ (45 mL) and H_2_O (30 mL). The organic layer turned dark red, and the resulting mixture was stirred at room temperature under an argon atmosphere for 1.5 h. After this time, a solution of **11** (340 μL, 3.0 mmol) and DIPEA (1.1 mL, 6.3 mmol) in CH_2_Cl_2_ (5 mL) was added, and the mixture was stirred for 2 h. Then, the organic layer was separated, dried over anhydrous Na_2_SO_4_, and filtered. The solvent of the resulting filtrate was removed partially under reduced pressure, and then the mixture was cooled to 0 °C, and Et_2_O was added. An orange solid precipitated, which was filtered and washed with Et_2_O to give pure **L4** (925 mg, 1.7 mmol, 57%) as an orange powder. mp 149–152 °C (from CH_2_Cl_2_/Et_2_O). IR (ATR): 3337, 2912, 2836, 1760, 1694, 1581, 1526, 1505, 1464, 1444, 1423, 1342, 1295, 1271, 1245, 1230, 1128, 1043, 1003, 951, 805, 769 cm^–1^. ^1^H NMR (400 MHz, CDCl_3_): δ 7.08 (d, *J*_trans_ = 16.0 Hz, 1H), 6.99 (d, *J*_trans_ = 16.0 Hz, 1H), 6.76 (s, 2H), 6.69 (d, *J* = 4.3 Hz, 1H), 6.51 (t, *J* = 6.6 Hz, 1H), 6.49 (d, *J* = 4.3 Hz, 1H), 4.79 (t, *J* = 6.8 Hz, 2H), 4.01 (q, *J* = 6.6 Hz, 2H), 3.91 (s, 6H), 3.87 (s, 3H), 2.67 (t, *J* = 6.6 Hz, 2H), 2.54 (t, *J* = 6.8 Hz, 2H), 2.15 (s, 3H), 2.07 (s, 3H), 2.07–1.97 (m, 4H). ^13^C NMR (101 MHz, CDCl_3_): δ 186.6, 185.4, 176.8, 156.5, 153.6, 138.6, 138.5, 132.7, 131.0, 125.2, 115.3, 112.5, 109.3, 103.7, 61.1, 56.3, 45.2, 44.8, 32.0, 31.4, 31.3, 29.3, 15.8, 15.6. HRMS (ESI^+^): calcd for [C_27_H_34_N_2_O_5_S_2_]: 531.1982 [M + H]^+^, 553.1801 [M + Na]^+^; found, 531.1996 [M + H]^+^, 553.1814 [M + Na]^+^. UV (DMF) λ_max_, nm (ε, M^–1^ cm^–1^): 306 (1.29 × 10^4^), 429 (5.93 × 10^4^), 448 (6.20 × 10^4^).

#### (*E*)-3-(1-{3-[Methylthio]propyl}-5-{3,4,5-tris[2-(2-{2-methoxyethoxy}ethoxy)ethoxy]styryl}-1*H*-pyrrol-2-yl)-4-({3-[methylthio]propyl}amino)cyclobut-3-ene-1,2-dione, **L7**

A suspension of NaH (60% dispersion in mineral oil, 51.7 mg, 1.29 mmol) in anhydrous DMF (0.5 mL) was cooled to 0 °C under an argon atmosphere. Then, a solution of diethyl (3,4,5-tris{2-[2-(2-methoxyethoxy)ethoxy]ethoxy}benzyl)phosphonate^36^ (431.6 mg, 0.60 mmol) in anhydrous DMF (0.7 mL) was added dropwise. The reaction mixture was stirred at 0 °C for 30 min, then a solution of 13 (112.8 mg, 0.62 mmol) in anhydrous DMF (0.7 mL) was added, and the mixture was heated at 100 °C for 16 h. After this time, the mixture was cooled to 0 °C, and H_2_O (5 mL) was added dropwise. The aqueous phase was extracted with EtOAc (3 × 5 mL). Each organic phase was washed with brine (3 × 5 mL). The combined organic extracts were dried over anhydrous Na_2_SO_4_ and filtered. The solvent was removed under reduced pressure, and the residue was purified by column chromatography (silica gel, EtOAc to EtOAc/MeOH, 95:5) to give crude **19** (341.2 mg) as a dark brown oil, which was used in the next step without further purification.

A solution of crude **19** (238.8 mg, 0.32 mmol) in CH_2_Cl_2_ (3 mL) was added dropwise to a mixture of a solution of dichlorocyclobutenedione **9** (49.9 mg, 0.33 mmol) in CH_2_Cl_2_ (7 mL) and H_2_O (2 mL). The organic layer turned dark red, and the mixture was stirred at room temperature for 1 h. After this time, a solution of **11** (36 μL, 0.32 mmol) and DIPEA (113 μL, 0.64 mmol) in CH_2_Cl_2_ (1 mL) was added. The resulting mixture was stirred at rt for 90 min. Then, the organic layer was dried over anhydrous Na_2_SO_4_ and filtered. The solvent was removed under reduced pressure, and the residue was purified by column chromatography (silica gel, EtOAc to EtOAc/MeOH, 95:5) to give pure **L7** (245.6 mg, 0.26 mmol, 62% over 2 steps) as a red-orange oil. The oil was kept in the freezer turning into a brown sticky solid. mp 46–49 °C (from CH_2_Cl_2_). IR (ATR): 2919, 2876, 1766, 1714, 1596, 1533, 1504, 1466, 1431, 1341, 1295, 1270, 1109, 952, 854 cm^–1^. ^1^H NMR (400 MHz, CDCl_3_): δ 7.00 (d, *J*_trans_ = 16.0 Hz, 1H), 6.92 (d, *J*_trans_ = 16.0 Hz, 1H), 6.75 (s, 2H), 6.66 (d, *J* = 4.3 Hz, 1H), 6.53 (t, *J* = 6.5 Hz, 1H), 6.49 (d, *J* = 4.3 Hz, 1H), 4.75 (t, *J* = 6.9 Hz, 2H), 4.22–4.13 (m, 6H), 3.99 (q, *J* = 6.5 Hz, 2H), 3.89–3.49 (m, 30H), 3.36 (s, 3H), 3.36 (s, 6H), 2.66 (t, *J* = 6.5 Hz, 2H), 2.51 (t, *J* = 6.9 Hz, 2H), 2.13 (s, 3H), 2.06 (s, 3H), 2.05–1.95 (m, 4H). ^13^C NMR (100 MHz, CDCl_3_): δ 188.7, 185.4, 176.8, 156.5, 153.0, 139.1, 138.6, 132.5, 130.8, 125.2, 115.3, 112.6, 109.4, 106.5, 72.6, 72.1, 70.9, 70.8, 70.7, 70.6, 69.9, 69.1, 59.1, 45.1, 44.9, 31.9, 31.4, 31.3, 29.5, 15.8, 15.6. HRMS (ESI^+^): calcd for [C_45_H_70_N_2_O_14_S_2_]: 927.4341 [M + H]^+^, 949.4161 [M + Na]^+^; found, 927.4326 [M + H]^+^, 949.4164 [M + Na]^+^. UV (H_2_O) λ_max_, nm (ε, M^–1^ cm^–1^): 212 (2.15 × 10^4^), 235 (1.70 × 10^4^), 308 (1.03 × 10^4^), 432 (4.06 × 10^4^).

#### (*E*)-3-(5-{4-Hydroxystyryl}-1-{3-[methylthio]propyl}-1*H*-pyrrol-2-yl)-4-({3-[methylthio]propyl}amino)cyclobut-3-ene-1,2-dione, **L3**

Boron tribromide (1 M in dichloromethane, 10 mL, 10.0 mmol) was added dropwise to a suspension of **L2** (533 mg, 1.1 mmol) in anhydrous CH_2_Cl_2_ (10 mL) at −10 °C (ice/acetone bath). The mixture was stirred under an argon atmosphere at 0 °C for 2 h. After this time, H_2_O (20 mL) was added dropwise to the cooled mixture, and an orange solid precipitated. The resulting mixture was basified with a NaHCO_3_ saturated solution (55 mL) and stirred for 1 h. Then, the organic phase was removed under reduced pressure, and the formed precipitate was filtrated and washed with H_2_O and Et_2_O. The resulting solid was digested with Et_2_O and then filtrated to afford pure **L3** (439.8 mg, 0.96 mmol, 87%) as a brown solid. mp 192–196 °C (from CH_2_Cl_2_). IR (ATR): 3249, 2916, 1766, 1705, 1568, 1536, 1511, 1459, 1428, 1386, 1368, 1342, 1296, 1270, 1253, 1217, 1173, 1138, 1106, 1061, 1044, 952, 929, 850, 816, 760 cm^–1^. ^1^H NMR (400 MHz, DMSO-*d*_6_): δ 9.65 (s, 1H), 8.57 (t, *J* = 6.6 Hz, 1H), 7.59–7.43 (m, 2H), 7.13 (d, *J* = 16.1 Hz, 1H), 7.05 (d, *J* = 16.1 Hz, 1H), 6.96 (d, *J* = 4.4 Hz, 1H), 6.82 (d, *J* = 4.4 Hz, 1H), 6.80–6.76 (m, 2H), 4.72 (t, *J* = 7.2 Hz, 2H), 3.77 (q, *J* = 6.6 Hz, 2H), 2.52 (t, *J* = 6.6 Hz, 2H), 2.38 (t, *J* = 7.2 Hz, 2H), 2.05 (s, 3H), 1.99 (s, 3H), 1.90 (qn, *J* = 6.6 Hz, 2H), 1.82 (qn, *J* = 7.2 Hz, 2H). ^13^C NMR (101 MHz, DMSO-*d*_6_): δ 188.9, 184.3, 175.6, 157.6, 155.4, 138.7, 130.2, 128.0, 124.5, 115.6, 114.5, 112.8, 108.6, 44.2, 43.5, 31.3, 30.2, 30.0, 29.9, 14.6. HRMS (ESI^+^): calcd for [C_24_H_28_N_2_O_3_S_2_]: 457.1614 [M + H]^+^, 479.1434 [M + Na]^+^; found, 457.1604 [M + H ]^+^, 479.1432 [M + Na]^+^. UV (DMSO) λ_max_, nm (ε, M^–1^ cm^–1^): 306 (1.56 × 10^4^), 436 (5.60 × 10^4^), 452 (5.77 × 10^4^).

#### (*E*)-3-(1-{3-[Methylthio]propyl}-5-{3,4,5-trihydroxystyryl}-1*H*-pyrrol-2-yl)-4-({3-[methylthio]propyl}amino)cyclobut-3-ene-1,2-dione, **L5**

Boron tribromide (1.0 M in dichloromethane, 14 mL, 14.0 mmol) was added dropwise to a suspension of 22 (482 mg, 0.91 mmol) in anhydrous CH_2_Cl_2_ (14 mL) at −10 °C (ice/acetone bath). The mixture was stirred under an argon atmosphere at 0 °C for 1 h. After this time, 30 mL of H_2_O was added, and an orange solid precipitated. The resulting mixture was basified with a NaHCO_3_ saturated solution (40 mL), and the solid was filtrated and washed with H_2_O and MeOH to give pure **L5** (436.6 mg, 0.89 mmol, 98%) as a dark orange solid. mp 215–218 °C (from CH_2_Cl_2_). IR (ATR): 3435, 3269, 2915, 1765, 1671, 1570, 1524, 1468, 1432, 1366, 1334, 1312, 1270, 1224, 1191, 1174, 1116, 1047, 997, 955, 860, 820, 767 cm^–1^. ^1^H NMR (400 MHz, DMSO-*d*_6_): δ 8.91 (s, 2H), 8.53 (t, *J* = 7.1 Hz, 1H), 8.50 (s, 1H), 6.98 (d, *J* = 15.9 Hz, 1H), 6.93 (d, *J* = 4.3 Hz, 1H), 6.89 (d, *J* = 15.9 Hz, 1H), 6.83 (d, *J* = 4.3 Hz, 1H), 6.56 (s, 2H), 4.72 (t, *J* = 7.1 Hz, 2H), 3.77 (q, *J* = 7.1 Hz, 2H), 2.53 (t, *J* = 7.1 Hz, 2H), 2.39 (t, *J* = 7.1 Hz, 2H), 2.06 (s, 3H), 2.01 (s, 3H), 1.90 (qn, *J* = 7.1 Hz, 2H), 1.83 (qn, *J* = 7.1 Hz, 2H). ^13^C NMR (101 MHz, DMSO-*d*_6_): δ 188.8, 184.4, 175.6, 155.4, 146.2, 138.6, 134.0, 131.1, 127.6, 124.5, 114.4, 112.7, 108.7, 105.9, 44.1, 43.5, 31.2, 30.2, 30.0, 29.9, 14.6. HRMS (ESI^+^): calcd for [C_24_H_28_N_2_O_5_S_2_]: 489.1512 [M + H]^+^, 511.1332 [M + Na]^+^; found, 489.1495 [M + H]^+^, 511.1324 [M + Na]^+^. UV (DMSO) λ_max_, nm (ε, M^–1^ cm^–1^): 311 (1.33 × 10^4^), 441 (6.93 × 10^4^), 455 (7.19 × 10^4^).

#### (*E*)-3-(5-{4-[2-(2-{2-Methoxyethoxy}ethoxy)ethoxy]styryl}-1-{3-[methylthio]propyl}-1*H*-pyrrol-2-yl)-4-({3-[methylthio]propyl}amino)cyclobut-3-ene-1,2-dione, **L6**

K_2_CO_3_ (50.1 mg, 0.36 mmol) was added to a suspension of **L3** (77.0 mg, 0.17 mmol) and 1-bromo-2-(2-(2-methoxyethoxy)ethoxy)ethane^37^ (53.3 mg, 0.23 mmol) in anhydrous MeCN (2 mL). The mixture was refluxed for 24 h under an argon atmosphere. After this time, the mixture was cooled to room temperature, K_2_CO_3_ was removed by filtration, and the product was recrystallized from hot methanol to give **L6** (45.3 mg, 0.08 mmol, 47%) as a yellow solid. mp 110–112 °C (from MeOH). IR (ATR): 3253, 2911, 2872, 1764, 1705, 1604, 1558, 1508, 1459, 1447, 1390, 1368, 1337, 1292, 1273, 1245, 1203, 1174, 1135, 1057, 1031, 951, 849, 815, 800, 758 cm^–1^. ^1^H NMR (400 MHz, CDCl_3_): δ 7.44–7.40 (m, 2H), 7.01 (d, *J* = 16.0 Hz, 1H), 6.95 (d, *J* = 16.0 Hz, 1H), 6.92–6.87 (m, 2H), 6.65 (d, *J* = 4.3 Hz, 1H), 6.63 (t, *J* = 6.9 Hz, 1H), 6.52 (d, *J* = 4.3 Hz, 1H), 4.75 (t, *J* = 7.0 Hz, 2H), 4.17–4.12 (m, 2H), 3.99 (q, *J* = 6.9 Hz, 2H), 3.89–3.84 (m, 2H), 3.77–3.71 (m, 2H), 3.71–3.63 (m, 4H), 3.57–3.52 (m, 2H), 3.37 (s, 3H), 2.66 (t, *J* = 6.9 Hz, 2H), 2.50 (t, *J* = 7.0 Hz, 2H), 2.13 (s, 3H), 2.05 (s, 3H), 2.07–1.95 (m, 4H). ^13^C NMR (101 MHz, CDCl_3_): δ 188.5, 185.4, 176.6, 159.1, 156.6, 139.2, 130.8, 129.9, 127.9, 125.0, 115.1, 113.7, 112.7, 109.1, 72.0, 71.0, 70.8, 70.7, 69.8, 67.6, 59.2, 45.1, 45.0, 31.9, 31.3, 31.2, 29.5, 15.6. HRMS (ESI^+^): calcd for [C_31_H_42_N_2_O_6_S_2_]: 603.2557 [M + H]^+^, 625.2376 [M + Na]^+^; found, 603.2564 [M + H]^+^, 625.2387 [M + Na]^+^. UV (DMF) λ_max_, nm (ε, M^–1^ cm^–1^): 304 (1.43 × 10^4^), 431 (6.03 × 10^4^), 448 (6.40 × 10^4^).

#### α-Methyl-ω-((*E*)-3-{5-[4-oxystyryl]-1-[3-(methylthio)propyl]-1*H*-pyrrol-2-yl}-4-{[3-(methylthio)propyl]amino}cyclobut-3-ene-1,2-dione)poly(oxyethane-1,2-diyl), **L8**

K_2_CO_3_ (421 mg, 3.0 mmol) was added to a suspension of **L3** (774 mg, 1.7 mmol) and α-methyl-ω-(methylsulfonate)poly(oxyethane-1,2-diyl)^38^ (*M*_w_ = 2078, 2.71 g, 1.3 mmol) in anhydrous MeCN (20 mL). The mixture was refluxed for 16 h under an argon atmosphere. After this time, the mixture was cooled to room temperature, K_2_CO_3_ was removed by filtration, and the solvent was removed under a reduced pressure. Then, CH_2_Cl_2_ (100 mL) and brine (60 mL) were added, and the aqueous layer was extracted with CH_2_Cl_2_ (3 × 20 mL). The combined organic layers were dried over anhydrous Na_2_SO_4_ and filtered, the solvent was removed under a reduced pressure, and the product was recrystallized from CH_2_Cl_2_/Et_2_O to afford **L8** (2.89 g, 1.2 mmol, 92%) as a yellow solid. mp 51–55 °C (from CH_2_Cl_2_/Et_2_O). IR (ATR): 2880, 2740, 1765, 1708, 1596, 1536, 1511, 1466, 1359, 1341, 1279, 1240, 1146, 1100, 1060, 960, 841 cm^–1^. ^1^H NMR (400 MHz, CDCl_3_): δ 7.44–7.38 (m, 2H), 7.00 (d, *J* = 16.1 Hz, 1H), 6.94 (d, *J* = 16.1 Hz, 1H), 6.91–6.86 (m, 2H), 6.69–6.61 (m, 3H), 6.54–6.48 (m, 1H), 4.74 (t, *J* = 7.0 Hz, 2H′), 4.15–4.09 (m, 2H), 3.96 (q, *J* = 6.8 Hz, 2H), 3.88–3.39 (m), 3.35 (s, 3H), 2.63 (t, *J* = 6.8 Hz, 2H), 2.48 (t, *J* = 7.0 Hz, 2H), 2.11 (s, 3H), 2.04 (s, 3H), 2.03–1.91 (m, 4H). ^13^C NMR (101 MHz, CDCl_3_): δ 188.4, 184.9, 176.1, 158.6, 156.0, 138.6, 130.1, 129.6, 127.5, 124.7, 115.9, 114.7, 113.4, 108.7, 72.3, 71.7, 70.6, 70.3, 69.4, 58.8, 44.5, 44.4, 31.0, 30.8, 29.7, 15.2. UV (H_2_O) λ_max_, nm (ε, M^–1^ cm^–1^): 192 (3.32 × 10^4^), 234 (1.38 × 10^4^), 308 (1.09 × 10^4^), 435 (3.39 × 10^4^).

#### (*E*)-3-(5-{4-[(*tert*-Butyldiphenylsilyl)oxy]styryl}-1-{3-[methylthio]propyl}-1*H*-pyrrol-2-yl)-4-({3-[methylthio]propyl}amino)cyclobut-3-ene-1,2-dione, **L20**

*tert*-Butyldiphenylsilyl chloride (0.35 mL, 1.32 mmol) was added dropwise to a solution of **L3** (274.4 mg, 0.60 mmol) in anhydrous DMF (0.25 mL). The resulting mixture was stirred under an argon atmosphere at room temperature for 18 h. After this time, 5 mL of H_2_O was added, and the product was extracted with EtOAc (3 × 5 mL). Each organic layer was washed with H_2_O (3 × 5 mL). The combined organic extracts were dried over anhydrous Na_2_SO_4_, and the solvent was removed under reduced pressure. The residue was purified by column chromatography (silica gel, hexane/EtOAc, 1:1) to give pure **L20** (225.2 mg, 0.32 mmol, 53%) as a yellow solid. mp 161–163 °C (from CH_2_Cl_2_). IR (ATR): 3308, 2931, 2857, 1768, 1706, 1601, 1563, 1538, 1507, 1472, 1426, 1390, 1370, 1254, 1174, 1139, 1108, 1057, 954, 912, 853, 819 cm^–1^. ^1^H NMR (400 MHz, CDCl_3_): δ 7.76–7.70 (m, 4H), 7.47–7.41 (m, 2H), 7.41–7.35 (m, 4H), 7.28–7.23 (m, 2H), 6.95 (d, *J* = 16.0 Hz, 1H), 6.90 (d, *J* = 16.0 Hz, 1H), 6.79–6.74 (m, 2H), 6.62 (d, *J* = 4.3 Hz, 1H), 6.58 (t, *J* = 6.6 Hz, 1H), 6.49 (d, *J* = 4.3 Hz, 1H), 4.72 (t, *J* = 7.1 Hz, 2H), 4.00 (q, *J* = 6.6 Hz, 2H), 2.66 (t, *J* = 6.6 Hz, 2H), 2.48 (t, *J* = 7.1 Hz, 2H), 2.13 (s, 3H), 2.03 (qn, *J* = 6.6 Hz, 2H), 2.02 (s, 3H), 1.97 (qn, *J* = 7.1 Hz, 2H), 1.11 (s, 9H). ^13^C NMR (101 MHz, CDCl_3_): δ 188.5, 185.4, 176.7, 156.6, 156.0, 139.2, 135.6, 132.8, 131.0, 130.1, 130.0, 127.9, 127.7, 125.0, 120.3, 113.7, 112.7, 109.1, 45.1, 45.0, 31.9, 31.3, 31.1, 29.5, 26.6, 19.6, 15.6, 15.6. HRMS (ESI^+^): calcd for [C_40_H_46_N_2_O_3_S_2_Si]: 695.2792 [M + H]^+^, 717.2611 [M + Na]^+^; found, 695.2794 [M + H ]^+^, 717.2617 [M + Na]^+^. UV (CHCl_3_) λ_max_, nm (ε, M^–1^ cm^–1^): 301 (1.21 × 10^4^), 431 (5.59 × 10^4^), 446 (5.75 × 10^4^).

#### (*E*)-3-(5-{4-Methoxystyryl}-1-{3-[methylthio]propyl}-1*H*-pyrrol-2-yl)-4-(methylamino)cyclobut-3-ene-1,2-dione, **L22**

A solution of **17** (88.1 mg, 0.31 mmol) in CH_2_Cl_2_ (3 mL) was added dropwise to a mixture of a solution of dichlorocyclobutenedione **9** (46.5 mg, 0.31 mmol) in CH_2_Cl_2_ (6 mL) and H_2_O (2 mL). The organic layer turned dark red, and the resulting mixture was stirred at room temperature under argon for 1 h. After this time, a solution of methylamine (33% wt in MeOH, 38 μL, 0.31 mmol) and DIPEA (105 μL, 0.60 mmol) in CH_2_Cl_2_ (2 mL) was added, and the mixture was stirred for 30 min. Then, the organic layer was separated, dried over anhydrous Na_2_SO_4_, and filtered. The resulting filtrate was cooled to 0 °C, and Et_2_O was added. An orange solid precipitated, which was filtered and washed with Et_2_O to give pure **L22** (45 mg, 0.11 mmol, 37%) as an orange powder. mp >220 °C (from CH_2_Cl_2_/Et_2_O). IR (ATR): 3293, 2910, 1766, 1699, 1569, 1507, 1450, 1426, 1386, 1294, 1251, 1172, 1130, 1110, 1026, 956, 816 cm^–1^. ^1^H NMR (400 MHz, DMSO-*d*_6_): δ 8.43 (q, *J* = 4.5 Hz, 1H), 7.61–7.53 (m, 2H), 7.17 (d, *J* = 16.2 Hz, 1H), 7.12 (d, *J* = 16.2 Hz, 1H), 7.00–6.92 (m, 2H), 6.89 (d, *J* = 4.3 Hz, 1H), 6.83 (d, *J* = 4.3 Hz, 1H), 4.75 (t, *J* = 7.4 Hz, 2H), 3.78 (s, 3H), 3.31 (d, *J* = 4.5 Hz, 3H), 2.38 (t, *J* = 7.4 Hz, 2H), 1.99 (s, 3H), 1.82 (qn, *J* = 7.4 Hz, 2H). ^13^C NMR (101 MHz, DMSO-*d*_6_): δ 189.4, 184.3, 175.9, 159.1, 155.1, 138.3, 129.6, 127.9, 124.7, 114.2, 114.0, 113.9, 108.8, 55.2, 44.2, 31.3, 29.9, 14.6. HRMS (ESI^+^): calcd for [C_22_H_24_N_2_O_3_S]: 397.1580 [M + H]^+^, 419.1400 [M + Na]^+^; found, 397.1567 [M + H]^+^, 419.1385 [M + Na]^+^. UV (DMF) λ_max_, nm (ε, M^–1^ cm^–1^): 302 (1.48 × 10^6^), 429 (6.08 × 10^6^), 446 (6.44 × 10^6^).

### Synthesis of Complexes

#### Complex **C**1

Ligand **L1** (86.7 mg, 0.26 mmol) and K_2_PtCl_4_ (105.6 mg, 0.25 mmol) were suspended in a mixture of MeOH and water (1:1, 4 mL). The mixture was stirred at room temperature under an argon atmosphere for 17 h. After this time, a brown solid precipitated. After filtration, the solid was washed with hot MeOH and water and then dried to afford **C1** as a brown powder (82.0 mg, 0.14 mmol, 56%). mp 173–178 °C (from MeOH/H_2_O, 1:1). IR (ATR): 3283, 2920, 1772, 1706, 1589, 1537, 1478, 1421, 1355, 1264, 1132, 1092, 970 cm^–1^. ^1^H NMR (400 MHz, DMSO-*d*_6_) as a mixture of products due to one Cl exchange: 8.67–8.51 (m), 7.31–7.16 (m), 6.92–6.81 (m), 6.40–6.26 (m), 4.61 (t, *J* = 6.9 Hz), 4.54 (t, *J* = 7.0 Hz), 3.86–3.71 (m), 2.59–2.50 (bs), 2.36–2.28 (bs), 1.93–1.81 (bs), 2.21–1.96 (bs). ^13^C NMR (101 MHz, DMSO-*d*_6_) as a mixture of products due to one Cl exchange: δ 190.0 + 189.8, 184.8 + 184.7, 176.0, 156.4, 156.2, 128.9, 123.3, 114.0–113.7, 110.6–110.4, 48.0, 47.6, 43.5, 43.1, 35.4, 35.3, 31.4, 30.2, 30.0, 29.9, 29.7, 28.4, 21.0, 20.6. HRMS (ESI^+^): calcd for [C_16_H_22_N_2_O_2_S_2_PtCl_2_]: 532.0688 [M–2Cl–H]^+^, 569.0444 [M–Cl]^+^, 605.0203 [M + H]^+^, 610.0827 [M–2Cl–H + DMSO]^+^, 647.0582 [M–Cl + DMSO]^+^; found, 532.0678 [M–2Cl–H]^+^, 569.0443 [M–Cl]^+^, 605.0206 [M + H]^+^, 610.0817 [M–2Cl–H + DMSO]^+^, 647.0583 [M–Cl + DMSO]^+^. EA calcd for C_16_H_22_N_2_O_2_S_2_PtCl_2_ (%): C, 30.87; H, 3.89; N, 4.50; S, 10.30 [M + H_2_O]. Found: C, 30.76; H, 3.49; N, 4.22; S, 10.12 [M + H_2_O]. UV (DMSO) λ_max_, nm (ε, M^–1^ cm^–1^): 276 (1.40 × 10^4^), 366 (2.25 × 10^4^), 445 (9.32 × 10^2^), 516 (2.30 × 10^2^).

#### Complex **C2**

A solution of K_2_PtCl_4_ (153.9 mg, 0.37 mmol) in H_2_O (5 mL) was added to a solution of **L2** (172.0 mg, 0.37 mmol) in THF (5 mL). The solution was stirred at room temperature under argon for 22 h. After this time, an orange solid precipitated. After filtration, the solid was washed with THF and water and dried to afford **C2** as an orange powder (262.2 mg, 0.36 mmol, 97%). mp 184–187 °C (from THF/H_2_O). IR (ATR): 3302, 2916, 1764, 1701, 1584, 1533, 1509, 1461, 1425, 1299, 1247, 1174, 1141, 1029, 957, 850, 816 cm^–1^. ^1^H NMR (400 MHz, DMF-*d*_7_): δ 8.75–8.51 (m), 7.75–755 (m), 7.40–7.65 (m), 5.06–4.63 (m), 4.23–3.76 (m), 3.41–2.83 (m), 2.69–2.02 (m). HRMS (ESI^+^): calcd for [C_25_H_30_N_2_O_3_S_2_PtCl_2_]: 737.0781 [M + H]^+^, 759.0601 [M + Na]^+^, 775.0339 [M + K]^+^; found, 737.0791 [M + H]^+^, 759.0607 [M + Na]^+^, 775.0353 [M + K]^+^. EA calcd for C_25_H_30_N_2_O_3_S_2_PtCl_2_ (%): C, 40.76; H, 4.11; N, 3.80; S, 8.70. Found: C, 40.96; H, 4.33; N, 3.51; S, 8.55. UV (DMF) λ_max_, nm (ε, M^–1^ cm^–1^): 305 (1.61 × 10^4^), 430 (6.18 × 10^4^), 446 (5.95 × 10^4^).

#### Complex **C3**

A solution of K_2_PtCl_4_ (89.2 mg, 0.22 mmol) in H_2_O (3 mL) was added to a solution of **L3** (95.1 mg, 0.21 mmol) in THF (3 mL). The solution was stirred at room temperature under argon for 15 h. After this time, a brown-red solid precipitated. After filtration, the solid was washed with H_2_O, MeOH, and THF and dried to afford **C3** as a brown-red powder (130.8 mg, 0.18 mmol, 86%). mp >200 °C (from THF/H_2_O). IR (ATR): 1736, 1691, 1579, 1532, 1510, 1461, 1421, 1267, 1168, 1138, 1044, 955, 850, 810 cm^–1^. ^1^H NMR (400 MHz, DMSO-*d*_6_): δ 9.65 (s), 8.70–8.51 (m), 7.59–7.41 (m), 7.22–6.62 (m), 4.92–4.63 (m), 3.91–3.68 (m), 3.27–3.03 (m), 2.70–2.24 (m), 2.15–1.70 (m). MS (ESI^+^): calcd for [C_24_H_28_N_2_O_3_S_2_PtCl_2_]: 687.1 [M–Cl]^+^, 745.0 [M + Na]^+^, 765.1 [M–Cl + DMSO]^+^; found, 687.1 [M–Cl]^+^, 745.0 [M + Na]^+^, 765.1 [M–Cl + DMSO]^+^. EA calcd for C_24_H_28_N_2_O_3_S_2_PtCl_2_ (%): C, 38.45; H, 4.17; N, 3.74; S, 8.55 [M + 3/2H_2_O]. Found: C, 38.39; H, 3.82; N, 3.50; S, 8.27 [M + 3/2H_2_O]. UV (DMSO) λ_max_, nm (ε, M^–1^ cm^–1^): 303 (1.58 × 10^4^), 436 (5.22 × 10^4^), 450 (5.29 × 10^4^).

#### Complex **C4**

A solution of K_2_PtCl_4_ (57.2 mg, 0.14 mmol) in water (1.5 mL) was added to a solution of **L4** (69.6 mg, 0.13 mmol) in THF (1.5 mL). The solution was stirred at room temperature under argon for 20 h. After this time, an orange solid precipitated. After filtration, the solid was washed with THF and water and dried to afford **C4** as an orange powder (102.9 mg, 99%). mp >200 °C (from THF/H_2_O). IR (ATR): 3299, 2930, 1762, 1701, 1578, 1529, 1503, 1459, 1416, 1336, 1266, 1241, 1184, 1120, 1041, 997, 951, 808, 760 cm^–1^. ^1^H NMR (400 MHz, DMF-*d*_7_): δ 8.74–8.57 (m), 7.52–7.14 (m), 7.13–6.96 (m), 6.95–6.79 (m), 5.07–4.80 (m), 4.07–3.85 (m), 3.81–3.72 (m), 3.69–3.60 (m), 3.37–2.83 (m), 2.70–2.05 (m), 1.88–1.74 (m). HRMS (ESI^+^): calcd for [C_27_H_34_N_2_O_5_S_2_PtCl_2_]: 797.0994 [M + H]^+^, 819.0813 [M + Na]^+^, 835.0551 [M + K]^+^; found, 797.0978 [M + H]^+^, 819.0798 [M + Na]^+^, 835.0551 [M + K]^+^. EA calcd for C_27_H_34_N_2_O_5_S_2_PtCl_2_ (%): C, 39.81; H, 4.45; N, 3.44; S, 7.87 [M + H_2_O]. Found: C, 39.42, H, 4.15, N, 3.15, S, 7.49 [M + H_2_O]. UV (DMF) λ_max_, nm (ε, M^–1^ cm^–1^): 307 (1.31 × 10^4^), 429 (6.40 × 10^4^), 448 (6.18 × 10^4^).

#### Complex **C5**

A solution of K_2_PtCl_4_ (54.8 mg, 0.13 mmol) in H_2_O (2 mL) was added to a brown suspension of **L5** (63.8 mg, 0.13 mmol) in THF (2 mL). The solution was stirred at room temperature under argon for 16 h. After this time, a brown solid precipitated. After filtration, the solid was washed with H_2_O, MeOH, and THF and dried to afford **C5** (80.5 mg, 0.11, 85%) as a brown solid. mp >200 °C (from THF/H_2_O). IR (ATR): 3317, 1767, 1699, 1585, 1526, 1462, 1426, 1311, 1187, 1139, 1032, 998, 958, 819 cm^–1^. ^1^H NMR (400 MHz, DMSO-*d*_6_): δ 8.98–8.81 (m), 8.69–8.35 (m), 7.11–6.75 (m), 6.65–6.51 (m), 4.89–4.64 (m), 3.93–3.57 (m), 3.44–3.07 (m), 2.65–1.62 (m). MS (ESI^+^): calcd for [C_24_H_28_N_2_O_5_S_2_PtCl_2_]: 719.1 [M–Cl]^+^, 755.1 [M + H]^+^, 777.0 [M + Na]^+^, 797.1 [M–Cl + DMSO]^+^; found, 719.1 [M–Cl]^+^, 755.0 [M + H]^+^, 777.0 [M + Na]^+^, 797.1 [M–Cl + DMSO]^+^. EA calcd for C_24_H_28_N_2_O_5_S_2_PtCl_2_ (%): C, 37.31; H, 3.91; N, 3.63; S, 8.30 [M + H_2_O]. Found: C, 37.14, H, 3.88, N, 3.31, S, 7.67 [M + H_2_O]. UV (DMSO) λ_max_, nm (ε, M^–1^ cm^–1^): 306 (1.28 × 10^4^), 439 (5.62 × 10^4^), 455 (5.78 × 10^4^).

#### Complex **C6**

A solution of K_2_PtCl_4_ (67.0 mg, 0.16 mmol) in H_2_O (2 mL) was added to a solution of **L6** (96.3 mg, 0.16 mmol) in THF (2 mL). The resulting mixture was stirred at room temperature under argon for 16 h. After this time, a sticky orange solid precipitated, which was filtrated and washed with H_2_O, MeOH, and THF and dried. The sticky solid was digested with Et_2_O to give pure **C6** (111.3 mg, 0.13 mmol, 81%) as an orange powder. mp 135–139 °C (from THF/H_2_O). IR (ATR): 2869, 1762, 1704, 1583, 1533, 1509, 1460, 1424, 1371, 1298, 1236, 1176, 1138, 1093, 1057, 954, 849, 817 cm^–1^. ^1^H NMR (400 MHz, DMSO-*d*_6_): δ 8.65–8.51 (m), 7.66–7.51 (m), 7.25–7.08 (m), 7.02–6.80 (m), 4.93–4.66 (m), 4.23–4.04 (m), 3.88–3.68 (m), 3.65–3.48 (m), 3.47–3.40 (m), 3.26–3.20 (m), 2.92–2.75 (m), 2.61–2.34 (m), 2.18–2.03 (m), 2.01–1.96 (m), 1.95–1.78 (m). MS (ESI^+^): calcd for [C_31_H_42_N_2_O_6_S_2_PtCl_2_]: 869.2 [M + H]^+^, 891.1 [M + Na]^+^; found, 869.2 [M + H]^+^, 891.1 [M + Na]^+^. EA calcd for C_31_H_42_N_2_O_6_S_2_PtCl_2_ (%): C, 41.99, H, 5.00, N, 3.16, S, 7.23 [M + H_2_O]. Found: C, 42.07, H, 4.79, N, 3.14, S, 7.15 [M + H_2_O]. UV (DMF) λ_max_, nm (ε, M^–1^ cm^–1^): 304 (1.90 × 10^4^), 429 (7.19 × 10^4^), 446 (6.93 × 10^4^).

#### Complex **C7**

A solution of K_2_PtCl_4_ (109.6 mg, 0.26 mmol) in H_2_O (4 mL) was added to a solution of **L7** (250.4 mg, 0.26 mmol) in THF (4 mL). The solution was stirred at room temperature under argon for 17 h. After this time, 20 mL of brine was added, and the product was extracted with CH_2_Cl_2_ (3 × 20 mL). The combined organic layers were dried over anhydrous Na_2_SO_4_ and filtrated. The product was recrystallized in CH_2_Cl_2_/Et_2_O to give **C7** (207.2 mg, 0.17 mmol, 65%) as a brown solid. mp 92–95 °C (from CH_2_Cl_2_/Et_2_O). IR (ATR): 2874, 1764, 1707, 1590, 1530, 1503, 1459, 1429, 1338, 1248, 1096, 951.92, 851 cm^–1^. ^1^H NMR (400 MHz, CDCl_3_): δ 7.12–6.50 (m), 4.44–4.04 (m), 3.97–3.44 (m), 3.39–3.30 (m), 2.62–2.01 (m), 1.82 (s). MS (ESI^+^): calcd for [C_45_H_70_N_2_O_14_S_2_PtCl_2_]: 1215.3 [M + Na]^+^; found, 1215.3 [M + Na]^+^. EA calcd for C_45_H_70_N_2_O_14_S_2_PtCl_2_ (%): C, 42.64; H, 5.57; N, 2.21; S, 5.06 [M + KCl]. Found: C, 42.73, H, 5.58, N, 2.11, S, 4.82 [M + KCl]. UV (H_2_O) λ_max_, nm (ε, M^–1^ cm^–1^): 221 (3.19 × 10^4^), 237 (2.83 × 10^4^), 312 (1.11 × 10^4^), 432 (3.66 × 10^4^), 462 (2.71 × 10^4^).

#### Complex **C8**

A solution of K_2_PtCl_4_ (235.5 mg, 0.57 mmol) in H_2_O (6 mL) was added to a solution of **L8** (*M*_w_ = 2439, 1.06 g, 0.43 mmol) in H_2_O (7 mL). The solution was stirred at room temperature under argon for 16 h. After this time, brine (20 mL) was added, and the mixture was extracted with CH_2_Cl_2_ (3 × 20 mL). The combined organic extracts were dried over anhydrous Na_2_SO_4_ and filtered, the solvent was removed under reduced pressure, and the residue was recrystallized from CH_2_Cl_2_/Et_2_O to give **C8** (1.10 g, 0.41 mmol, 95%) as a brown solid. mp 50–53 °C (from CH_2_Cl_2_/Et_2_O). IR (ATR): 2878, 1765, 1706, 1596, 1510, 1466, 1342, 1279, 1241, 1145, 1100, 1060, 961, 842 cm^–1^. ^1^H NMR (400 MHz, CDCl_3_): δ 7.76–6.45 (m), 4.99–3.48 (m), 3.46–3.40 (m), 3.36 (s), 2.79–2.00 (m). ICP-OES: 6.7% Pt. UV (H_2_O) λ_max_, nm (ε, M^–1^ cm^–1^): 235 (2.86 × 10^4^), 308 (1.36 × 10^4^), 434 (2.75 × 10^4^), 462 (2.18 × 10^4^).

#### Complex **C20**

A solution of K_2_PtCl_4_ (60.9 mg, 0.15 mmol) in H_2_O (2 mL) was added to a solution of **L20** (98.1 mg, 0.14 mmol) in THF (2 mL). The solution was stirred at room temperature under argon for 17 h. After this time, 20 mL of brine was added, and the product was extracted with CH_2_Cl_2_ (3 × 20 mL). The combined organic layers were dried over anhydrous Na_2_SO_4_ and filtrated. The product was recrystallized from CH_2_Cl_2_/Et_2_O to give pure **C20** (114.0 mg, 0.12 mmol, 86%) as a dark orange solid. mp 190–195 °C (from CH_2_Cl_2_/Et_2_O). IR (ATR): 2929, 2856, 1764, 1702, 1584, 1533, 1506, 1461, 1425, 1253, 1169, 1141, 1104, 955, 910, 851, 819 cm^–1^. ^1^H NMR (360 MHz, CDCl_3_): δ 7.76–7.62 (m), 7.49–7.13 (m), 7.01–6.39 (m), 4.82–4.33 (m), 4.24–3.86 (m), 3.33–1.81 (m), 1.29–0.79 (m). MS (ESI^+^): calcd for [C_40_H_46_N_2_O_3_S_2_SiPtCl_2_]: 925.2 [M–Cl]^+^, 961.2 [M + H]^+^, 983.2 [M + Na]^+^; found, 925.2 [M–Cl]^+^, 961.2 [M + H]^+^, 983.2 [M + Na]^+^. EA calcd for C_40_H_46_N_2_O_3_S_2_SiPtCl_2_ (%): C, 49.99; H, 4.82; N, 2.92; S, 6.67. Found: C, 49.72, H, 4.85, N, 2.72, S, 6.25. UV (CHCl_3_) λ_max_, nm (ε, M^–1^ cm^–1^): 310 (9.15 × 10^3^), 429 (3.83 × 10^4^), 455 (3.02 × 10^4^).

### X-ray Structure Determination

Crystals of **L2** were mounted on a glass fiber and used for the data collection on a Bruker D8 Venture with a photon detector equipped with graphite-monochromated Cu Kα radiation (λ = 1.54178 Å). The data reduction was performed with APEX2^39^ software and corrected for absorption using SADABS.^40^ Crystal structures were solved by direct methods using SIR97 program^41^ and refined by full-matrix least-squares on *F*^2^, including all reflections using anisotropic displacement parameters by means of WINGX crystallographic package.^42^ Generally, anisotropic temperature factors were assigned to all atoms except for hydrogen atoms, which are riding their parent atoms with an isotropic temperature factor arbitrarily chosen as 1.2 times that of the respective parent. Final *R*(*F*), w*R*(*F*^2^), the goodness of fit agreement factors, and details on the data collection and analysis can be found in Table S3. Crystallographic data (excluding structure factors) for the structure reported in this paper have been deposited with the Cambridge Crystallographic Data Centre as supplementary publication Nr. CCDC 2100958 for compound **L2**. Copies of the data can be obtained free of charge on application to the Director, CCDC, 12 Union Road, Cambridge, CB2 1EZ, U.K. (Fax: +44-1223-335033; e-mail: deposit@ccdc.cam.ac.uk).

### Photochemical Characterization

UV–vis absorption measurements were recorded on a HP 8453 spectrophotometer.

Fluorescence emission spectra were recorded using a custom-made spectrofluorometer, where the sample was excited with a cw diode laser (λ_exc_ = 405 nm), and emitted photons were detected using an Andor ICCD camera coupled to a spectrograph. All the emission spectra registered were corrected by the wavelength dependence of the spectra response of the detection system. Samples were prepared in spectroscopic grade solvents and adjusted to a response within the linear range.

Fluorescence quantum yields were determined using the standard method for highly diluted solutions to prevent selfabsorption processes (absorption <0.1 at λ_exc_) and relative to 9,10-bis(phenylethynyl)anthracene in acetonitrile (Φ_fl_ = 0.985).^43^

Continuous irradiation of the solutions of photoactive compounds was performed using a UV lamp (Vilber-Lormat, λ = 365 nm, 4 W) or a blue LED (λ_exc_ ∼ 450 nm,^26^ 3 W).

Photodegradation quantum yields were determined using a reported methodology^44^ and relative to *trans*-azobenzene in acetonitrile (Φ_trans→cis_ = 0.14)^45^ for **L1** and **C1** [excited with a Nd/YAG (Brilliant, Quantel) pulsed laser emitting at 355 nm] and to 1,2-bis(5-chloro-2-methyl-3-thienyl-perfluorocyclopentene) in hexane (Φ_closed→open_ = 0.13)^46^ for **L2–8** and **C2–8** (excited with a diode cw laser with λ_exc_ = 445 nm).

To characterize the photoproducts, 10^–2^ M solutions of **L2** (DMF/H_2_O, 9:1) and **L22** (DMF/EtOH, 6:4) and **C2** (DMF-*d*_7_/D_2_O, 9:1) were irradiated with a blue LED (λ_exc_ ∼ 450 nm,^26^ 10 W) until the total disappearance of the absorption maxima band of the initial species (monitored by UV–vis measurements of aliquots). For **L2** and **L22**, after total photodegradation, H_2_O was added, and the crude products were extracted with CH_2_Cl_2_. The combined extracts were dried over anhydrous Na_2_SO_4_, filtered, and the solvent was removed under a reduced pressure. Products were purified by column chromatography (silica gel, hexane/EtOAc 6:4 to hexane/EtOAc, 3:7). In the case of **C2**, after total photodegradation, the sample was freeze-dried to remove the solvent.

### CD Spectroscopy

CD spectroscopy was performed using a model J-715 spectropolarimeter (JASCO, Gross-Umstadt, Germany) equipped with a computer (J-700 software, JASCO) with 1 cm thick quartz cuvettes. Measurements were carried out at a constant temperature of 25 °C. CD spectra were measured in 10 mM Tris-HCl buffer (pH 7.24). The calf thymus DNA concentration was 50 μM. Different samples with an increasing amount of the Pt complex to study (0, 25, and 50 μM) were incubated at 37 °C for 24 h before spectra were recorded in the range of 200–350 nm. Complexes were added from 10^–3^ M stock solutions in water/DMSO, 98:2, solvent mixtures. Irradiated stocks were prepared by irradiation with a blue LED (λ_exc_ ∼ 450 nm, 3 W) until the total disappearance of the absorption maxima band of the initial species (monitored by UV–vis measurements of aliquots).

### Cell Culture

Human ovarian cancer cells A2780 and human ovarian cancer cells cisplatin-resistant A2780cis were obtained from the European Collection of Authenticated Cell Cultures (ECACC, UK) and were routinely cultured in Dulbecco’s modified Eagle’s medium (Invitrogen) containing 10% heat-inactivated fetal bovine serum (FBS) at 37 °C in a humidified CO_2_ atmosphere. Human adenocarcinoma cells (HeLa) were obtained from American Type Culture Collection (ATCC, Manassas, VA, USA) and were routinely cultured in modified Eagle’s medium alpha (MEM-α, Invitrogen) containing 10% heat-inactivated fetal bovine serum at 37 °C in a humidified CO_2_ atmosphere.

### DNA Isolation

To investigate the Pt content associated with the DNA of A2780 cells, DNA was extracted and purified by using the phenol/chloroform methodology. In brief, 5 × 10^6^ cells were seeded in 150 mm dishes and allowed to attach overnight. Cells were then incubated with the different compounds for 24 h in a 10% serum-containing medium. Following incubation, the supernatant was discarded, and the cells were washed thoroughly 3 times with 5 mL ice-cold PBS. Cells were trypsinized and transferred into a 15 mL tube for centrifugation at 500*g* for 5 min. The obtained pellet was resuspended in lysis buffer (100 mM NaCl, 10 mM Tris-Cl pH 8, 25 mM EDTA pH 8, 0.5% SDS, 0.1 mg/mL proteinase K), and genomic DNA was purified by the phenol/chloroform extraction. The extracted DNA was eluted using 1 mL of the TE buffer (10 mM Tris-Cl pH 9, 0.1 mM EDTA). The DNA concentration and purity of the sample were determined by spectral photometry (NanoDrop 2000c, Thermo Scientific) in triplicate.

### ICP–MS

For quantification of the metal content, 1 mL of the DNA solution was wet-digested by adding a freshly prepared mixture of concentrated HNO_3_ at 105 °C for 1 h. Samples were subsequently dissolved in dilute HNO_3_ (1%, v/v) before being analyzed by ICP–MS. The platinum content was determined by ICP–MS with an Agilent 7900 inductively coupled plasma mass spectrometer.

### Cell Viability Assays

Cell proliferation was evaluated using the PrestoBlue assay. Cells were plated in 96-well sterile plates at a density of 5 × 10^3^ cells per well (100 μL) and allowed to grow for 24 h. Cells were then incubated with various concentrations of the studied compounds and cisplatin (0–200 μM for dose–response curves) for 24 h at 37 °C. The next day, cells were washed three times with a fresh culture medium to remove noninternalized complex excess, either untreated or light-activated, for 15 min and left to grow for an additional 48 h at 37 °C. Following the treatment, 10 μL of the PrestoBlue solution was added to each well, and the plates were incubated for an additional 2 h at 37 °C. Afterward, fluorescence was measured using a Victor3 (PerkinElmer) fluorescence multiwell plate reader with the excitation/emission wavelengths set at 531/572 nm. The cell viability was expressed as percentage values with respect to control cells, and the data are shown as the mean value ± standard error of the mean (SEM) of three independent experiments. Dose–response curves and the corresponding IC_50_ values were obtained by means of nonlinear regression (curve fit), calculated with GraphPad Prism 6.0 software.

### Apoptosis Detection Assay

Assessment of apoptotic, late apoptotic/necrotic, and healthy cells with a fluorescence microscope was performed using the Apoptosis/Necrosis Detection Kit (Abcam 176750). A2780 cells (10^4^ cells/well) were plated on 96-well plates and allowed to adhere overnight. Cells were either untreated or treated with 1.5 μM of complex **C7** and 5 μM of complex **C8** for 24 h. Cells were washed with a fresh medium and either untreated or light-activated for 15 min and incubated for a final time of 72 h. Cells were washed twice with the assay buffer, and then the staining buffer (containing 5 μL of apopxin deep red indicator, 5 μL of nuclear green, and 5 μL of CytoCalcein 450 to each 1 mL of the assay buffer) was added and, finally, incubated for 60 min at room temperature. Afterward, the cells were washed three times with the assay buffer, and a total of 10 random images were taken using the Cy5 (Ex/Em = 630/660 nm), FITC (Ex/Em = 490/520 nm), and violet (Ex/Em = 405/450 nm) channels of a fluorescent microscope at a 20× objective. Cells from images were counted, and the numbers of each cellular state were recorded.

### ROS Production Assay

Measurement of ROS was achieved using the DCFDA reagent ROS detection assay. A2780 cells were plated in a 96-well plate at 2 × 10^4^ cells/well in a medium supplemented with 10% FBS and allowed to adhere overnight. The next day cells were incubated with DCFDA (100 μL/well of a 25 μM solution) for 30 min in the dark. Cells were then washed, either untreated or treated, with cisplatin or complexes **C7** and **C8** at their IC_50_ and incubated for an additional 4 h. After this time, cells were washed, fresh medium was added, and light-activated for 15 min or maintained in the dark to finally be incubated for a 48 h extra time. The experiments were run in triplicate. H_2_O_2_ was used as a positive control at 100 μM. The fluorescence of each well was measured in a Microplate Reader Victor3 (PerkinElmer) at 535 nm after an excitation at 485 nm.

## Supporting Information Available

Synthesis of ligand **L6** from **9**, Synthesis of complex **C20**, Gaussview representations of all calculated conformations, UV–vis absorption spectra of ligands **L1–8** and complexes **C1–8** in solvent mixtures with water, fluorescence quantum yields of ligands **L2–8** and complexes **C2–8**, photochemical properties of ligands **L1–8** and complexes **C1–8**. UV–vis spectra monitoring irradiation of ligand **L22** with 450 nm LED in DMF, MS spectrum (positive mode) of the ligand **L22** photoproduct, IR spectrum of the ligand **L22** photoproduct, ^1^H NMR spectrum of the major photoproduct of the ligand **L22**, ^13^C NMR spectrum of the major photoproduct of the ligand **L22**, ^1^H NMR spectrum and the NOE experiment for the major photoproduct of the ligand **L22**, HRMS of the irradiation crude for complex **C2**: peak [721.1 Da (+H^+^)] and its assigned structure, CD spectra of ct-DNA (50 μM) and ct-DNA incubated with (a) **C7**, (b) **C8**, (c) **C7′**, and (d) **C8′** at different molar ratios (ri), NMR spectra of synthesized compounds, MS spectra of **C1–7** and **C20**, Micrographs of complex-treated (**C1–8**) A2780 cells with a 10% FBS-containing cell culture medium, crystal data for **L2**, CIF file for compound **L2**, final structures obtained from the DFT calculations in the PDB format (PDF)

## Accession Codes

CCDC 2100958 contains the supplementary crystallographic data for this paper. These data can be obtained free of charge via www.ccdc.cam.ac.uk/data_request/cif, or by emailing data_request@ccdc.cam.ac.uk, or by contacting The Cambridge Crystallographic Data Centre, 12 Union Road, Cambridge CB2 1EZ, UK; fax: +44 1223 336033.

## References

[ref1] Chemistry of the Platinum Group Metals: Recent Developments; Hartley F. R., Ed.; Elsevier, 1991. ISBN-13: 978-0444881892. ISBN-10: 0444881891.

[ref2] aKnedel T.-O.; Buss S.; Maisuls I.; Daniliuc C. G.; Schlüsener C.; Brandt P.; Weingart O.; Vollrath A.; Janiak C.; Strassert C. A. Encapsulation of Phosphorescent Pt(II) Complexes in Zn-Based Metal-Organic Frameworks toward Oxygen-Sensing Porous Materials. Inorg. Chem. 2020, 59, 7252–7264. 10.1021/acs.inorgchem.0c00678. 32379464

[ref3] aNkabyo H. A.; Bosman G. W.; Luckay R. C.; Koch K. R. New E,Z platinum(II) complexes of asymmetrically di-substituted-acyl(aroyl)thioureas: Synthesis, characterization, photo-induced isomerism. Inorg. Chim. Acta 2020, 508, 119644–119651. 10.1016/j.ica.2020.119644.

[ref4] aJohnstone T. C.; Suntharalingam K.; Lippard S. J. The Next Generation of Platinum Drugs: Targeted Pt(II) Agents, Nanoparticle Delivery, and Pt(IV) Prodrugs. Chem. Rev. 2016, 116, 3436–3486. 10.1021/acs.chemrev.5b00597. 26865551 PMC4792284

[ref5] aShi H.; Imberti C.; Huang H.; Hands-Portman I.; Sadler P. J. Biotinylated photoactive Pt(iv) anticancer complexes. Chem. Commun. 2020, 56, 2320–2323. 10.1039/C9CC07845B. 31990000

[ref6] Shi H.; Imberti C.; Sadler P. J. Diazido Platinum(IV) Complexes for Photoactivated Anticancer Chemotherapy. Inorg. Chem. Front. 2019, 6, 1623–1638. 10.1039/C9QI00288J.

[ref7] Bonnet S. Why Develop Photoactivated Chemotherapy?. Dalton Trans. 2018, 47, 10330–10343. 10.1039/C8DT01585F. 29978870

[ref8] Mügge C.; Liu R.; Görls H.; Gabbiani C.; Michelucci E.; Rüdiger N.; Clement J. H.; Messori L.; Weigand W. Novel Platinum(II) Compounds with O,S Bidentate Ligands: Synthesis, Characterization, Antiproliferative Properties and Biomolecular Interactions. Dalton Trans. 2014, 43, 3072–3086. 10.1039/C3DT52284A. 24169734

[ref9] aShams Abyaneh F. S.; Eslami Moghadam M.; Divsalar A.; Ajloo D.; Hosaini Sadr M. Improving of Anticancer Activity and Solubility of Cisplatin by Methylglycine and Methyl Amine Ligands Against Human Breast Adenocarcinoma Cell Line. Appl. Biochem. Biotechnol. 2018, 186, 271–291. 10.1007/s12010-018-2715-5. 29516403

[ref10] Pizarro A. M.; McQuitty R. J.; Mackay F. S.; Zhao Y.; Woods J. A.; Sadler P. J. Cellular Accumulation, Lipophilicity and Photocytotoxicity of Diazido Platinum(IV) Anticancer Complexes. ChemMedChem 2014, 9, 1169–1175. 10.1002/cmdc.201402066. 24840112

[ref11] Morales K.; Samper K. G.; Peña Q.; Hernando J.; Lorenzo J.; Rodríguez-Diéguez A.; Capdevila M.; Figueredo M.; Palacios Ò.; Bayón P. Squaramide-Based Pt(II) Complexes as Potential Oxygen-Regulated Light-Triggered Photocages. Inorg. Chem. 2018, 57, 15517–15525. 10.1021/acs.inorgchem.8b02854. 30495945

[ref12] aKaur R.; Rani V.; Abbot V.; Kapoor Y.; Konar D.; Kapil Kumar K. Recent Synthetic and Medicinal Perspectives of Pyrroles: An Overview. J. Pharm., Chem. Biol. Sci. 2017, 1, 17–32.

[ref13] **10** was prepared from (3-bromo)(methyl)sulfane following a procedure already described for (3-chloro)(methyl)sulfane: Nenajdenko V. G.; Shevchenko N. E.; Balenkova E. S. Triflic Anhydride-Promoted Cyclizationof Sulfides: A Convenient Synthesis of Fused Sulfur Heterocycles. Synthesis 2003, 2003, 1191–1200. 10.1055/s-2003-39401.

[ref14] aDe Selms R. C.; Fox C. J.; Riordan R. C. Reactions of squaric acid and some derivatives with thionyl chloride/,-dimethylformamide. Tetrahedron Lett. 1970, 11, 781–782. 10.1016/S0040-4039(01)97828-1.

[ref15] Murias M.; Handler N.; Erker T.; Pleban K.; Ecker G.; Saiko P.; Szekeres T.; Jäger W. Resveratrol Analogues as Selective Cyclooxygenase-2 Inhibitors: Synthesis and Structure-Activity Relationship. Bioorg. Med. Chem. 2004, 12, 5571–5578. 10.1016/j.bmc.2004.08.008. 15465334

[ref16] Urbaniak A.; Delgado M.; Kacprzak K.; Chambers T. C. Activity of Resveratrol Triesters against Primary Acute Lymphoblastic Leukemia Cells. Bioorg. Med. Chem. Lett. 2017, 27, 2766–2770. 10.1016/j.bmcl.2017.04.066. 28499732

[ref17] Daou T. J.; Pourroy G.; Greneche J. M.; Bertin A.; Felder-Flesch D.; Begin-Colin S. Water Soluble Dendronized Iron Oxide Nanoparticles. Dalton Trans. 2009, 2009, 4442–4449. 10.1039/b823187g. 19488441

[ref18] Frisch M. J.; Trucks G. W.; Schlegel H. B.; Scuseria G. E.; Robb M. A.; Cheeseman J. R.; Scalmani G.; Barone V.; Petersson G. A.; Nakatsuji H.; Li X.; Caricato M.; Marenich A. V.; Bloino J.; Janesko B. G.; Gomperts R.; Mennucci B.; Hratchian H. P.; Ortiz J. V.; Izmaylov A. F.; Sonnenberg J. L.; Williams-Young D.; Ding F.; Lipparini F.; Egidi F.; Goings J.; Peng B.; Petrone A.; Henderson T.; Ranasinghe D.; Zakrzewski V. G.; Gao J.; Rega N.; Zheng G.; Liang W.; Hada M.; Ehara M.; Toyota K.; Fukuda R.; Hasegawa J.; Ishida M.; Nakajima T.; Honda Y.; Kitao O.; Nakai H.; Vreven T.; Throssell K.; Montgomery J. A. Jr.; Peralta J. E.; Ogliaro F.; Bearpark M. J.; Heyd J. J.; Brothers E. N.; Kudin K. N.; Staroverov V. N.; Keith T. A.; Kobayashi R.; Normand J.; Raghavachari K.; Rendell A. P.; Burant J. C.; Iyengar S. S.; Tomasi J.; Cossi M.; Millam J. M.; Klene M.; Adamo C.; Cammi R.; Ochterski J. W.; Martin R. L.; Morokuma K.; Farkas O.; Foresman J. B.; Fox D. J. Gaussian 16, Revision C.01; Gaussian, Inc.: Wallingford CT, 2016.

[ref19] Hay P. J.; Wadt W. R. Ab initio effective core potentials for molecular calculations. Potentials for K to Au including the outermost core orbitals. J. Chem. Phys. 1985, 82, 299–310. 10.1063/1.448975.

[ref20] Zhao Y.; Truhlar D. G. The M06 Suite of Density Functionals for Main Group Thermochemistry, Thermochemical Kinetics, Noncovalent Interactions, Excited States, and Transition Elements: Two New Functionals and Systematic Testing of Four M06-Class Functionals and 12 Other Functionals. Theor. Chem. Acc. 2008, 120, 215–241. 10.1007/s00214-007-0310-x.

[ref21] Roy D.; Keith T. A.; Millam J. M. GaussView, Version 6.1; Semichem Inc.: Shawnee Mission, KS, 2016.

[ref22] See Supporting Information for Gaussview representations for all calculated conformations (Figure S6, Table S4).

[ref23] See Supporting Information for Gaussview representations for all calculated conformations (Figure S6).

[ref24] Goswami P. P.; Syed A.; Beck C. L.; Albright T. R.; Mahoney K. M.; Unash R.; Smith E. A.; Winter A. H. BODIPY-Derived Photoremovable Protecting Groups Unmasked with Green Light. J. Am. Chem. Soc. 2015, 137, 3783–3786. 10.1021/jacs.5b01297. 25751156

[ref25] For a similar process also described: Zhao D. C.; Allen A. D.; Tidwell T. T. Preparation and Reactivity of Persistent and Stable Silyl-Substituted Bisketenes. J. Am. Chem. Soc. 1993, 115, 10097–10103. 10.1021/ja00075a027.

[ref26] aHarrowven D.; Sun W.; Wilson D. Steric Buttressing Changes Torquospecificity in Thermal Cyclobutenone Rearrangements, Providing New Opportunities for 5H-Furanone Synthesis. Synthesis 2017, 49, 3091–3106. 10.1055/s-0036-1588850.

[ref27] For micrographs of A2780 cells treated with complexes (**C1–8**) see Figure S12.

[ref28] bSrivastava P.; Singh K.; Verma M.; Sivakumar S.; Patra A. K. Photoactive Platinum(II) Complexes of Nonsteroidal Anti-Inflammatory Drug Naproxen: Interaction with Biological Targets, Antioxidant Activity and Cytotoxicity. Eur. J. Med. Chem. 2018, 144, 243–254. 10.1016/j.ejmech.2017.12.025. 29274491

[ref29] aReedijk J. Why Does Cisplatin Reach Guanine-N7 with Competing S-Donor Ligands Available in the Cell?. Chem. Rev. 1999, 99, 2499–2510. 10.1021/cr980422f. 11749488

[ref30] Perez R. P. Cellular and Molecular Determinants of Cisplatin Resistance. Eur. J. Cancer 1998, 34, 1535–1542. 10.1016/S0959-8049(98)00227-5. 9893624

[ref31] Fuertes M. A.; Alonso C.; Pérez J. M. Biochemical Modulation of Cisplatin Mechanisms of Action: Enhancement of Antitumor Activity and Circumvention of Drug Resistance. Chem. Rev. 2003, 103, 645–662. 10.1021/cr020010d. 12630848

[ref32] Marullo R.; Werner E.; Degtyareva N.; Moore B.; Altavilla G.; Ramalingam S. S.; Doetsch P. W. Cisplatin Induces a Mitochondrial-ROS Response that Contributes to Cytotoxicity Depending on Mitochondrial Redox Status and Bioenergetic Functions. PLoS One 2013, 8, e81162 10.1371/journal.pone.0081162. 24260552 PMC3834214

[ref33] Ott M.; Gogvadze V.; Orrenius S.; Zhivotovsky B. Mitochondria, Oxidative Stress and Cell Death. Apoptosis 2007, 12, 913–922. 10.1007/s10495-007-0756-2. 17453160

[ref34] aJin Z.; Qi S.; Guo X.; Jian Y.; Hou Y.; Li C.; Wang X.; Zhou Q. The modification of a pyrene group makes a Ru(II) complex versatile. Chem. Commun. 2021, 57, 3259–3262. 10.1039/d0cc08400j. 33650582

[ref35] Linnenberg O.; Kondinski A.; Stöcker C.; Monakhov K. Y. The Cu(I)-Catalysed Huisgen 1,3-Dipolar Cycloaddition Route to (Bio-)Organic Functionalisation of Polyoxovanadates. Dalton Trans. 2017, 46, 15636–15640. 10.1039/c7dt03376a. 28960001

[ref36] aElbert K. C.; Jishkariani D.; Wu Y.; Lee J. D.; Donnio B.; Murray C. B. Design, Self-Assembly, and Switchable Wettability in Hydrophobic, Hydrophilic, and Janus Dendritic Ligand-Gold Nanoparticle Hybrid Materials. Chem. Mater. 2017, 29, 8737–8746. 10.1021/acs.chemmater.7b02928.

[ref37] Yu C.-Y.; Godana A. S. Conjugated Polymer Nanoparticles Based on Fluorenes, PEGylated Carbazoles and Diphenylamines. Eur. Polym. J. 2018, 99, 165–171. 10.1016/j.eurpolymj.2017.12.019.

[ref38] Jeong W.; Khazi M. I.; Park D.-H.; Jung Y.-S.; Kim J.-M. Full Color Light Responsive Diarylethene Inks for Reusable Paper. Adv. Funct. Mater. 2016, 26, 5230–5238. 10.1002/adfm.201600032.

[ref39] Bruker Apex2, Bruker AXS Inc.: Madison, Wisconsin, USA, 2004.

[ref40] Sheldrick G. M. SADABS, Program for Empirical Adsorption Correction; Institute for Inorganic Chemistry, University of Gottingen: Germany, 1996.

[ref41] Altomare A.; Burla M. C.; Camalli M.; Cascarano G. L.; Giacovazzo C.; Guagliardi A.; Moliterni A. G. G.; Polidori G.; Spagna R. SIR97: a New Tool for Crystal Structure Determination and Refinement. J. Appl. Crystallogr. 1999, 32, 115–119. 10.1107/S0021889898007717.

[ref42] Sheldrick G. M. SHELX-2014, Program for Crystal Structure Refinement; University of Göttingen: Göttingen, Germany, 2014.

[ref43] Demeter A. First Steps in Photophysics. I. Fluorescence Yield and Radiative Rate Coefficient of 9,10-Bis(phenylethynyl)anthracene in Paraffins. J. Phys. Chem. A 2014, 118, 9985–9993. 10.1021/jp507626h. 25296291

[ref44] Lees A. J. A Photochemical Procedure for Determining Reaction Quantum Efficiencies in Systems with Multicomponent Inner Filter Absorbances. Anal. Chem. 1996, 68, 226–229. 10.1021/ac9507653. 21619240

[ref45] Bassotti E.; Carbone P.; Credi A.; Di Stefano M.; Masiero S.; Negri F.; Orlandi G.; Spada G. P. Effect of Strain on the Photoisomerization and Stability of a Congested Azobenzenophane: A Combined Experimental and Computational Study. J. Phys. Chem. A 2006, 110, 12385–12394. 10.1021/jp062428b. 17091939

[ref46] Higashiguchi K.; Matsuda K.; Asano Y.; Murakami A.; Nakamura S.; Irie M. Photochromism of Dithienylethenes Containing Fluorinated Thiophene Rings. Eur. J. Org. Chem. 2005, 2005, 91–97. 10.1002/ejoc.200400441.

