# Supplementary material for: Synthesis and In Vitro Studies of Photoactivatable Semisquaraine-type Pt(II) Complexes
Source: Inorg Chem. 2022 May 6;61(20):7729–45. doi: 10.1021/acs.inorgchem.1c03957 (PMC9131461; doi:10.1021/acs.inorgchem.1c03957)
Supplement: Supplementary file 1 — ic1c03957_si_001.pdf [file ic1c03957_si_001.pdf]

Supporting Information

Synthesis and In Vitro Studies of Photoactivatable Semisquaraine-type Pt(II) Complexes

Kevin Morales,[†] *Sergi Rodríguez-Calado,*[††] *Jordi Hernando,*[†] *Julia Lorenzo,*[††] *Antonio Rodríguez-Diéguez,* [ς] *Carlos Jaime,*[†] *Pau Nolis,*[†] *Mercè Capdevila,*[†] *Òscar Palacios,*[†] *Marta Figueredo,* *[†] *and Pau Bayón* *[†]

[†] Departament de Química, Universitat Autònoma de Barcelona, 08193 Cerdanyola del Vallès, Spain

[††] Institut de Biotecnologia i Biomedicina (IBB) and Departament de Bioquímica i Biologia Molecular, Campus UAB, 08193 Cerdanyola del Vallès, Spain

[ς]Department of Inorganic Chemistry, Faculty of Science, University of Granada, Av/Severo Ochoa s/n, 18071, Granada, Spain

marta.figueredo@uab.cat

pau.bayon@uab.cat

Table of Contents

Entries:

1. Synthesis of ligand **L6** from **9**. S3

2. Synthesis of complex **C20**. S5

3. NMR spectra of synthesized compounds. S15

4. MS spectra for **C1**-**7** and **C20**. S35

5. References. S78

Tables:

Table S1. Fluorescence quantum yields of ligands **L2**-**8** and complexes **C2**-**8**. S8

Table S2. Photochemical properties for ligands **L1**-**8** and complexes **C1**-**8**. S9

Table S3. Crystal data for **L2**. S40

Table S4. CIF File for compound **L2**. S40

Table S5. Final structures obtained from the DFT calculations in pdb format. S53

Figures:

Figure S1. Gaussview representations for all calculated conformations S6

Figure S2. UV-vis absorption spectra of ligands **L1**-**8** and complexes **C1**-**8** in solvent mixtures with water. S7

Figure S3. UV-vis spectra monitoring irradiation of ligand **L22** with 450 nm LED in DMF. S10

Figure S4. MS spectrum (positive mode) for the ligand **L22** photoproduct. S10

Figure S5. IR spectrum for the ligand **L22** photoproduct. S10

Figure S6. ^{1}H-NMR spectrum (600 MHz, DMF-d_7, 390 K) of major photoproduct of ligand **L22**. S11

Figure S7. ^{13}C-NMR spectrum (600 MHz, DMF-d_7, 390 K) of major photoproduct of ligand **L22**. S11

Figure S8. (a) ^{1}H-NMR spectrum and (b) nOe experiment (600 MHz, DMF-d_7, 250 K) for major photoproduct of ligand **L22**. S12

Figure S9. ^{1}H-^{13}C HMBC experiment (600 MHz, DMF-d_7, 250 K) for **25**, major photoproduct of ligand **L22**. S12

Figure S10. HRMS of the irrariation crude for complex **C2**: peak [721.1 Da (+H^{+})] and its assigned estructure. S13

Figure S11. CD spectra of ct-DNA (50 μM) and ct-DNA incubated with (a) **C7**, (b) **C8**, (c) **C7'** and (d) **C8'** at different molar ratios (ri). S14

Figure S12. Micrographs of complex-treated (**C1**-**8**) A2780 cells with 10% FBS-containing cell culture medium. S39

1. Synthesis of ligand L6 from 9

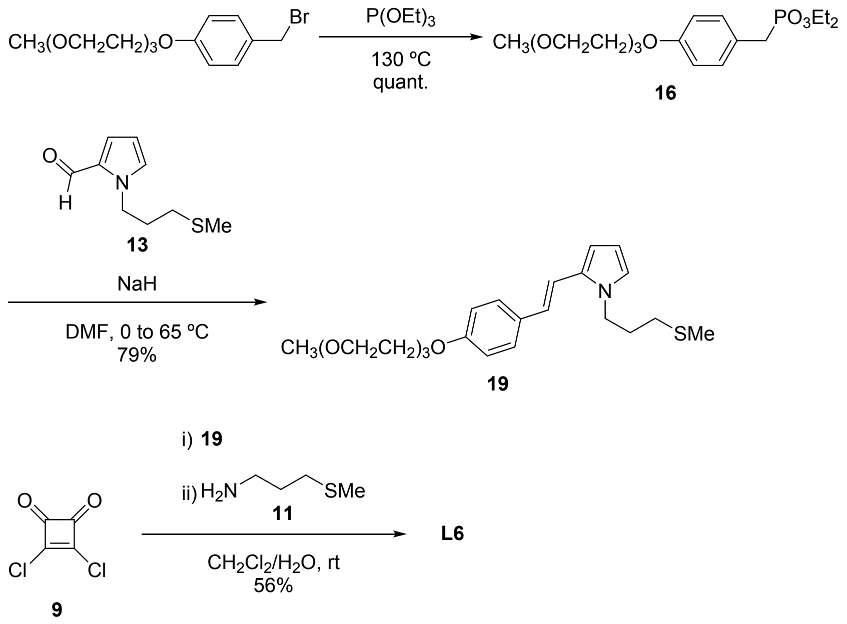

Diethyl (4-{2-[2-(2-methoxyethoxy)ethoxy]ethoxy}benzyl)phosphonate, **16**. A mixture of 1-(bromomethyl)-4-(2-(2-(2-methoxyethoxy)ethoxy)ethoxy)benzene[1] (843 mg, 2.53 mmol) and triethyl phosphite (0.56 mL, 5.01 mmol) were heated at 130°C for 90 min under argon atmosphere. After this time, the mixture was cooled to room temperature, triethyl phosphite excess was removed under reduced pressure and the crude product was purified by column chromatography (silica gel, EtOAc to EtOAc/MeOH, 96:4) to afford **16** (963.6 mg, 2.53 mmol, quantitative yield) as a pale-yellow oil. IR (ATR): 3050, 2981, 2874, 1612, 1511, 1455, 1391, 1352, 1297, 1246, 1197, 1180, 1100, 1052, 1024, 956, 850 cm^{-1}. ^{1}H-NMR (400 MHz, $CDCl_3$): δ 7.16 (dd, *J*= 8.7 Hz, 2.6 Hz, 2H), 6.82 (d, *J*= 8.7 Hz, 2H), 4.09-4.05 (m, 2H), 4.02-3.91 (m, 4H), 3.84-3.79 (m, 2H), 3.72-3.68 (m, 2H), 3.67-3.60 (m, 4H), 3.54-3.49 (m, 2H), 3.34 (s, 3H), 3.05 (d, *J*= 21.1 Hz), 1.20 (t, *J*= 7.1 Hz). ^{13}C-NMR (101 MHz, $CDCl_3$): δ 157.8 (d, *J*= 3.4 Hz), 130.8 (d, *J*= 6.7 Hz), 123.7 (d, *J*= 9.3 Hz), 114.8 (d, *J*= 3.0 Hz), 72.0, 70.9, 70.7, 70.6, 69.8, 67.5, 62.1 (d, *J*= 6.8 Hz), 59.1, 32.8 (d, *J*= 139.1 Hz), 16.4 (d, *J*= 6.0 Hz). HRMS (ESI+): calculated for [$C_{18}H_{31}O_7P$]: 413.1700 $[M+Na]^+$; found 413.1696 $[M+Na]^+$.

(*E*)-2-(4-{2-[2-(2-Methoxyethoxy)ethoxy]ethoxy}styryl)-1-(3-{methylthio}propyl)-1*H*-pyrrole, **19**. A suspension of NaH (60% dispersion in mineral oil, 158 mg, 4.0 mmol) in anhydrous DMF (1.5 mL) was cooled to 0°C under argon atmosphere. Then, a solution of **16** (750 mg, 1.9 mmol) in anhydrous DMF (1.5 mL) was added dropwise. The reaction mixture was stirred at 0°C for 1 h and then a solution of **13** (350 mg, 1.9 mmol) in anhydrous DMF (2 mL) was added and the mixture was heated at 65°C for 30 min. After this time, 10 mL of ice-water were added and the product was extracted with EtOAc (3 x 15 mL). The combined organic extracts were dried over anhydrous Na_2SO_4 and filtered, the solvent was removed under reduced pressure and the residue

was purified by column chromatography (silica gel, hexane/EtOAc, 8:2 to hexane/EtOAc, 4:6) to give **19** (630.6 mg, 1.5 mmol, 79%) as a yellow oil. IR (ATR): 2914, 2872, 1734, 1628, 1604, 1574, 1508, 1474, 1451, 1371, 1353, 1298, 1279, 1242, 1200, 1176, 1106, 1079, 1062, 1044, 951, 849, 815 cm^{-1}. ^{1}H-NMR (400 MHz, CD_3OD): δ 7.42-7.36 (m, 2H), 6.97 (t, *J*= 16.1 Hz, 1H), 6.91-6.86 (m, 2H), 6.80 (d, *J*= 16.1 Hz, 1H), 6.69 (dd, *J*= 2.7 Hz, 1.7 Hz, 1H), 6.40 (ddd, *J*= 3.7 Hz, 1.7 Hz, 0.6 Hz, 1H), 6.06 (ddd, *J*= 3.7 Hz, 2.7 Hz, 0.6 Hz, 1H), 4.12 (t, *J*= 6.8 Hz, 2H), 4.11-4.08 (m, 2H), 3.83-8.79 (m, 2H), 3.70-3.66 (m, 2H), 3.66-3.60 (m, 4H), 3.53-3.49 (m, 2H), 3.34 (s, 3H), 2.41 (t, *J*= 6.8 Hz, 2H), 2.03 (s, 3H), 1.97 (qn, *J*= 6.8 Hz, 2H). ^{13}C-NMR (101 MHz, CD_3OD): δ 159.4, 132.8, 132.4, 128.1, 126.3, 123.4, 116.5, 115.8, 109.2, 106.7, 72.9, 71.7, 71.6, 71.4, 70.9, 68.6, 59.1, 45.8, 31.8, 31.7, 15.3. HRMS (ESI+): calculated for [$C_{23}H_{33}NO_4S$]: 420.2203 $[M+H]^+$; found 420.2204 $[M+H]^+$.

(*E*)-3-(5-{4-[2-(2-{2-Methoxyethoxy}ethoxy)ethoxy]styryl}-1-{3-[methylthio]propyl}-1*H*-pyrrol-2-yl)-4-({3-[methylthio]propyl}amino)cyclobut-3-ene-1,2-dione, **L6**, from **9**: A solution of **19** (211.3 mg, 0.50 mmol) in CH_2Cl_2 (4 mL) was added dropwise to a mixture of a solution of **9** (76.3 mg, 0.51 mmol) in CH_2Cl_2 (9 mL) and H_2O (3 mL). The organic layer turned dark red and the mixture was stirred at room temperature for 2 h. After this time, a solution of **11** (57 µL, 0.51 mmol) and DIPEA (178 µL, 1.0 mmol) in CH_2Cl_2 (4 mL) was added. The resulting mixture was stirred at rt for 1 h. Then, the organic layer was separated, dried over anhydrous Na_2SO_4 and filtered. The solvent was removed under reduced pressure and the residue was crystallized in methanol to give **L6** (171.6 mg, 0.28 mmol, 56%) as a yellow solid.

2. Synthesis of complex C20

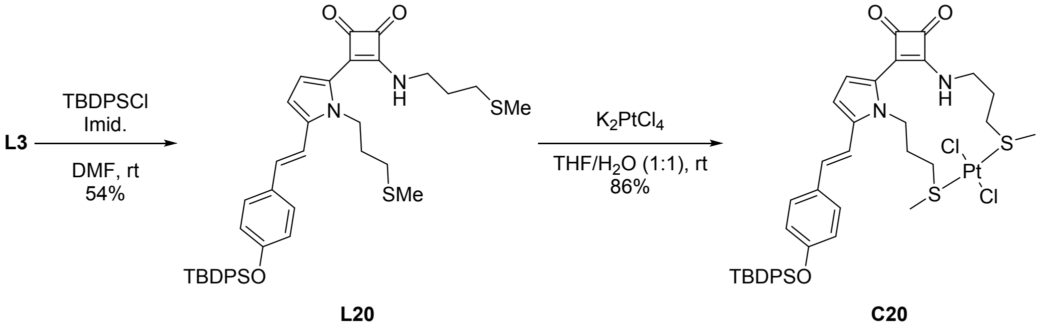

(*E*)-3-(5-{4-[(*tert*-Butyldiphenylsilyl)oxy]styryl}-1-{3-[methylthio]propyl}-1*H*-pyrrol-2-yl)-4-({3-[methylthio]propyl}amino)cyclobut-3-ene-1,2-dione, **L20**. *tert*-Butyldiphenylsilyl chloride (0.35 mL, 1.32 mmol) was added dropwise to a solution of **L3** (274.4 mg, 0.60 mmol) in anhydrous DMF (0.25 mL). The resulting mixture was stirred under argon at room temperature for 18 h. After this time, 5 mL of H_2O were added and the product was extracted with EtOAc (3 x 5 mL). Each organic layer was washed with H_2O (3 x 5 mL). The combined organic extracts were dried over anhydrous Na_2SO_4 and the solvent was removed under reduced pressure. The residue was purified by column chromatography (silica gel, hexane/EtOAc, 1:1) to give pure **L20** (225.2 mg, 0.32 mmol, 53%) as a yellow solid. M.p.: 161-163°C (from CH_2Cl_2). IR (ATR): 3308, 2931, 2857, 1768, 1706, 1601, 1563, 1538, 1507, 1472, 1426, 1390, 1370, 1254, 1174, 1139, 1108, 1057, 954, 912, 853, 819 cm^{-1}. ^{1}H-NMR (400 MHz, $CDCl_3$): δ 7.76-7.70 (m, 4H), 7.47-7.41 (m, 2H), 7.41-7.35 (m, 4H), 7.28-7.23 (m, 2H), 6.95 (d, *J*= 16.0 Hz, 1H), 6.90 (d, *J*= 16.0 Hz, 1H), 6.79-6.74 (m, 2H), 6.62 (d, *J*= 4.3 Hz, 1H), 6.58 (t, *J*= 6.6 Hz, 1H), 6.49 (d, *J*= 4.3 Hz, 1H), 4.72 (t, *J*= 7.1 Hz, 2H), 4.00 (q, *J*= 6.6 Hz, 2H), 2.66 (t, *J*= 6.6 Hz, 2H), 2.48 (t, *J*= 7.1 Hz, 2H), 2.13 (s, 3H), 2.03 (qn, *J*= 6.6 Hz, 2H), 2.02 (s, 3H), 1.97 (qn, *J*= 7.1 Hz, 2H), 1.11 (s, 9H). ^{13}C-NMR (101 MHz, $CDCl_3$): δ 188.5, 185.4, 176.7, 156.6, 156.0, 139.2, 135.6, 132.8, 131.0, 130.1, 130.0, 127.9, 127.7, 125.0, 120.3, 113.7, 112.7, 109.1, 45.1, 45.0, 31.9, 31.3, 31.1, 29.5, 26.6, 19.6, 15.6, 15.6. HRMS (ESI+): calculated for [$C_{40}H_{46}N_2O_3S_2Si$]: 695.2792 $[M+H]^+$, 717.2611 $[M+Na]^+$; found 695.2794 $[M+H\ ]^+$, 717.2617 $[M+Na]^+$. UV ($CHCl_3$) λ_{max}, nm (ε, M^{-1} cm^{-1}): 301 (1.21x10^4), 431 (5.59x10^4), 446 (5.75x10^4).

Complex **C20**. A solution of K_2PtCl_4 (60.9 mg, 0.15 mmol) in H_2O (2 mL) was added to a solution of **L20** (98.1 mg, 0.14 mmol) in THF (2 mL). The resulting solution was stirred at room temperature under argon for 17 h. After this time, 20 mL brine were added and the product was extracted with CH_2Cl_2 (3 x 20 mL). The combined organic extracts were dried over anhydrous Na_2SO_4 and filtrated. The residue was crystallized from CH_2Cl_2/Et_2O to give pure **C20** (114.0 mg, 0.12 mmol, 86%) as a dark orange solid. M.p.: 190-195°C (from CH_2Cl_2/Et_2O). IR (ATR): 2929, 2856, 1764, 1702, 1584, 1533, 1506, 1461, 1425, 1253, 1169, 1141, 1104, 955, 910, 851, 819 cm^{-1}. ^{1}H-NMR (360 MHz, $CDCl_3$): δ 7.76-7.62 (m), 7.49-7.13 (m), 7.01-6.39 (m), 4.82-4.33 (m),

4.24-3.86 (m), 3.33-1.81 (m), 1.29-0.79 (m). MS (ESI+): calculated for [$C_{40}H_{46}N_2O_3S_2SiPtCl_2$]: 925.2 [M-Cl]$^+$, 961.2 [M+H]$^+$, 983.2 [M+Na]$^+$; found, 925.2 [M-Cl]$^+$, 961.2 [M+H]$^+$, 983.2 [M+Na]$^+$. EA calculated for $C_{40}H_{46}N_2O_3S_2SiPtCl_2$ (%): C, 49.99; H, 4.82; N, 2.92; S, 6.67. Found: C, 49.72, H, 4.85, N, 2.72, S, 6.25. UV ($CHCl_3$) λ_{max}, nm (ε, M^{-1} cm^{-1}): 310 ($9.15x10^3$), 429 ($3.83x10^4$), 455 ($3.02x10^4$).

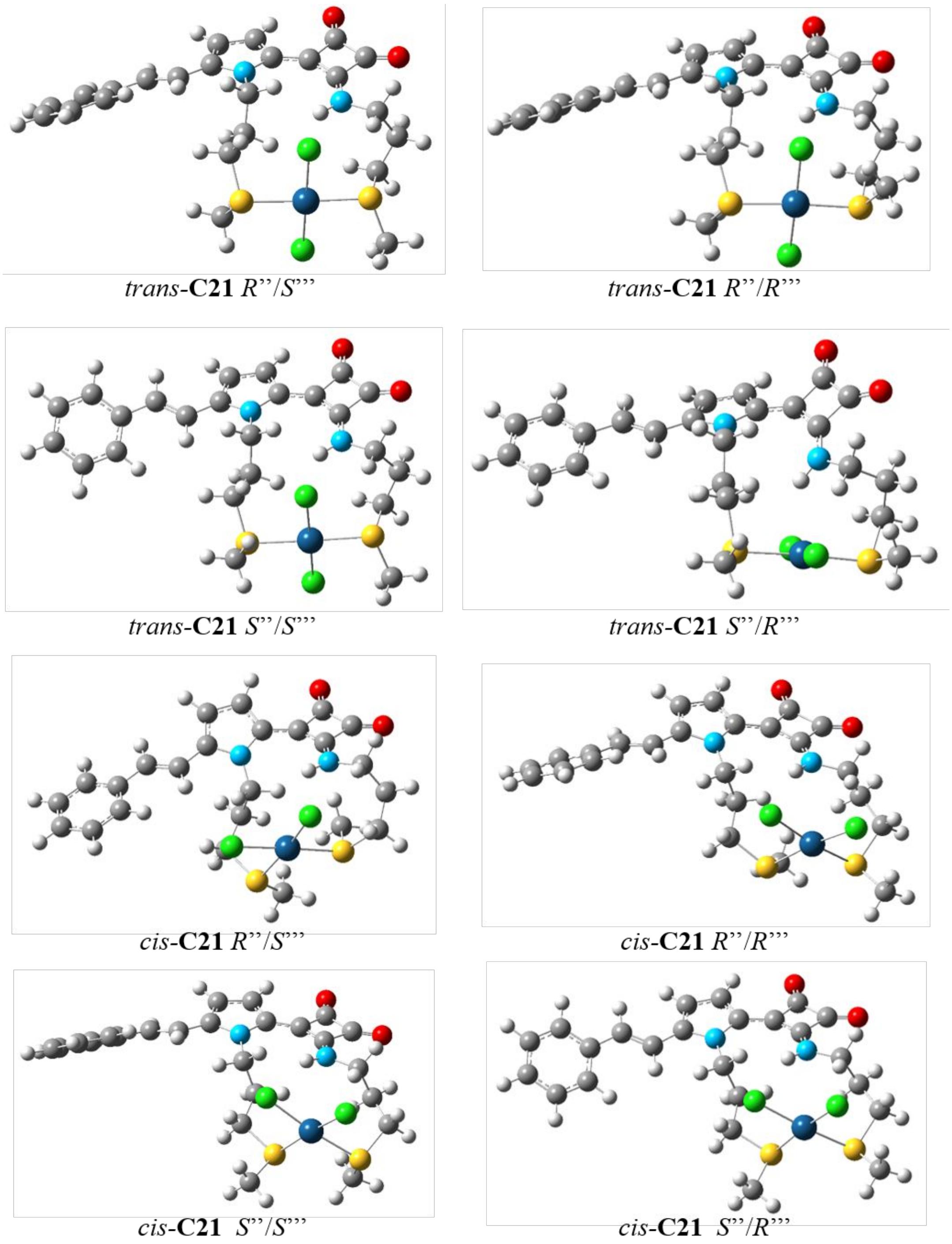

Figure S1. Gaussview representations for all calculated conformations of *trans*- and *cis*-**C21**.

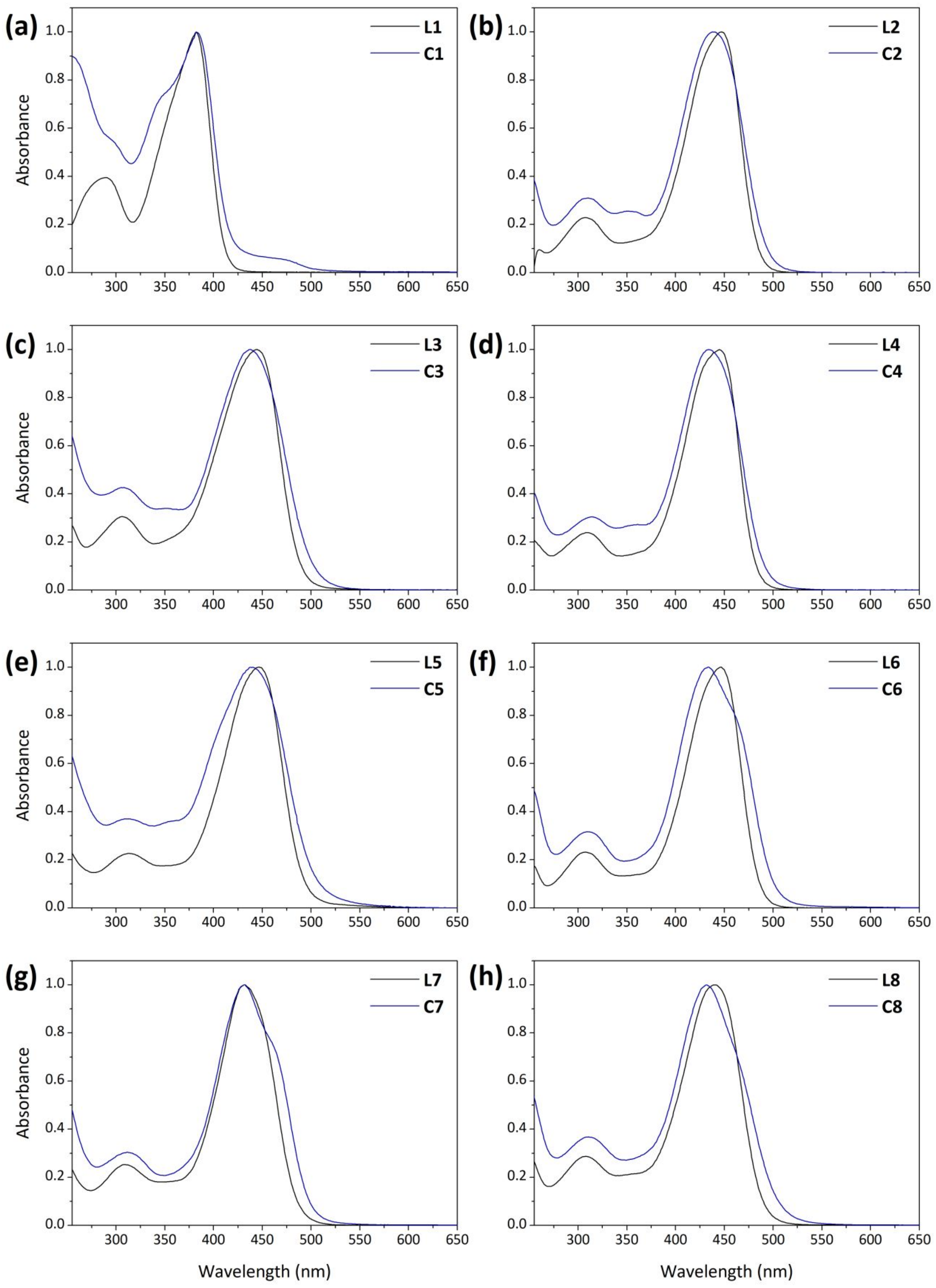

Figure S2. UV-vis absorption spectra of (a) **L1** and **C1**, (b) **L2** and **C2**, (c) **L3** and **C3**, (d) **L4** and **C4**, (e) **L5** and **C5**, (f) **L6** and **C6**, (g) **L7** and **C7**, (h) **L8** and **C8**. Solvent mixtures: water/DMSO, 8:2 (**L1**, **C1**, **C3**, **L5** and **C5**), water/DMF, 6:4 (**L2**), water/DMF, 8:2 (**C2**, **L4**, **C4**, **L6** and **C6**), water/DMSO, 7:3 (**L3**) and water (**L7**, **C7**, **L8** and **C8**).

Table S1. Fluorescence quantum yields of ligands **L2-8** and complexes **C2-8**.

**Entry**	**Compound**	**Solvent**	λ_{max}^{abs} **(nm)**	λ_{max}^{fl} **(nm)**	Φ_{fl}[a]
1	**L2**	MeCN	439	496	0.008
2		$CHCl_3$	446	491	0.05
3		DMF	447	446	0.05
4		H_2O/DMF, 6:4	447	484	0.009
5	**C2**	DMF	429	450	0.02
6		H_2O/DMF, 8:2	438	531	0.002
7	**L3**	H_2O/DMSO, 7:3	445	490	0.005
8	**C3**	H_2O/DMSO, 8:2	438	492	0.003
9	**L4**	H_2O/DMF, 8:2	445	518	0.007
10	**C4**	H_2O/DMF, 8:2	434	531	0.003
11	**L5**	H_2O/DMSO, 8:2	446	511	0.002
12	**C5**	H_2O/DMSO, 8:2	440	529	0.002
13	**L6**	H_2O/DMF, 8:2	446	518	0.006
14	**C6**	H_2O/DMF, 8:2	434	527	0.003
15	**L7**	H_2O	432	511	0.002
16	**C7**	H_2O	432	ND	ND
17	**L8**	H_2O	439	524	0.004
18	**C8**	H_2O	432	ND	ND

[a] Fluorescence quantum yield measured at λ_{exc} = 405 nm. 9,10-Bis(phenylethynyl)anthracene in acetonitrile was used as a reference (Φ_{fl} = 0.985).[2]

Table S2. Photochemical properties for ligands **L1-8** and complexes **C1-8**.

Entry	Compound	Solvent	λ_{max}^{abs} (nm)[a]	$\varepsilon^{\lambda_{max}^{abs}}$ (M^{-1} cm^{-1})	Φ_{ph}[a]	$\Phi_{ph}\,\varepsilon^{\lambda_{max}^{abs}}$
1	**L1**	H_2O/DMSO, 8:2	382	$3.04x10^4$	0.019	578
2	**C1**	H_2O/DMSO, 8:2	382	$1.96x10^4$	0.00003	0.6
3	**L2**	DMF	447	$7.69x10^4$	0.0006	46
4		H_2O/DMF, 6:4	447	$8.10x10^4$	0.00002	2
5	**C2**	DMF	429	$6.14x10^4$	0.0007	43
6		H_2O/DMF, 8:2	438	$4.15x10^4$	0.0003	12
7	**L3**	H_2O/DMSO, 7:3	445	$4.53x10^4$	0.00008	4
8	**C3**	H_2O/DMSO, 8:2	438	$3.05x10^4$	0.0004	12
9	**L4**	H_2O/DMF, 8:2	445	$5.50x10^4$	0.00004	2
10	**C4**	H_2O/DMF, 8:2	434	$3.83x10^4$	0.0004	15
11	**L5**	H_2O/DMSO, 8:2	446	$5.50x10^4$	0.00003	2
12	**C5**	H_2O/DMSO, 8:2	440	$2.96x10^4$	0.0004	12
13	**L6**	H_2O/DMF, 8:2	446	$6.76x10^4$	0.00007	5
14	**C6**	H_2O/DMF, 8:2	434	$6.49x10^4$	0.0003	19
15	**L7**	H_2O	432	$4.06x10^4$	0.00005	2
16	**C7**	H_2O	432	$3.66x10^4$	0.0007	26
17	**L8**	H_2O	439	$3.95x10^4$	0.00004	2
18	**C8**	H_2O	432	$3.20x10^4$	0.0004	13

[a] Photodegradation quantum yield. For ligand **L1** and complex **C1**, it was measured using *trans*-azobenzene as a reference ($\Phi_{trans \rightarrow cis}$ = 0.14 in acetonitrile)[3] and λ_{exc} = 355 nm. For the rest of ligands and complexes, 1,2-bis(5-chloro-2-methyl-3-thienyl)perfluorocyclopentene was used as a reference ($\Phi_{closed \rightarrow open}$ = 0.13 in hexane)[4] at λ_{exc} = 445 nm.

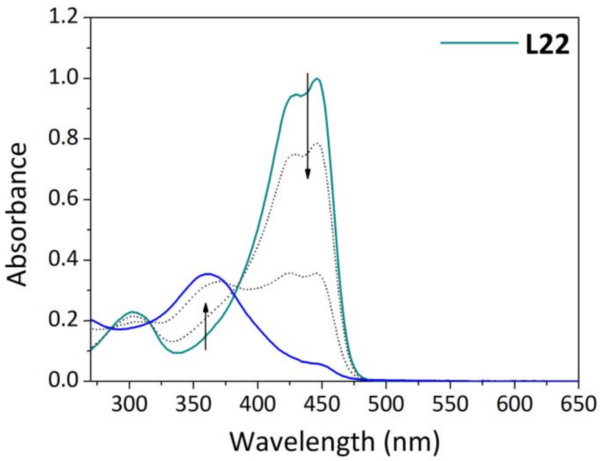

Figure S3. UV-vis spectra monitoring irradiation of ligand **L22** with 450 nm LED in DMF.

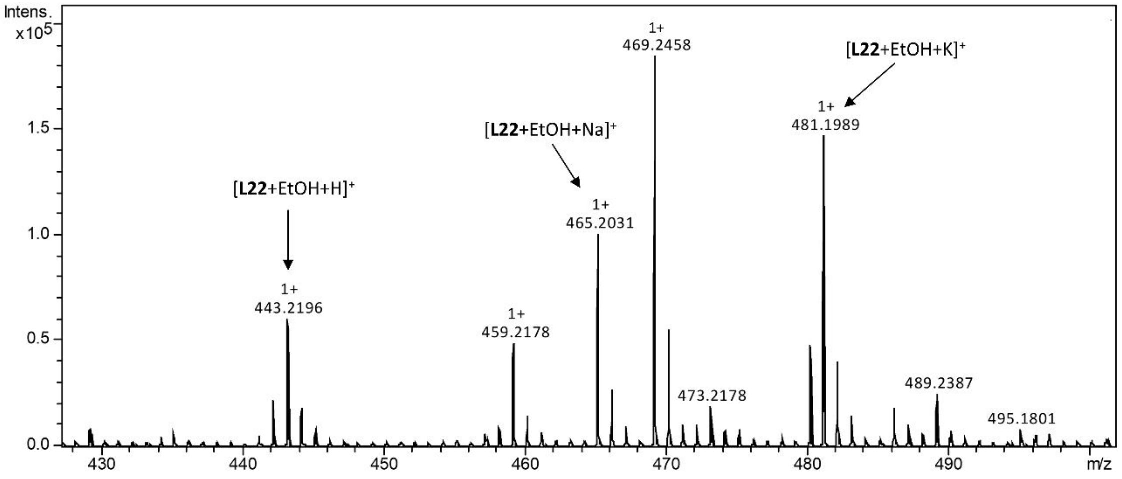

Figure S4. MS spectrum (positive mode) for the photoproduct of ligand **L22**.

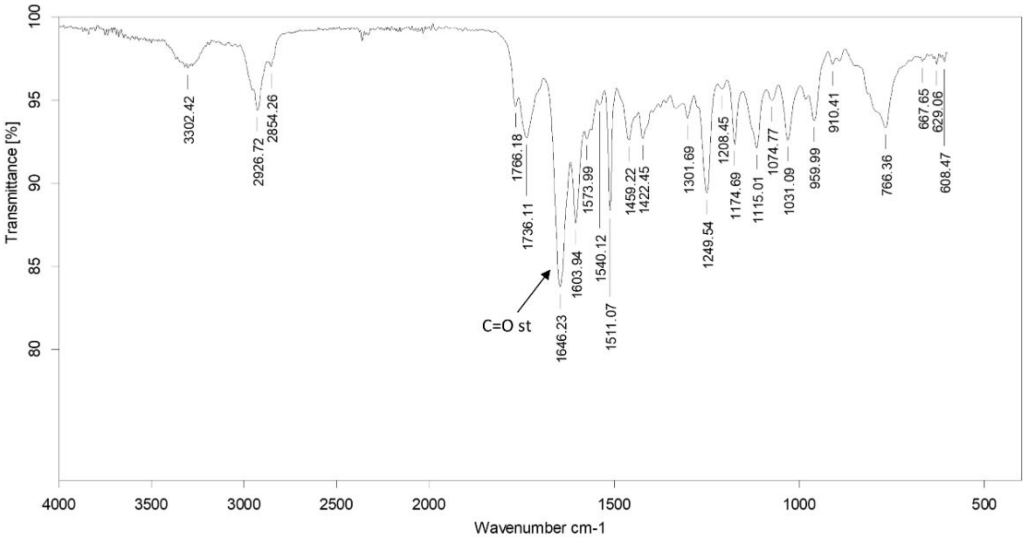

Figure S5. IR spectrum for the photoproduct of ligand **L22**.

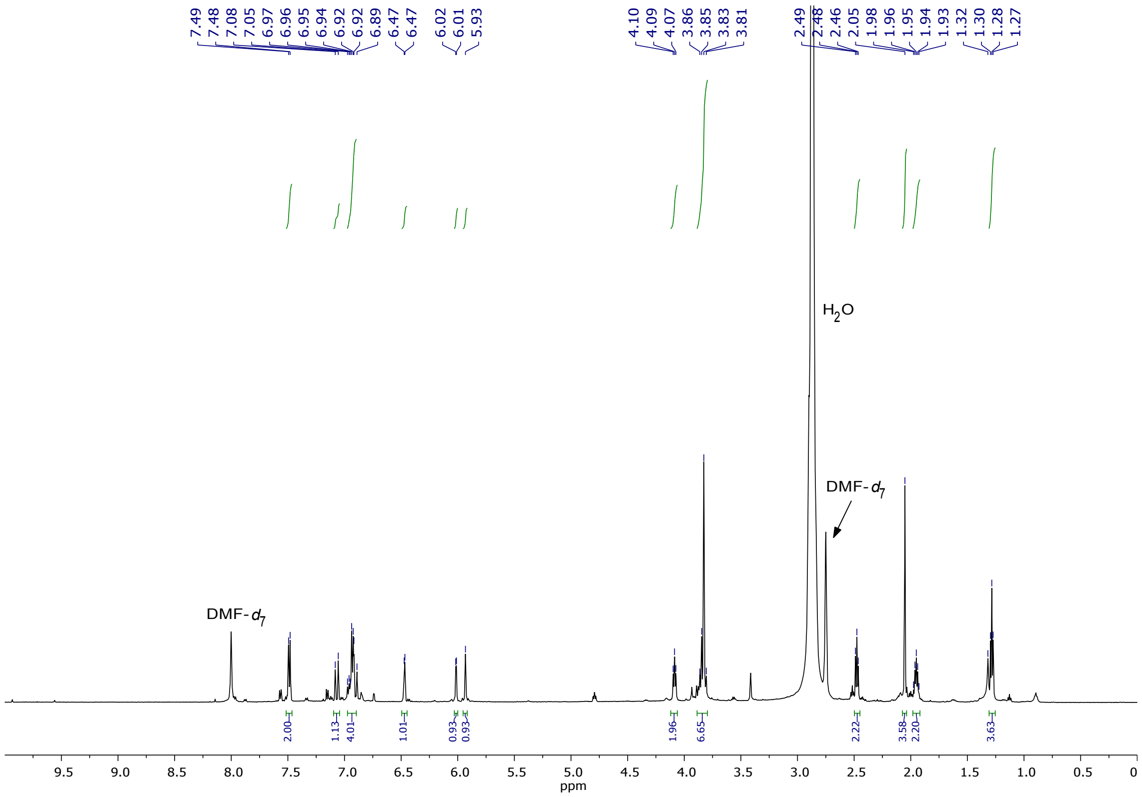

Figure S6. ^{1}H-NMR spectrum (600 MHz, DMF-d_7, 390 K) of **25**, major photoproduct of ligand **L22**.

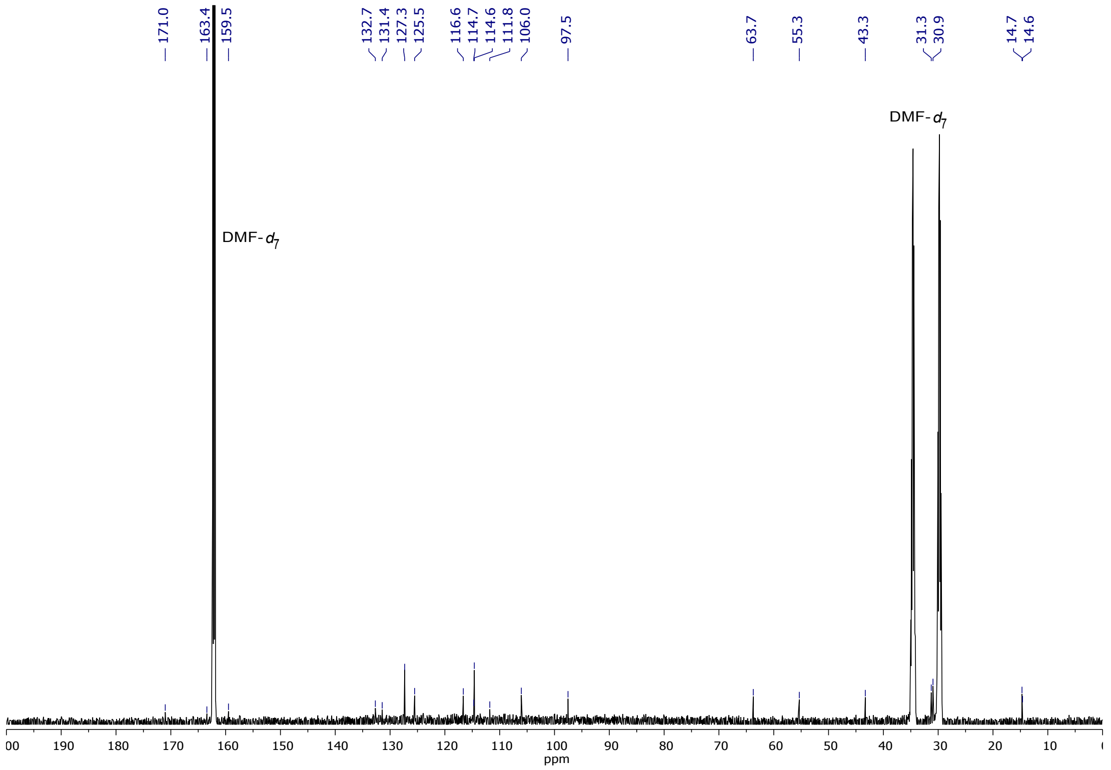

Figure S7. ^{13}C-NMR spectrum (600 MHz, DMF-d_7, 390 K) of **25**, major photoproduct of ligand **L22**.

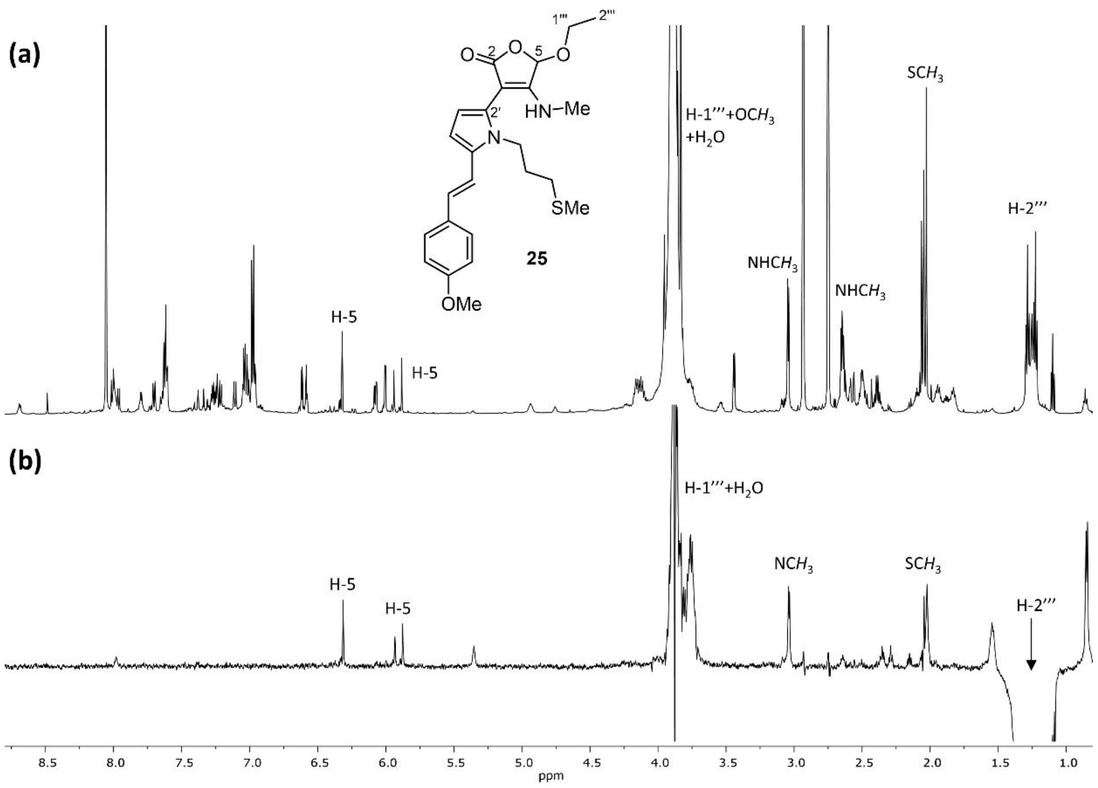

Figure S8. (a) ^{1}H-NMR spectrum and (b) nOe experiment (600 MHz, DMF-d_7, 250 K) for **25**, major photoproduct of ligand **L22**.

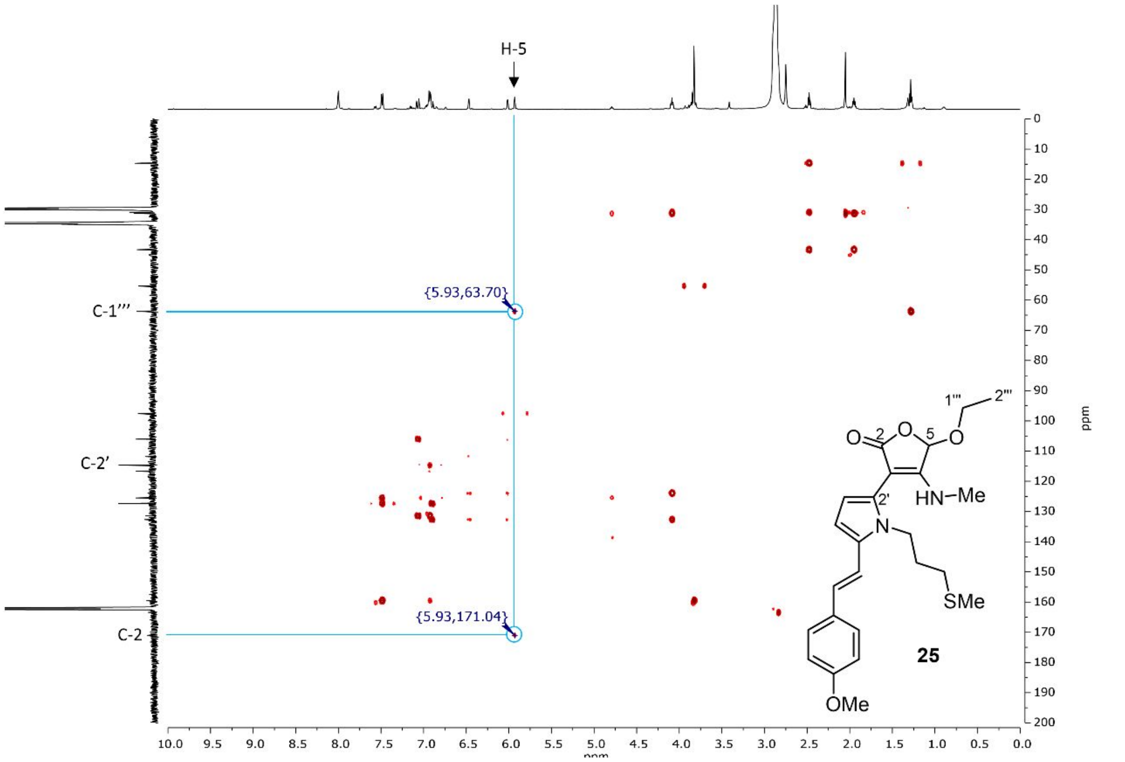

Figure S9. ^{1}H-^{13}C HMBC experiment (600 MHz, DMF-d_7, 250 K) for **25**, major photoproduct of ligand **L22.**

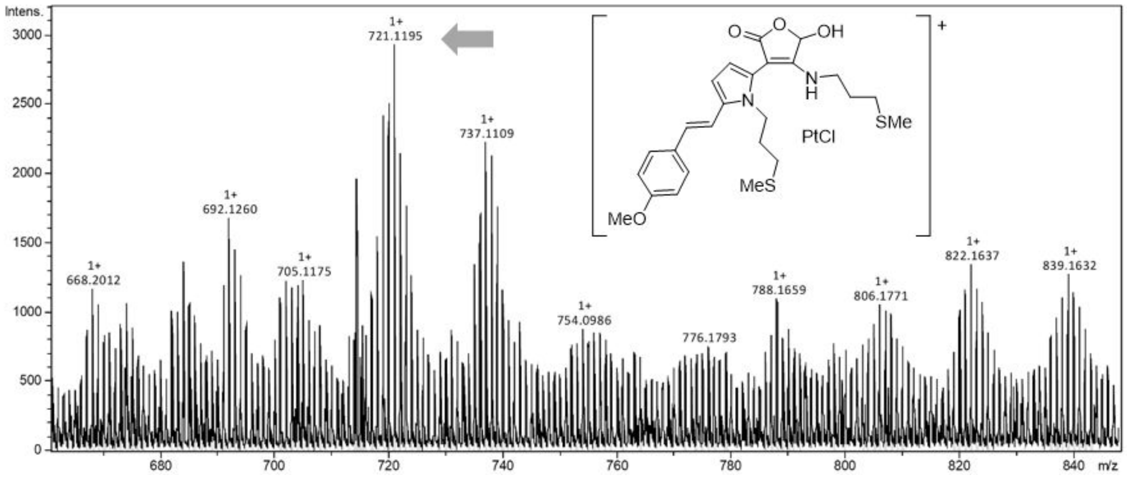

Figure S10. HRMS of the irrariation crude for complex **C2**: peak [721.1 Da [**C2**+H_2O-Cl]$^+$] and its assigned estructure.

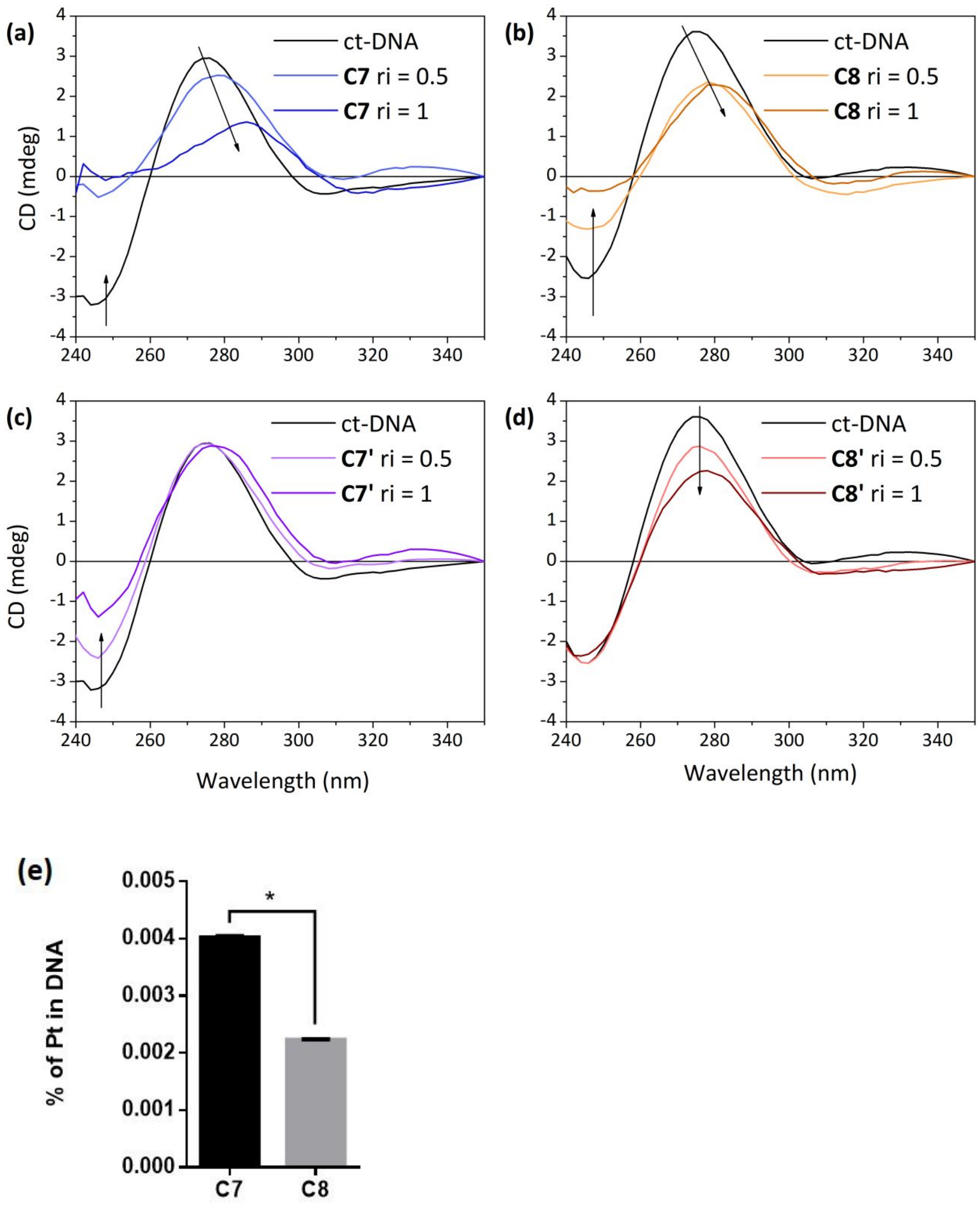

Figure S11. CD spectra of ct-DNA (50 µM) and ct-DNA incubated with (a) C7, (b) C8, (c) C7' and (d) C8' at different molar ratios (ri). (e) Quantitative results of the percentatge of Pt incorporation in DNA from A2780 cells treated with C7 and C8 compounds using their IC50, (20 and 200 µM, respectively). Statistical analysis was performed using a t-test with a N=2.

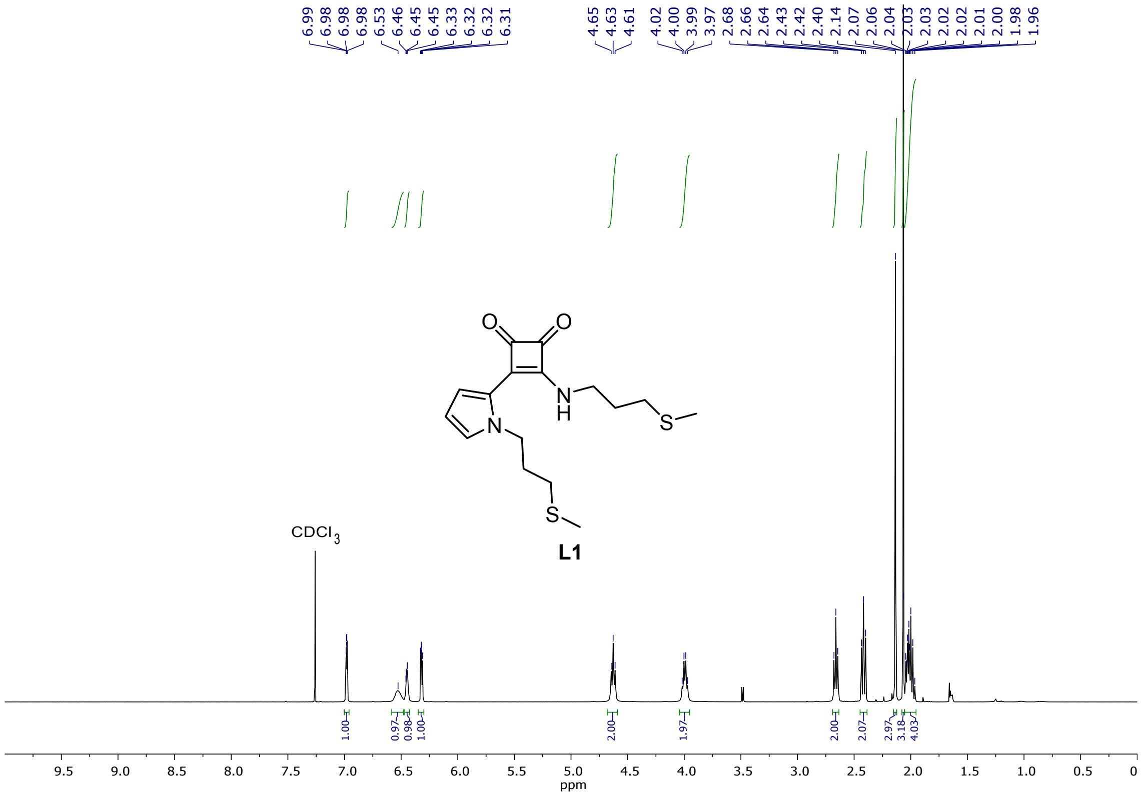

^{1}H-NMR (400 MHz, $CDCl_3$)

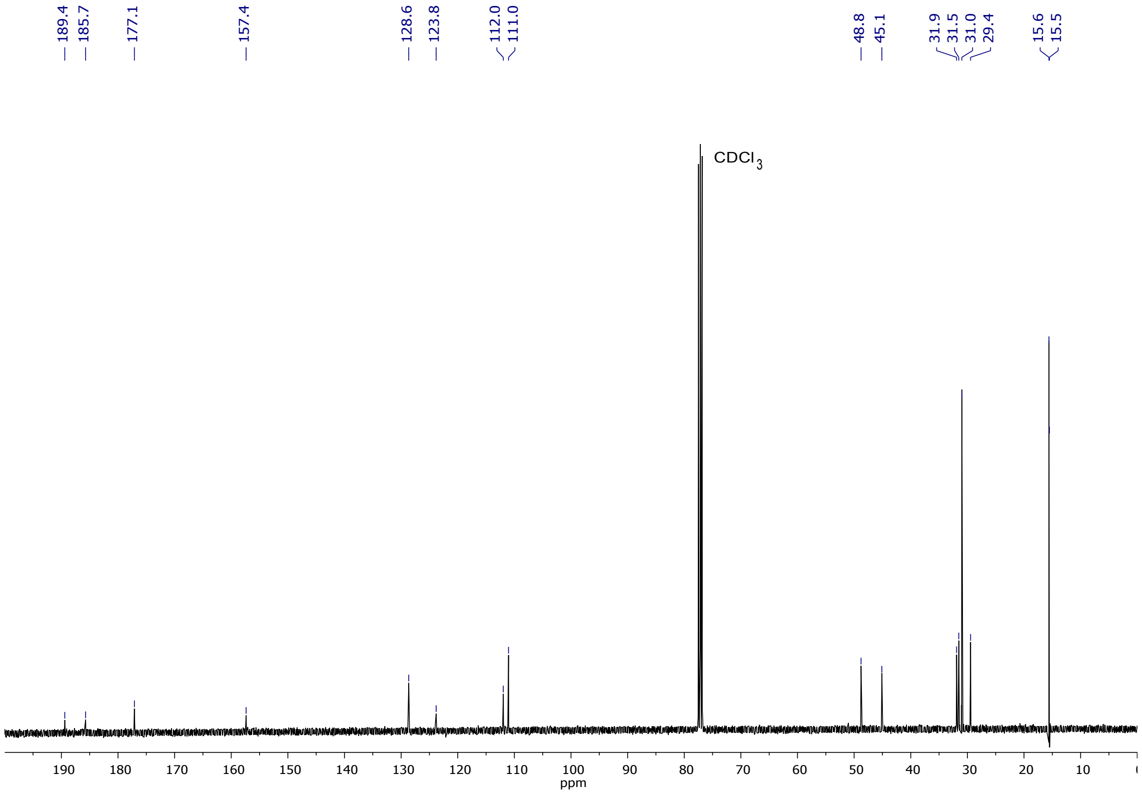

^{13}C-NMR (101 MHz, $CDCl_3$)

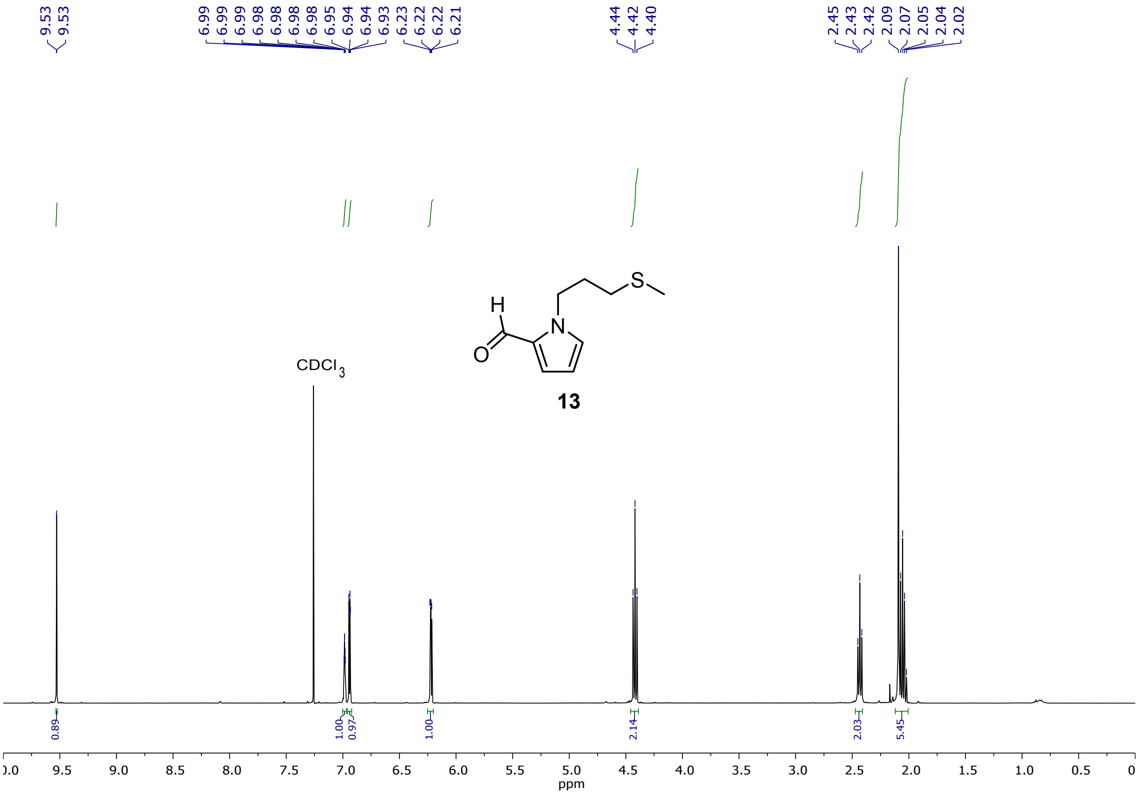

^{1}H-NMR (400 MHz, $CDCl_3$)

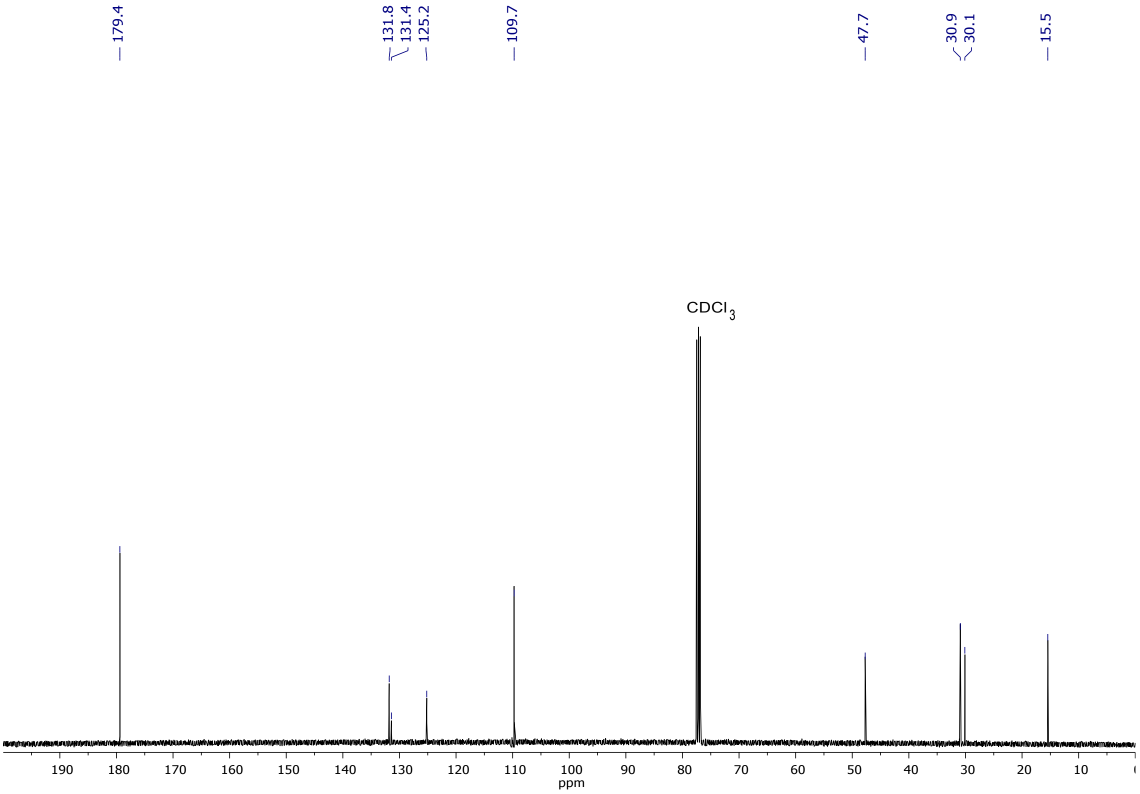

^{13}C-NMR (101 MHz, $CDCl_3$)

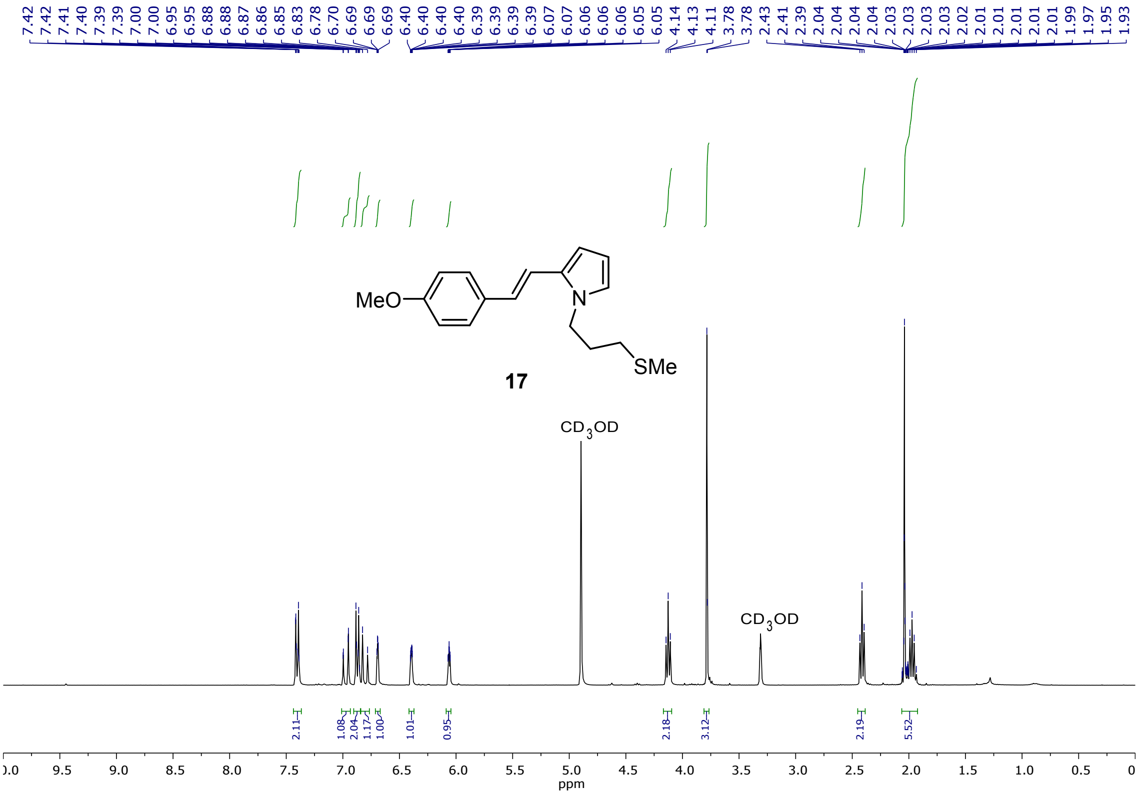

¹H-NMR (360 MHz, CD_3OD)

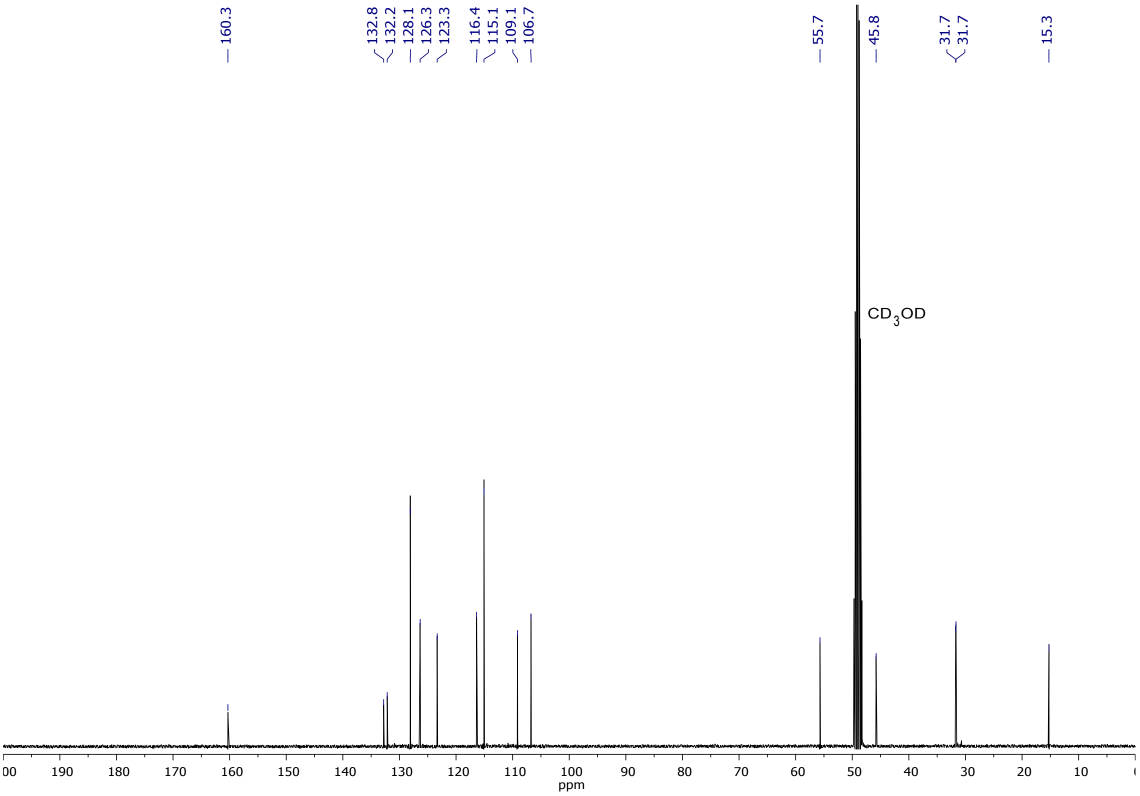

¹³C-NMR (91 MHz, CD_3OD)

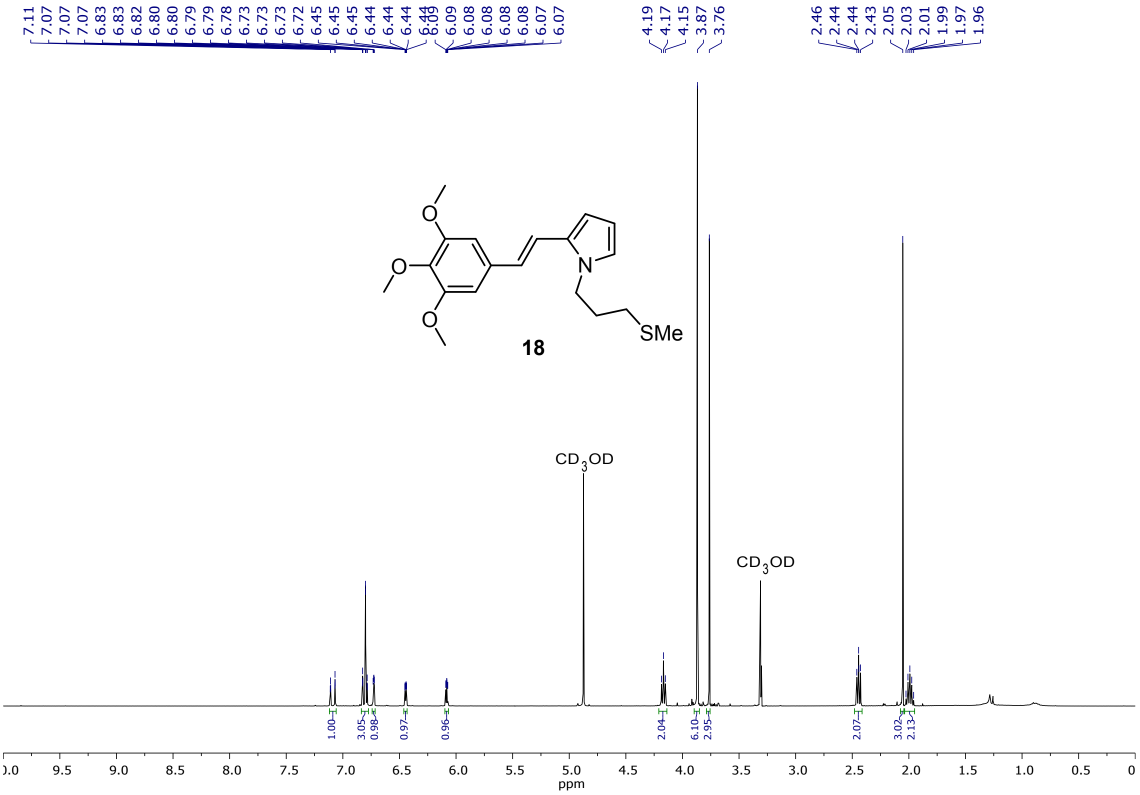

¹H-NMR (400 MHz, CD_3OD)

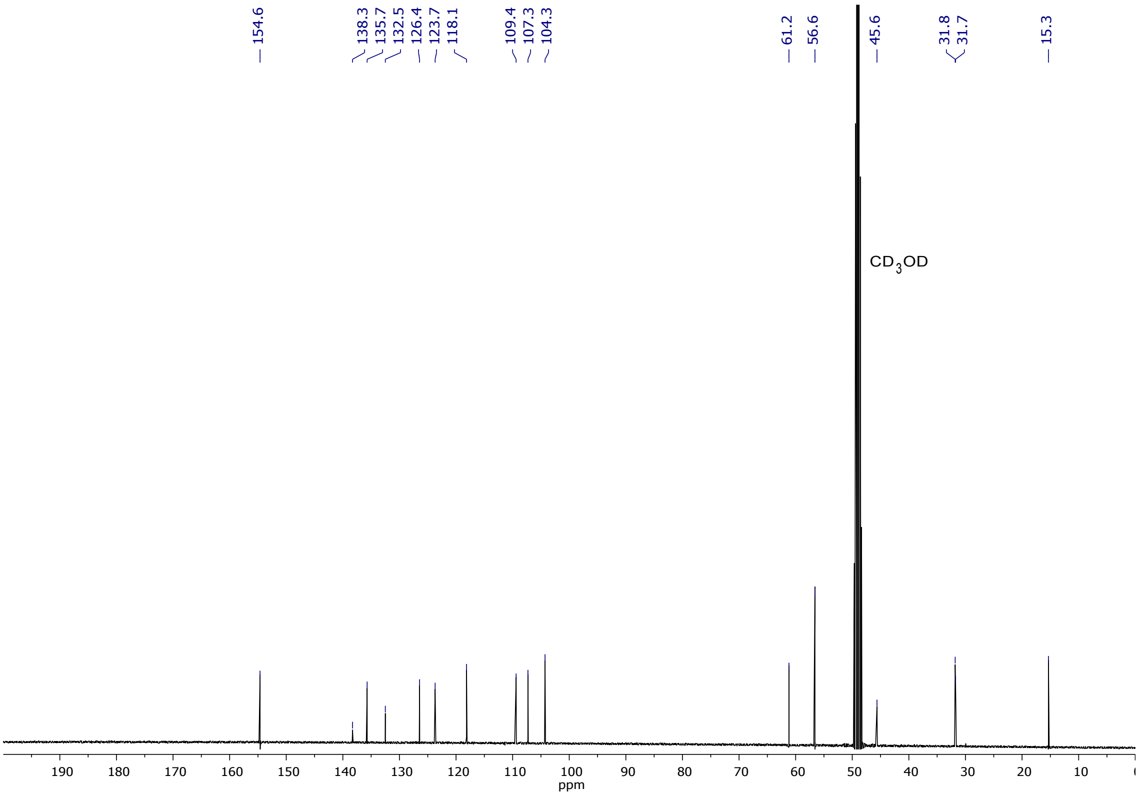

¹³C-NMR (101 MHz, CD_3OD)

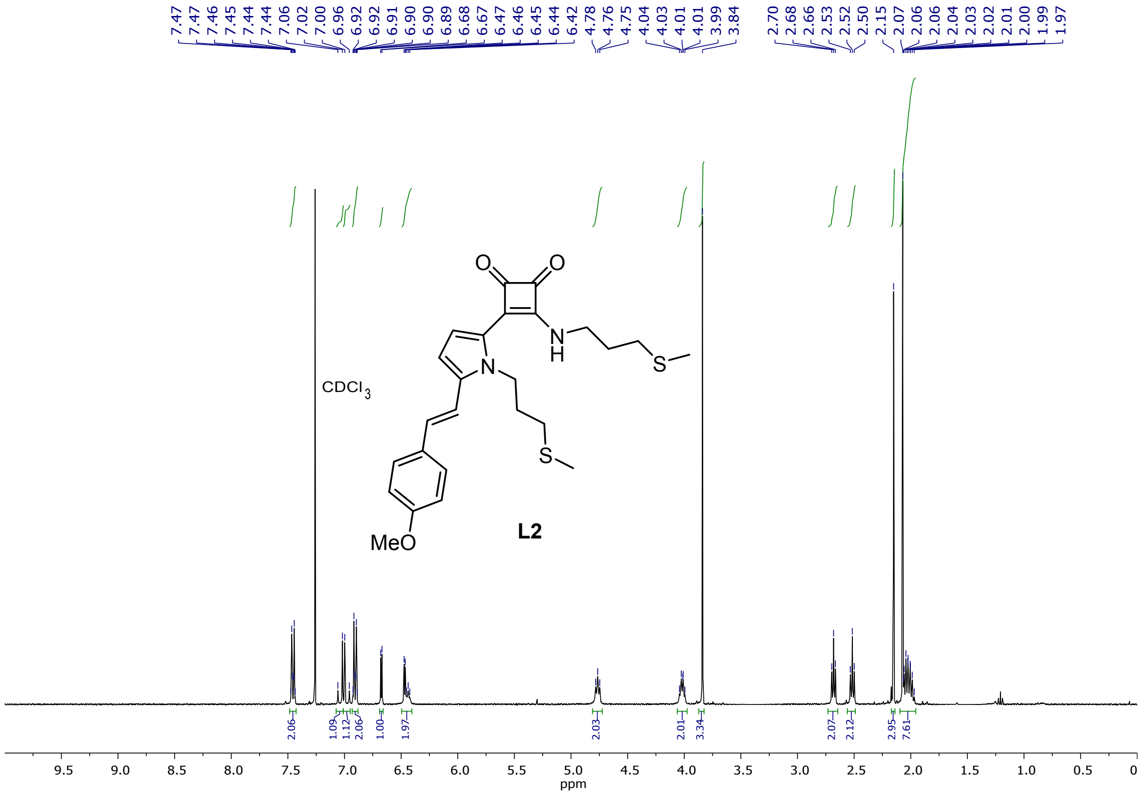

^{1}H-NMR (400 MHz, $CDCl_3$)

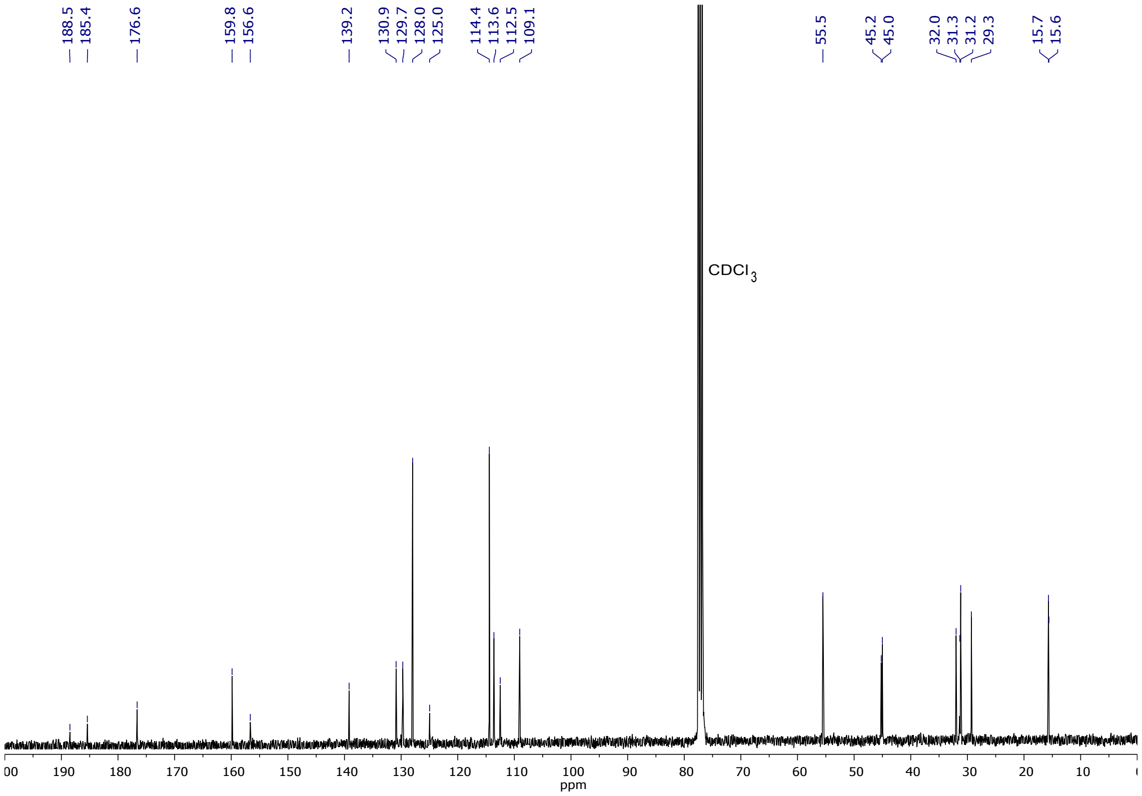

^{13}C-NMR (101 MHz, $CDCl_3$)

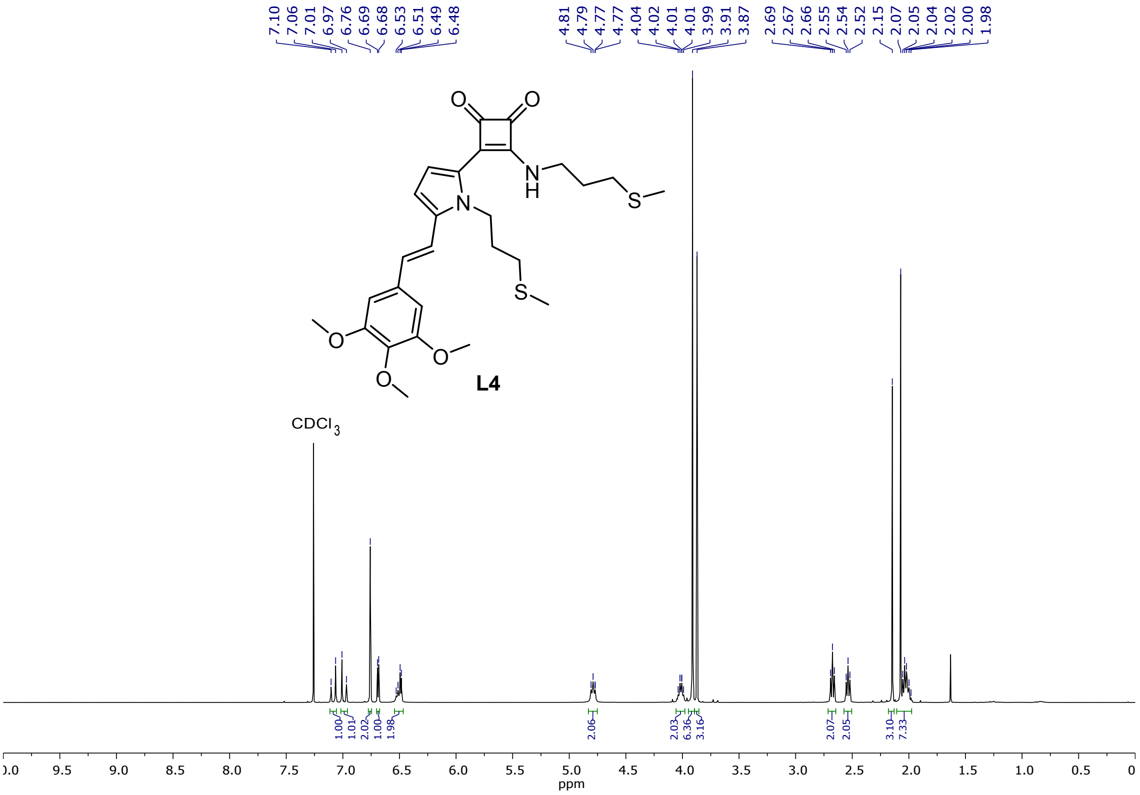

^{1}H-NMR (400 MHz, $CDCl_3$)

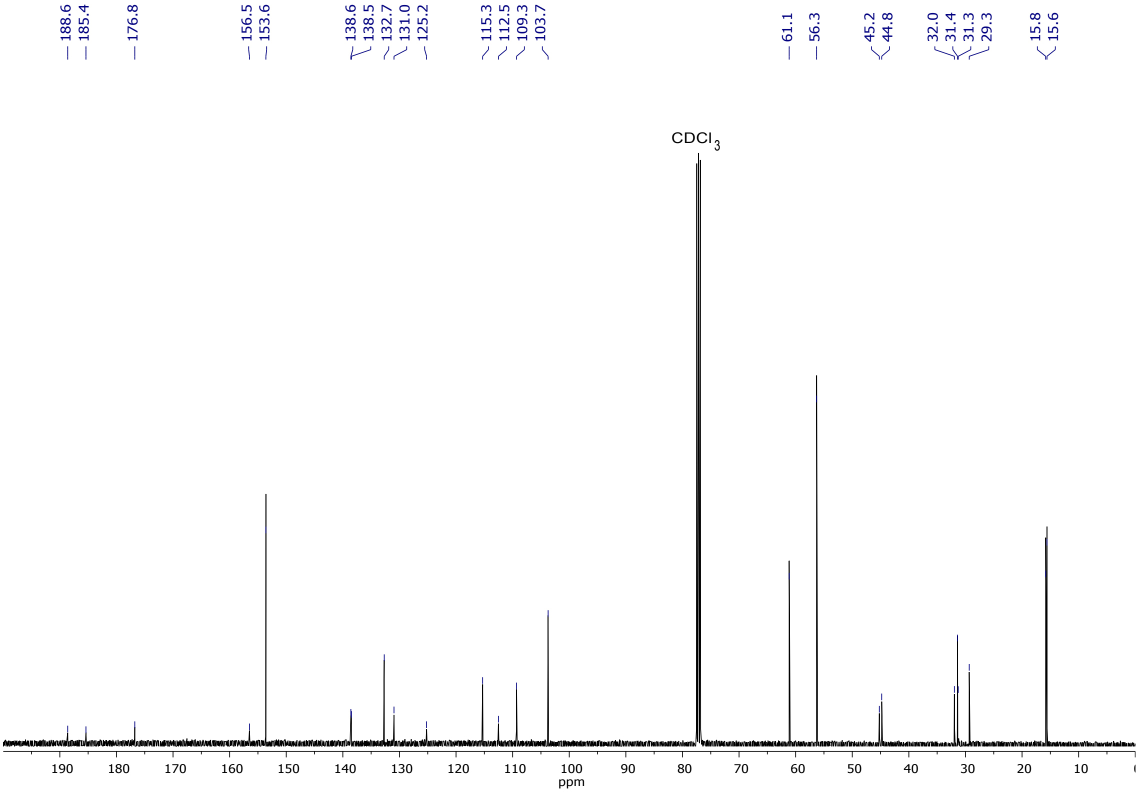

^{13}C-NMR (101 MHz, $CDCl_3$)

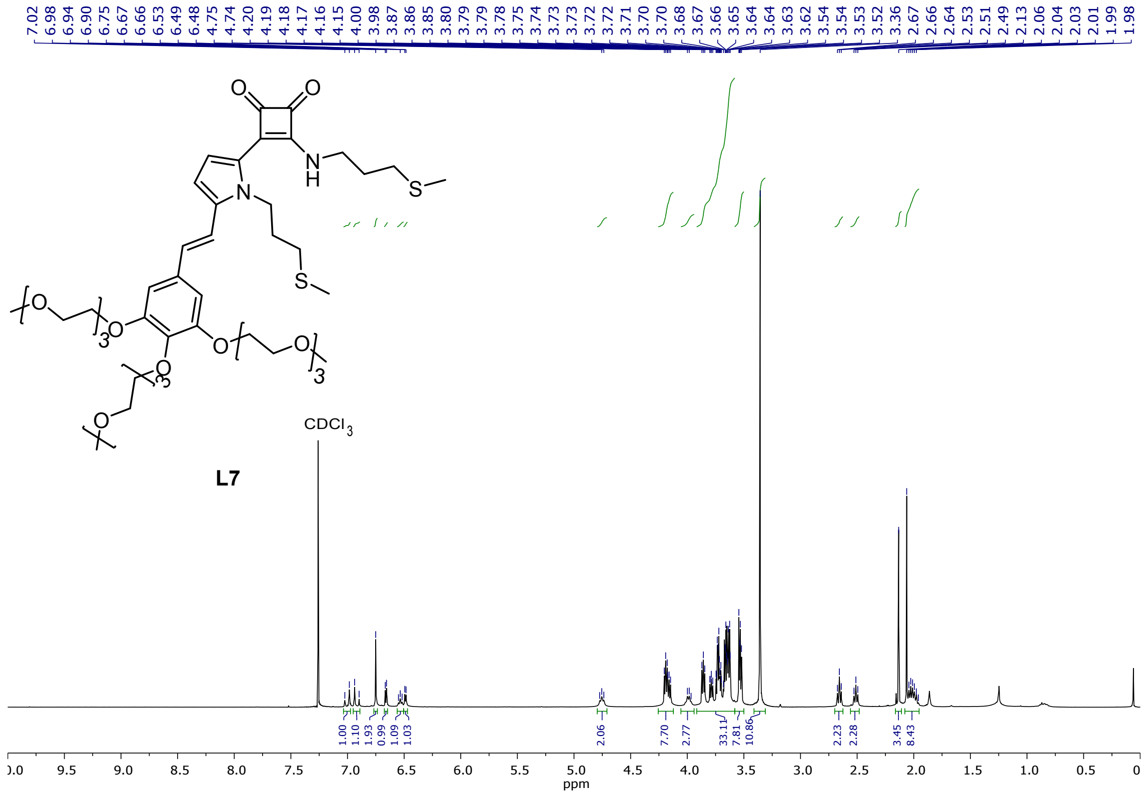

^{1}H-NMR (400 MHz, CDCl$_3$)

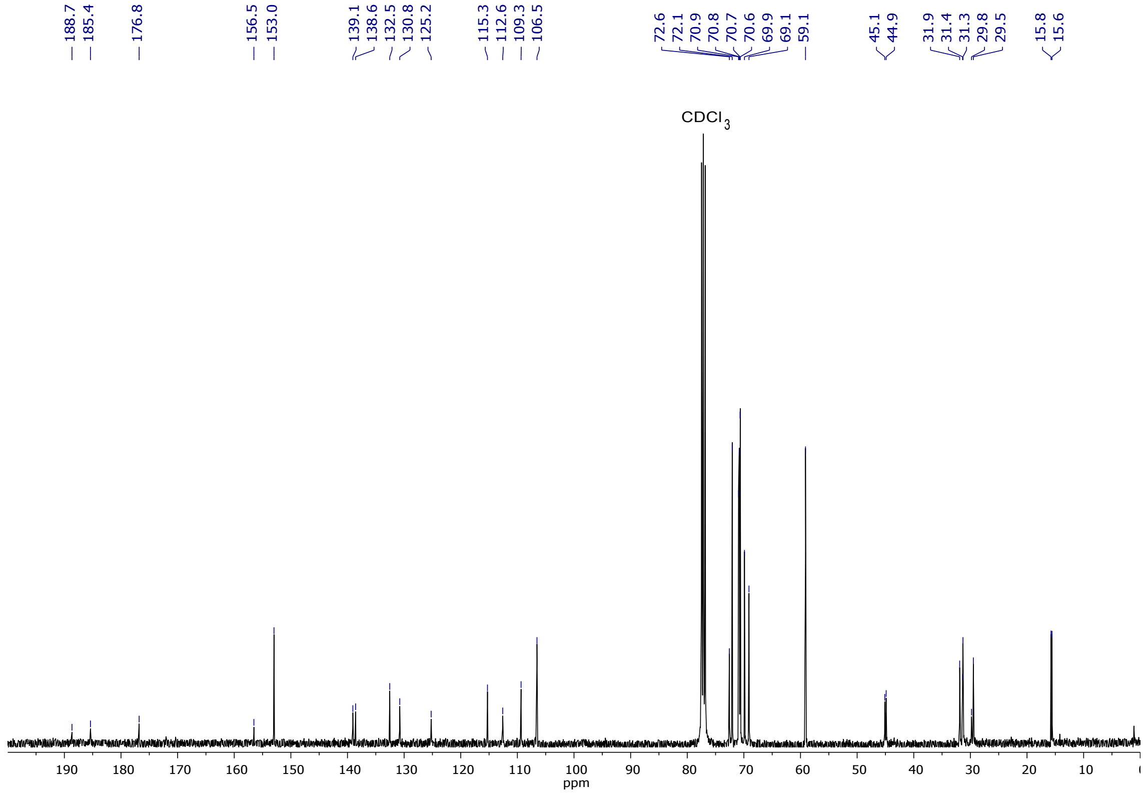

^{13}C-NMR (100 MHz, CDCl$_3$)

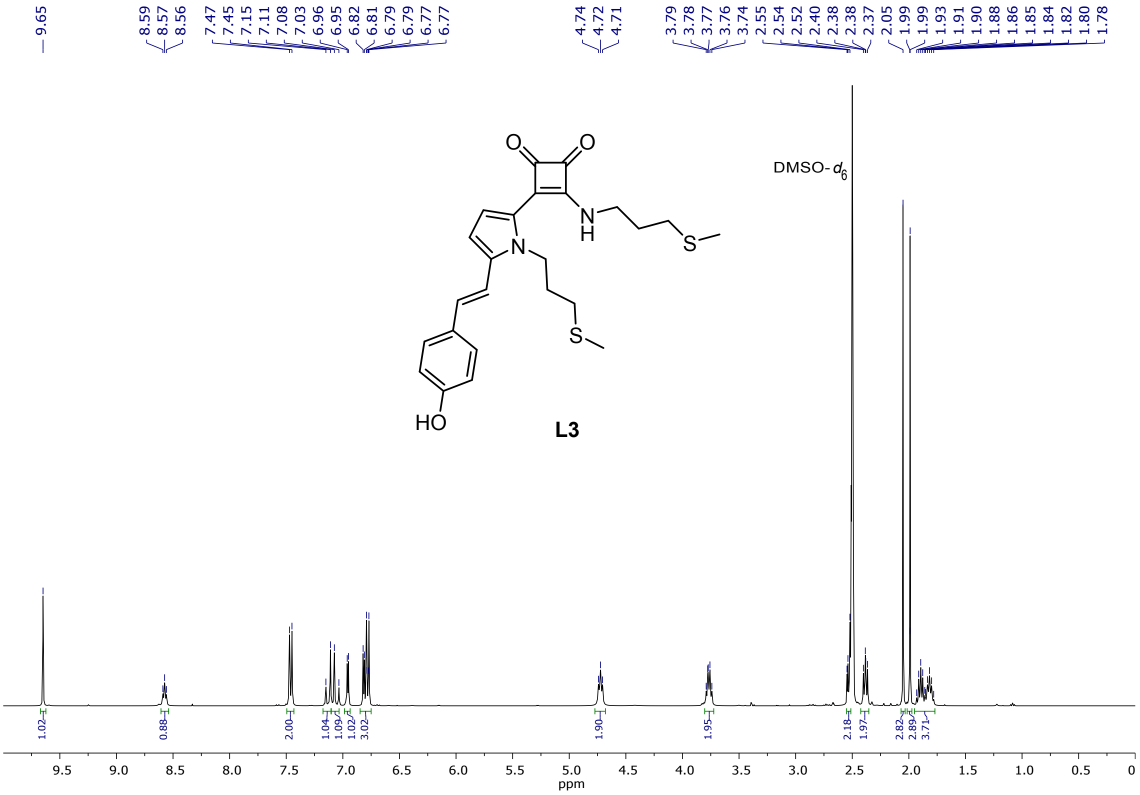

^{1}H-NMR (400 MHz, DMSO-d_6)

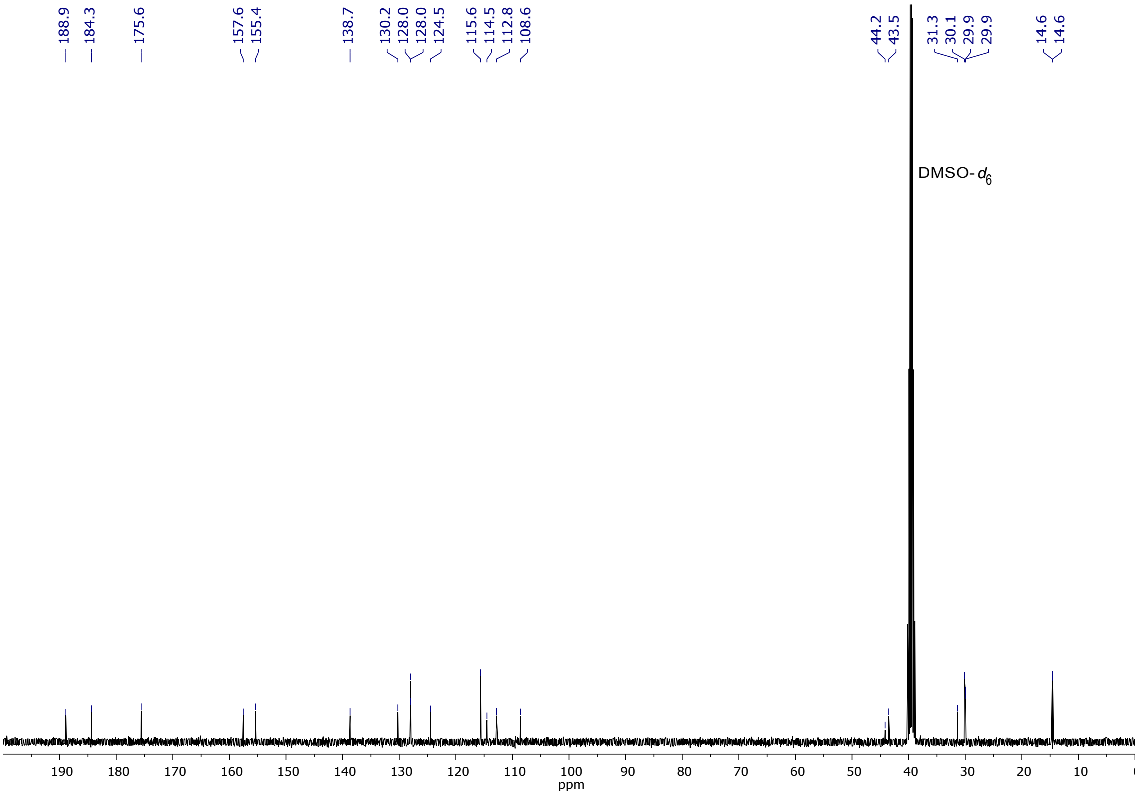

^{13}C-NMR (101 MHz, DMSO-d_6)

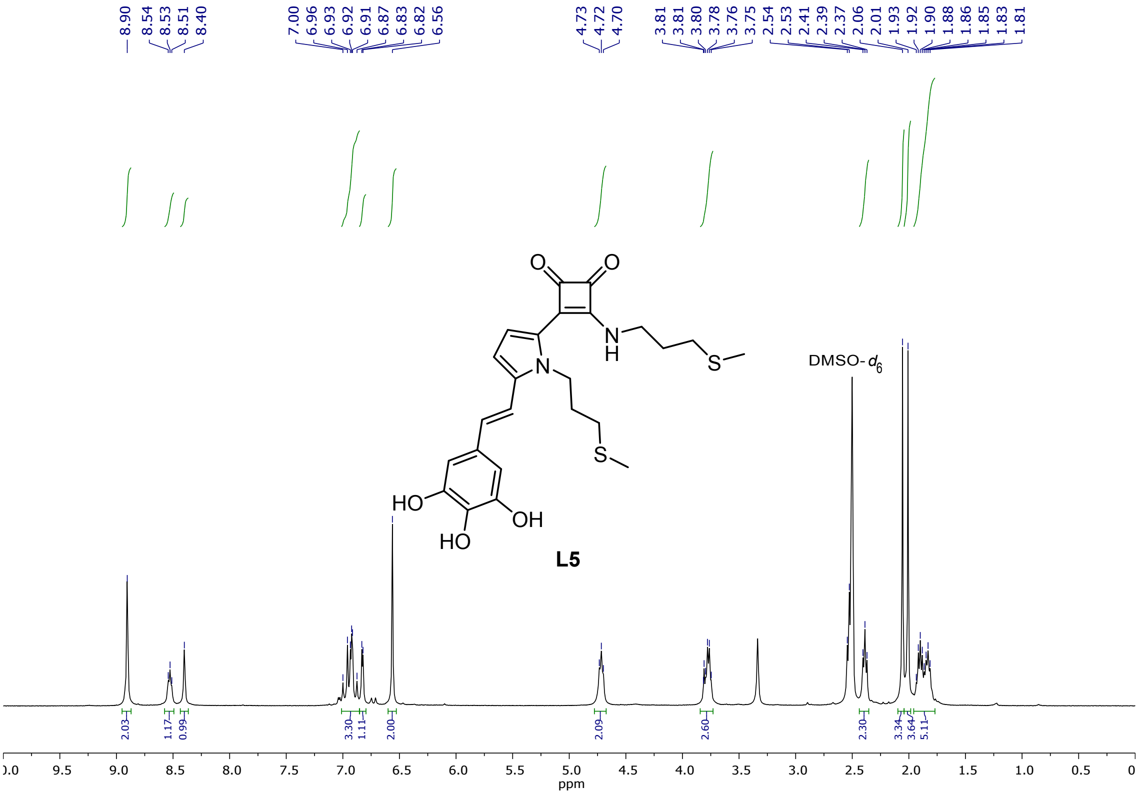

^{1}H-NMR (400 MHz, DMSO-d_6)

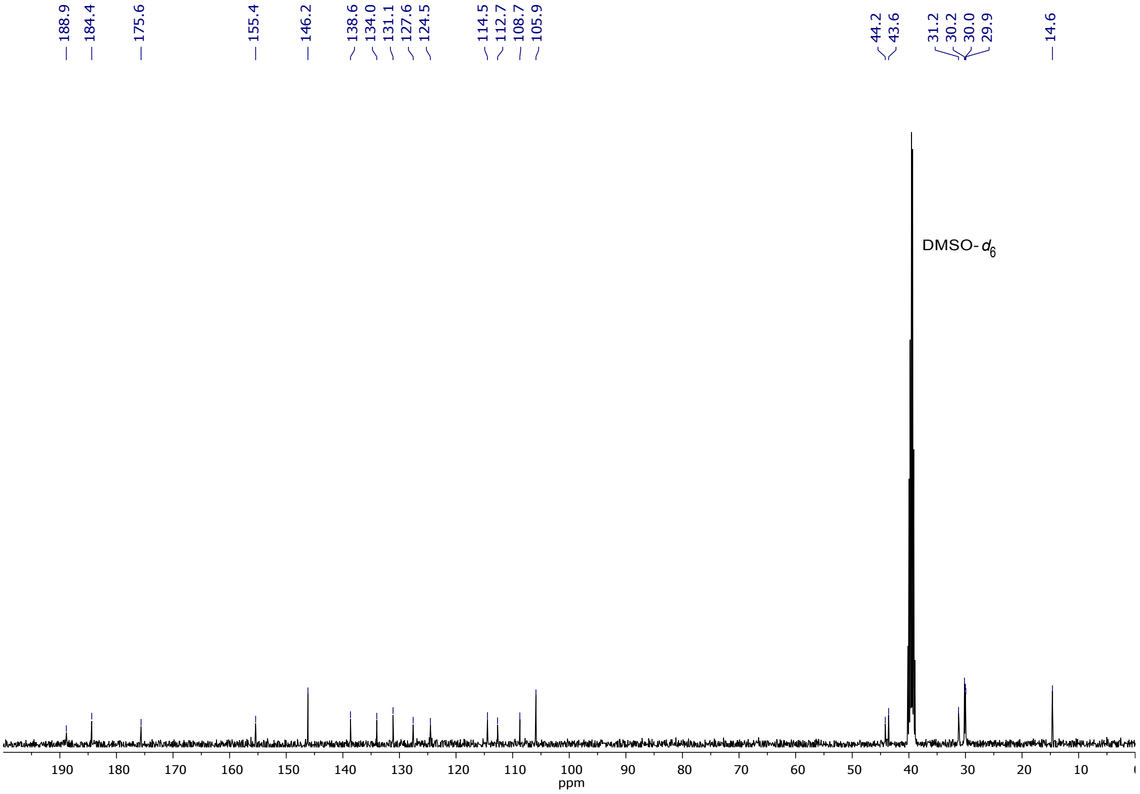

^{13}C-NMR (101 MHz, DMSO-d_6)

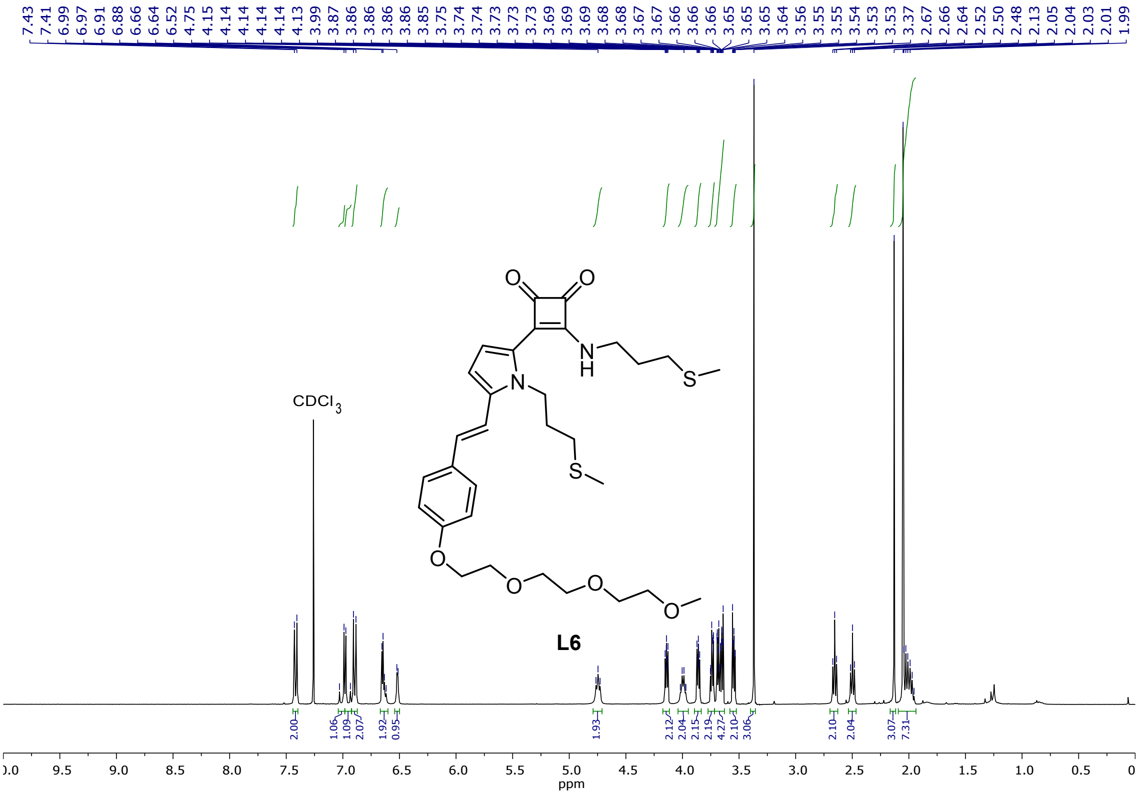

¹H-NMR (400 MHz, $CDCl_3$)

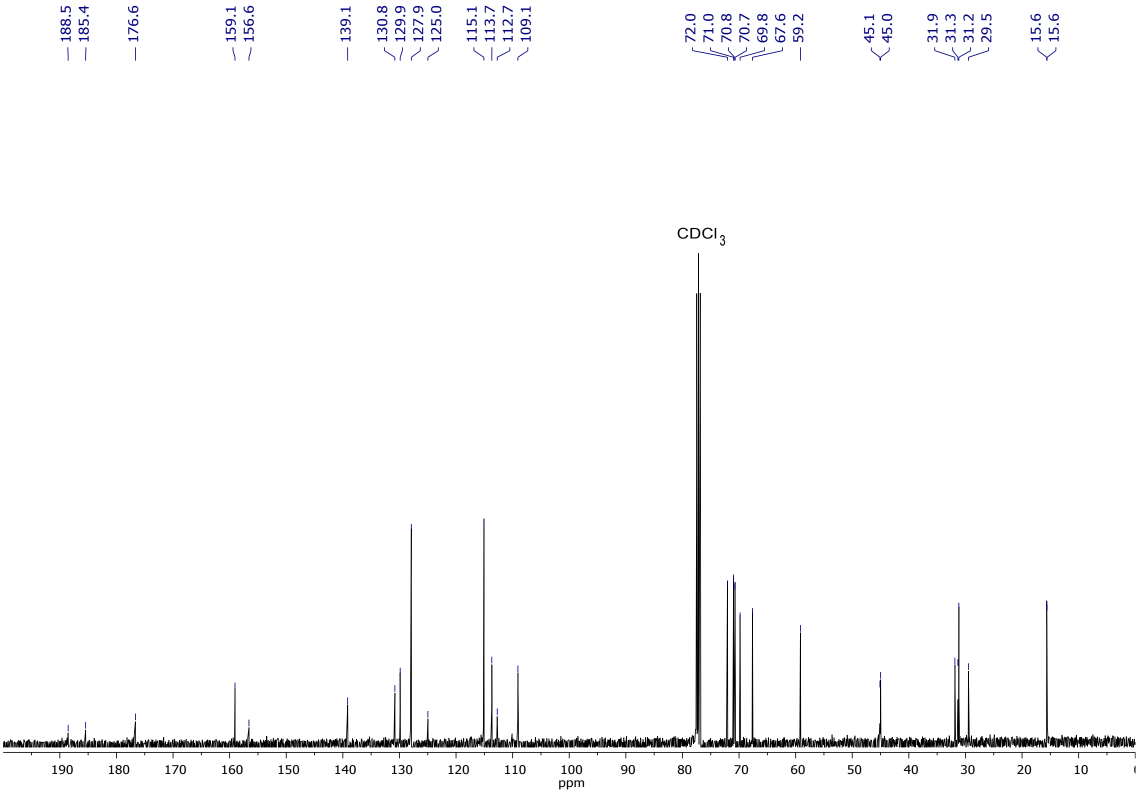

¹³C-NMR (101 MHz, $CDCl_3$)

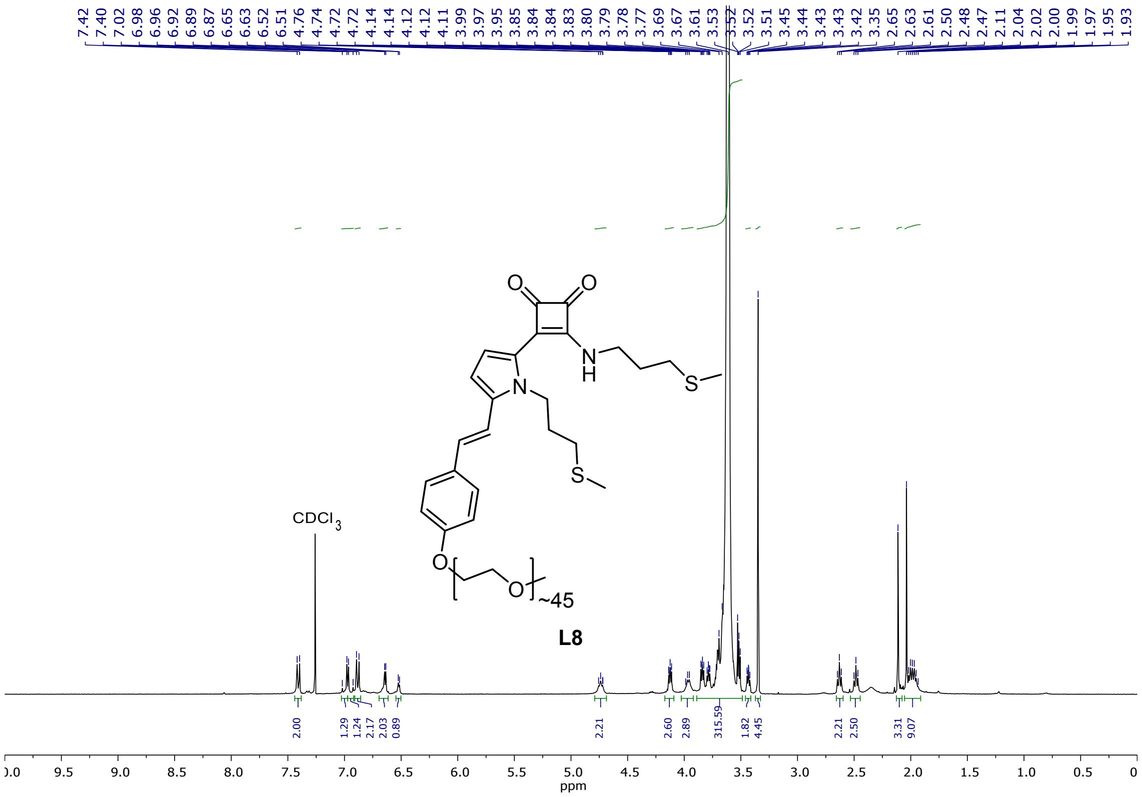

^{1}H-NMR (400 MHz, $CDCl_3$)

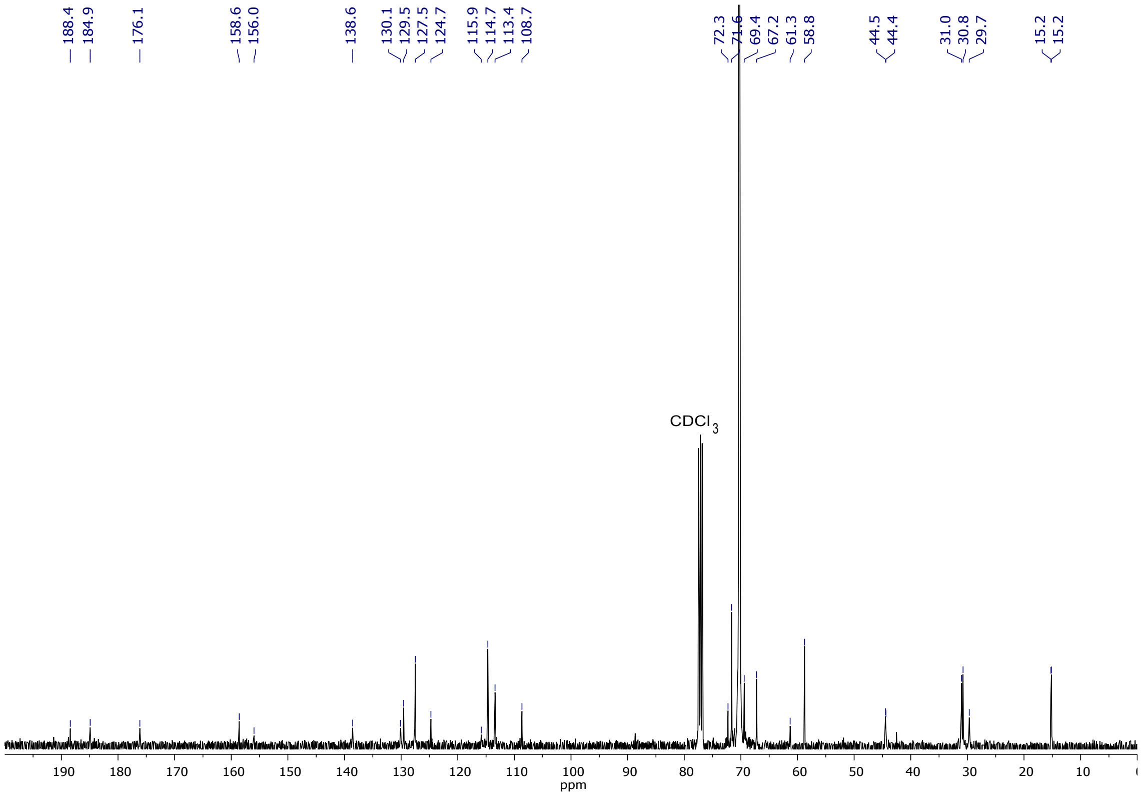

^{13}C-NMR (101 MHz, $CDCl_3$)

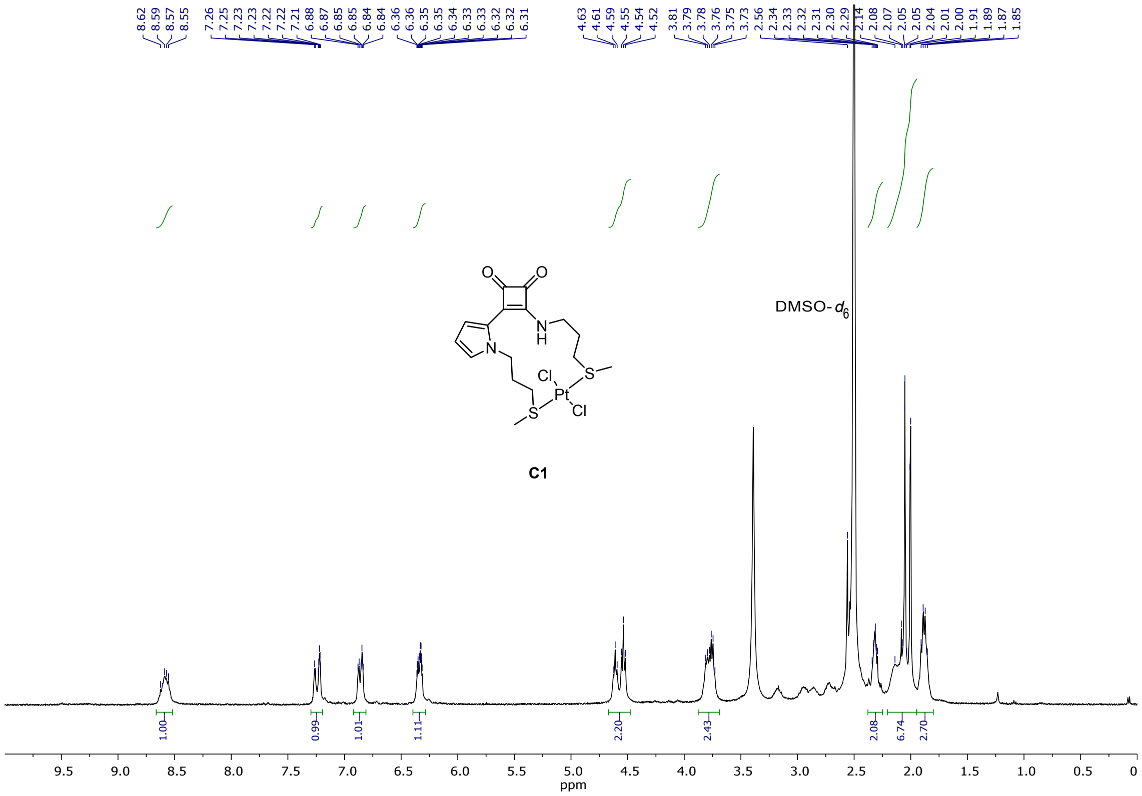

^{1}H-NMR (400 MHz, DMSO-d_6)

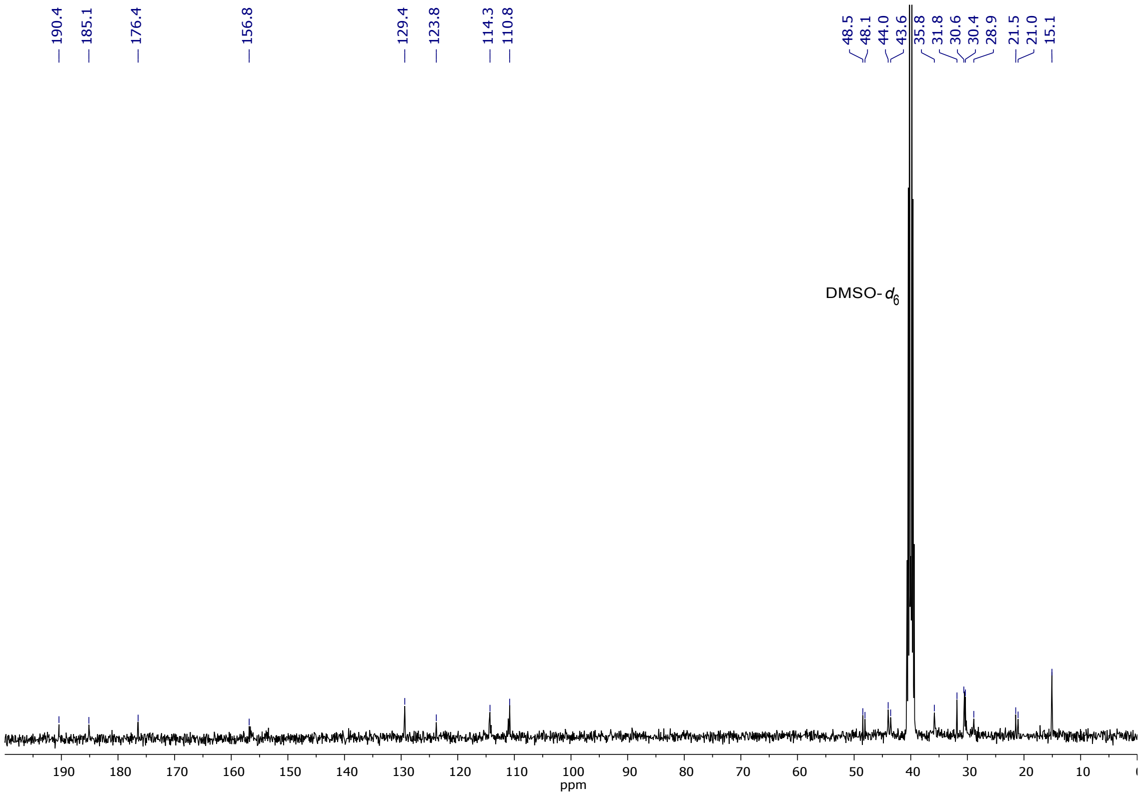

^{13}C-NMR (101 MHz, DMSO-d_6)

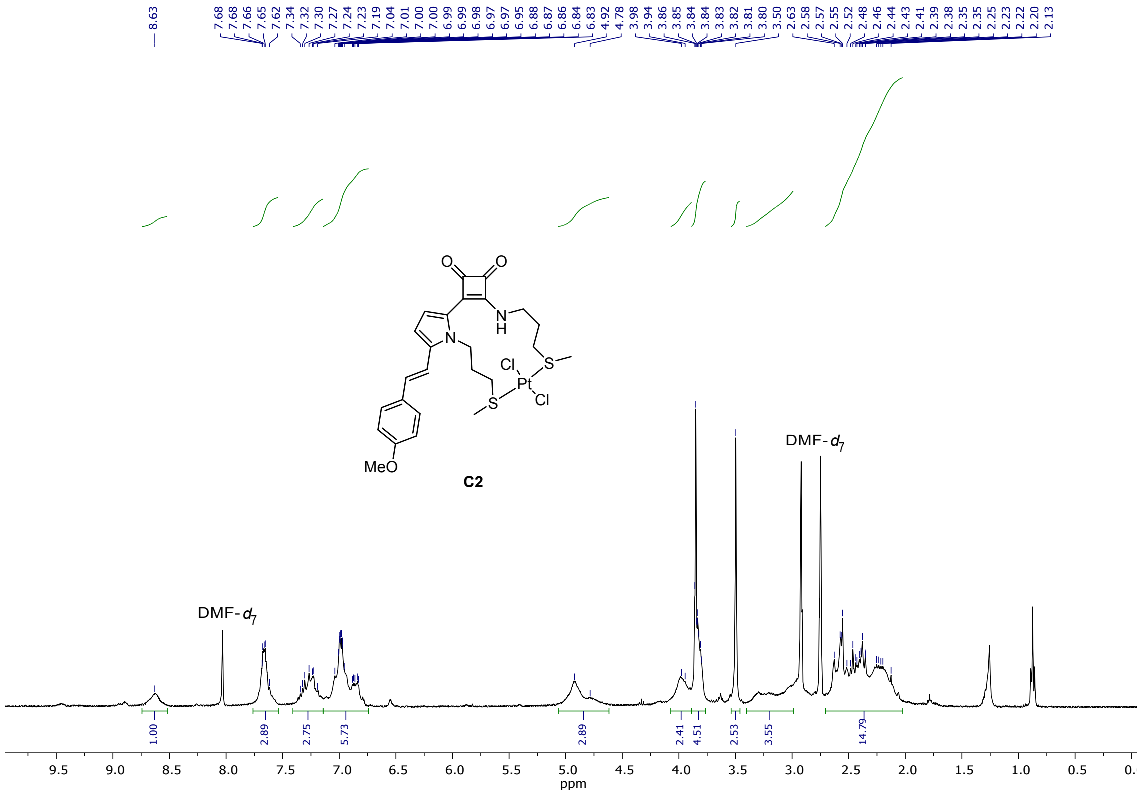

^{1}**H-NMR** (400 MHz, DMF-d_7)

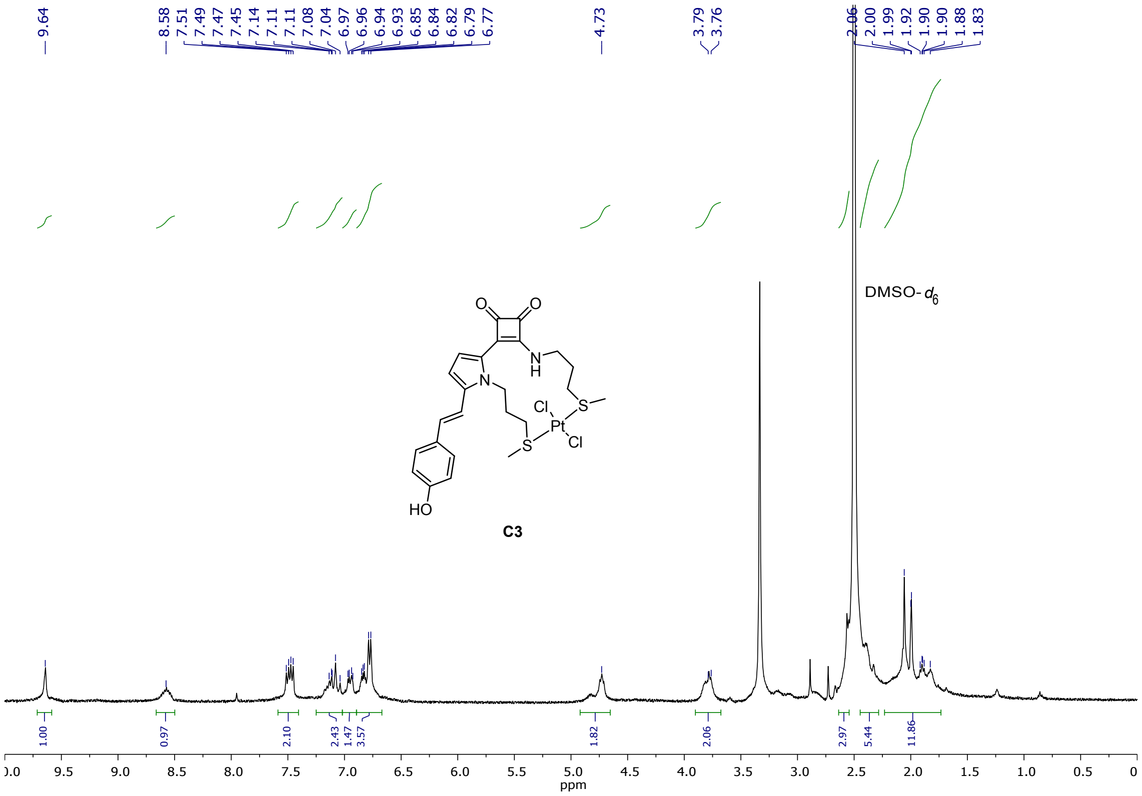

^{1}**H-NMR** (400 MHz, DMSO-d_6)

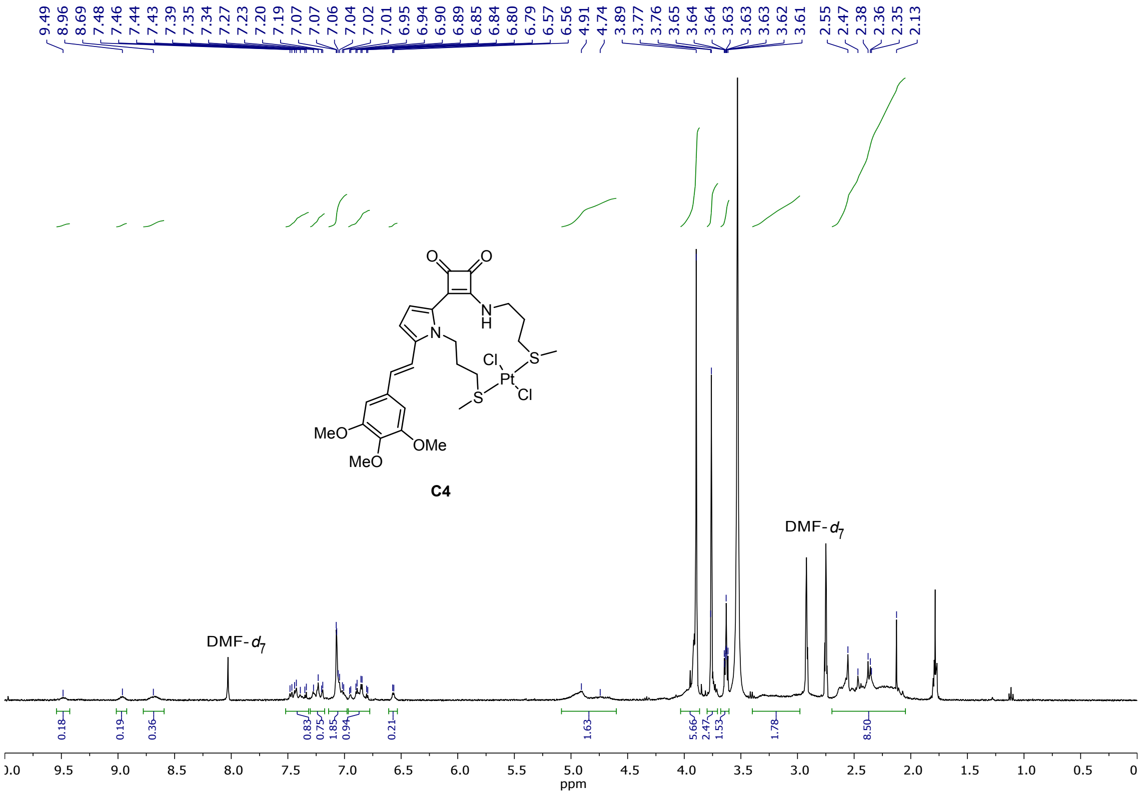

^{1}**H-NMR** (400 MHz, DMF-d_7)

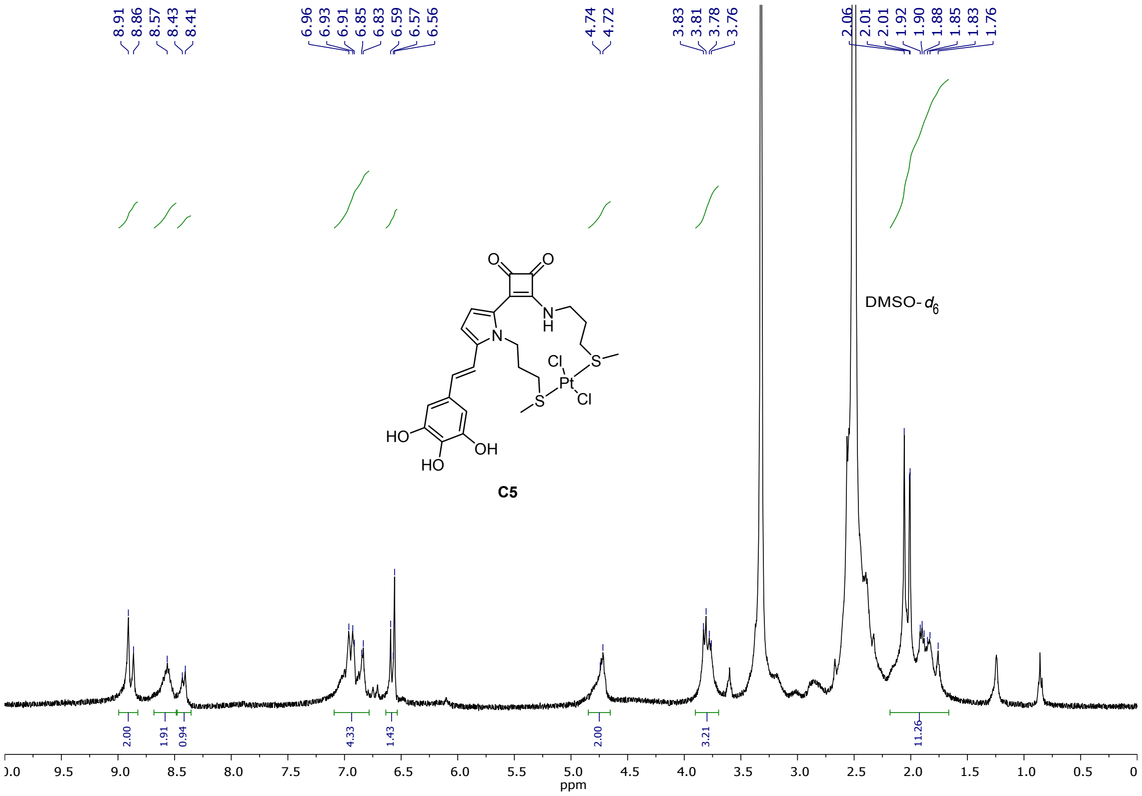

^{1}**H-NMR** (400 MHz, DMSO-d_6)

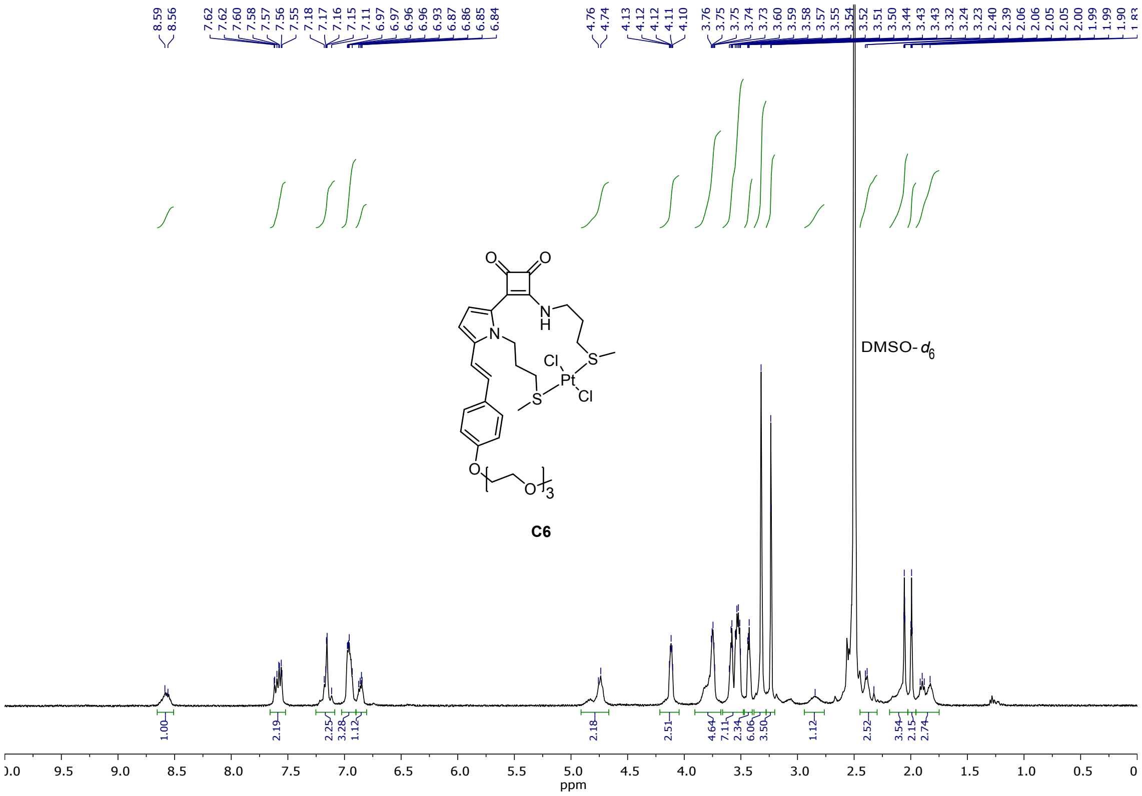

^{1}H-NMR (400 MHz, DMSO-d_6)

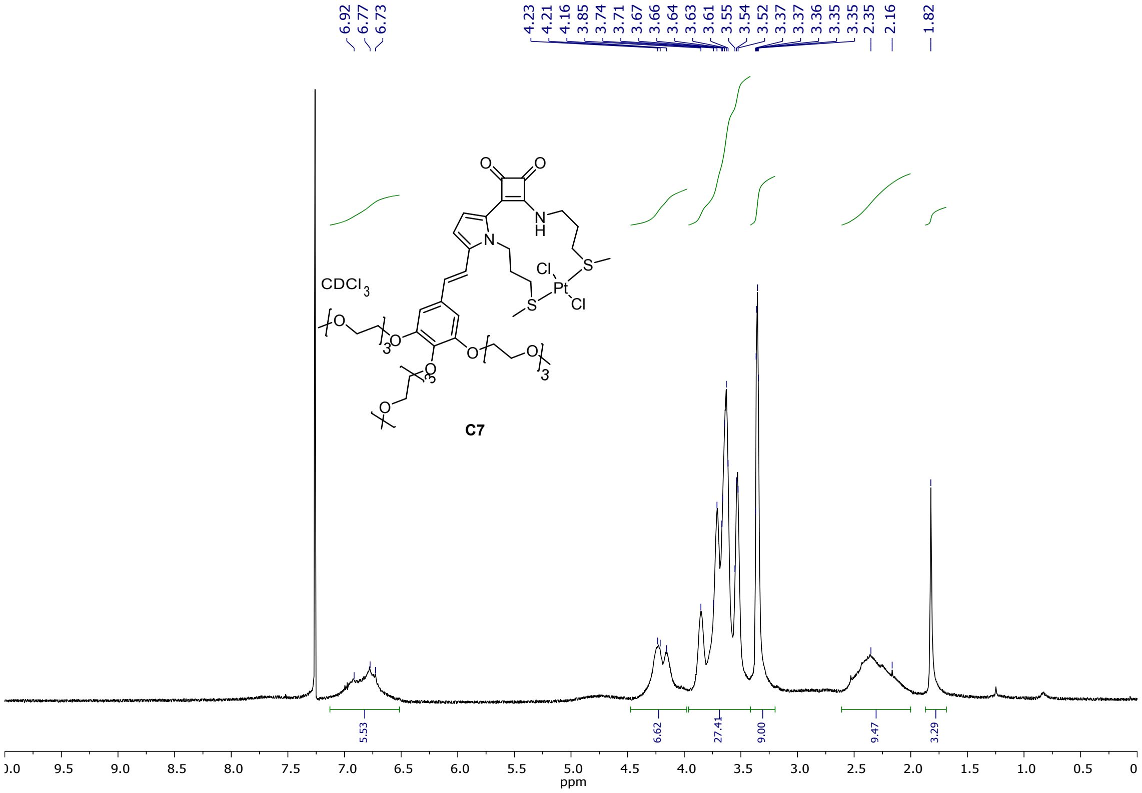

^{1}H-NMR (400 MHz, $CDCl_3$)

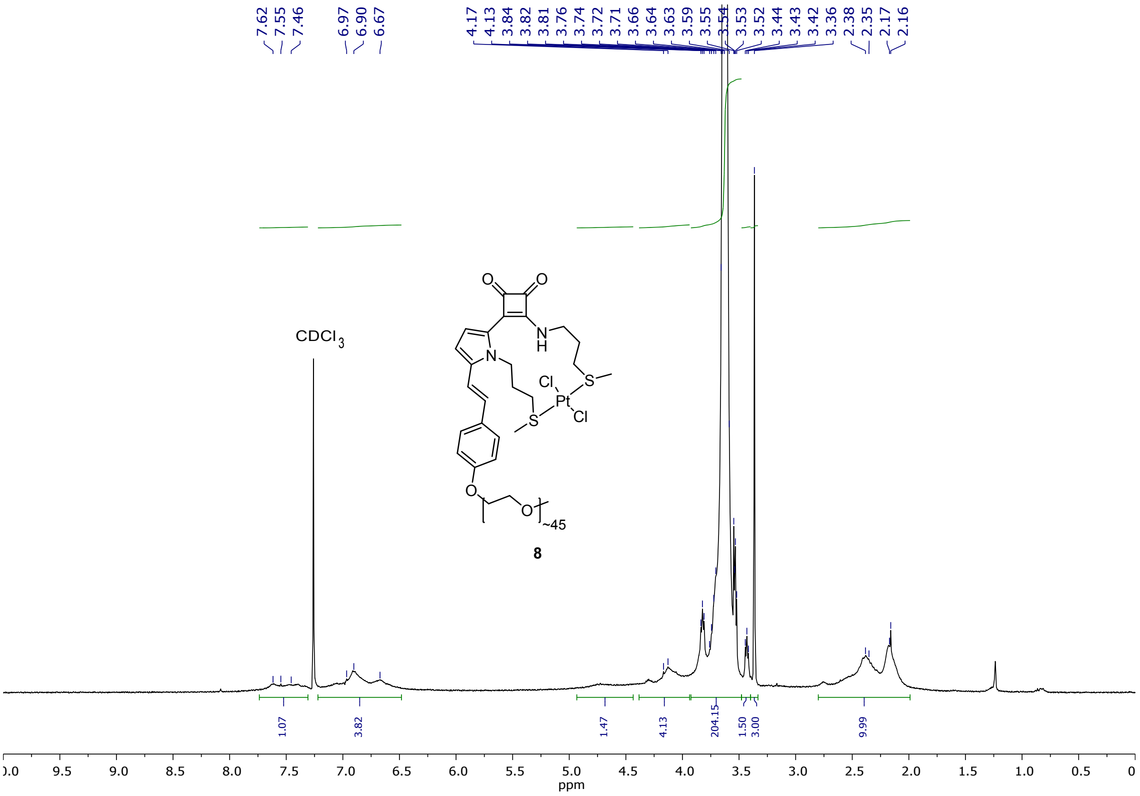

^{1}H-NMR (360 MHz, $CDCl_3$)

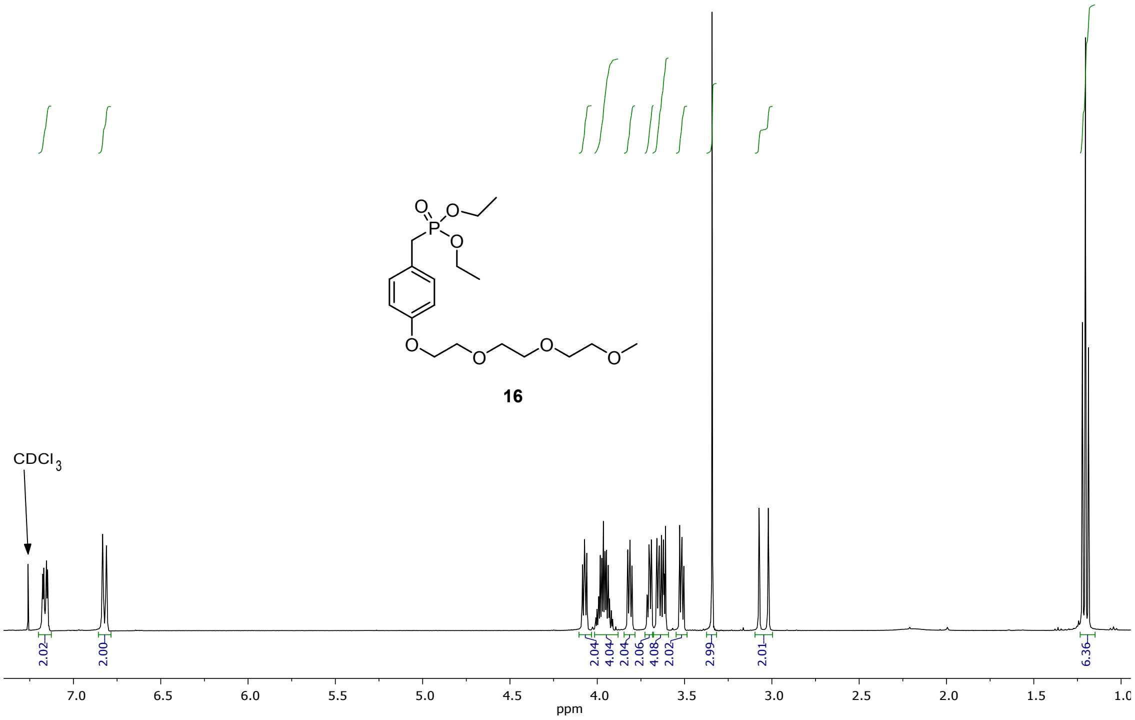

^{1}H-NMR (400 MHz, $CDCl_3$)

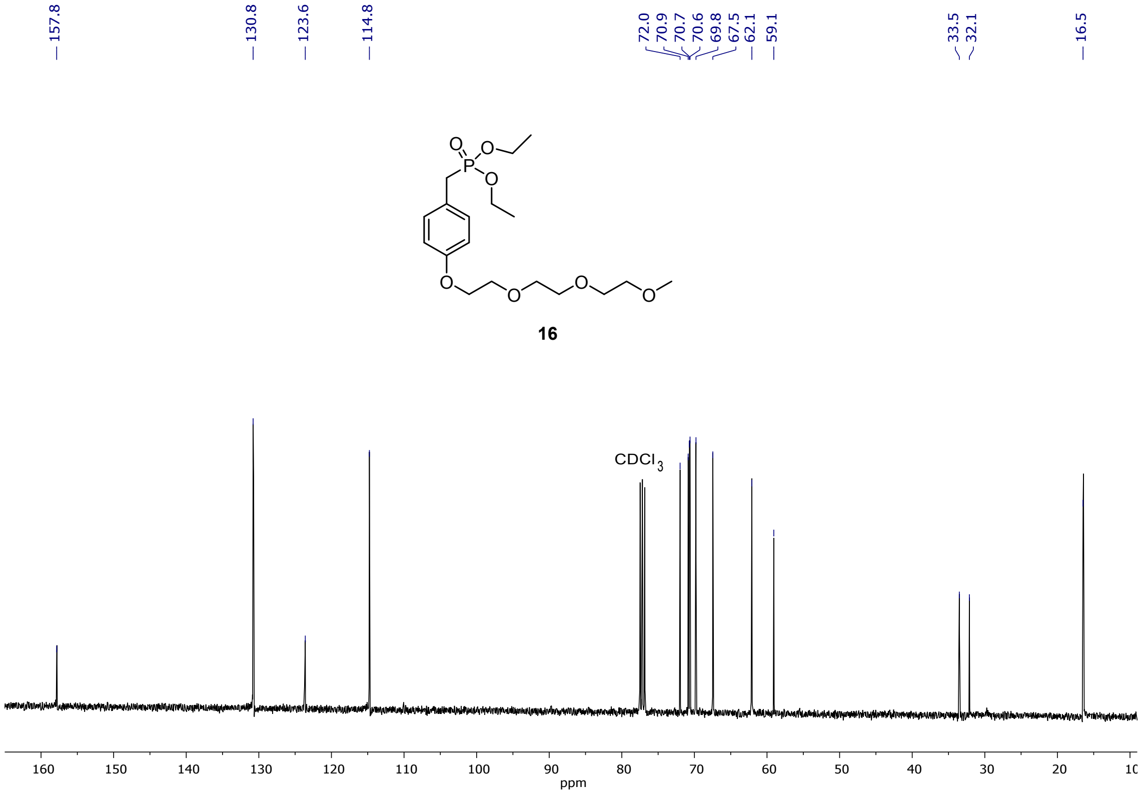

^{13}C-NMR (101 MHz, $CDCl_3$)

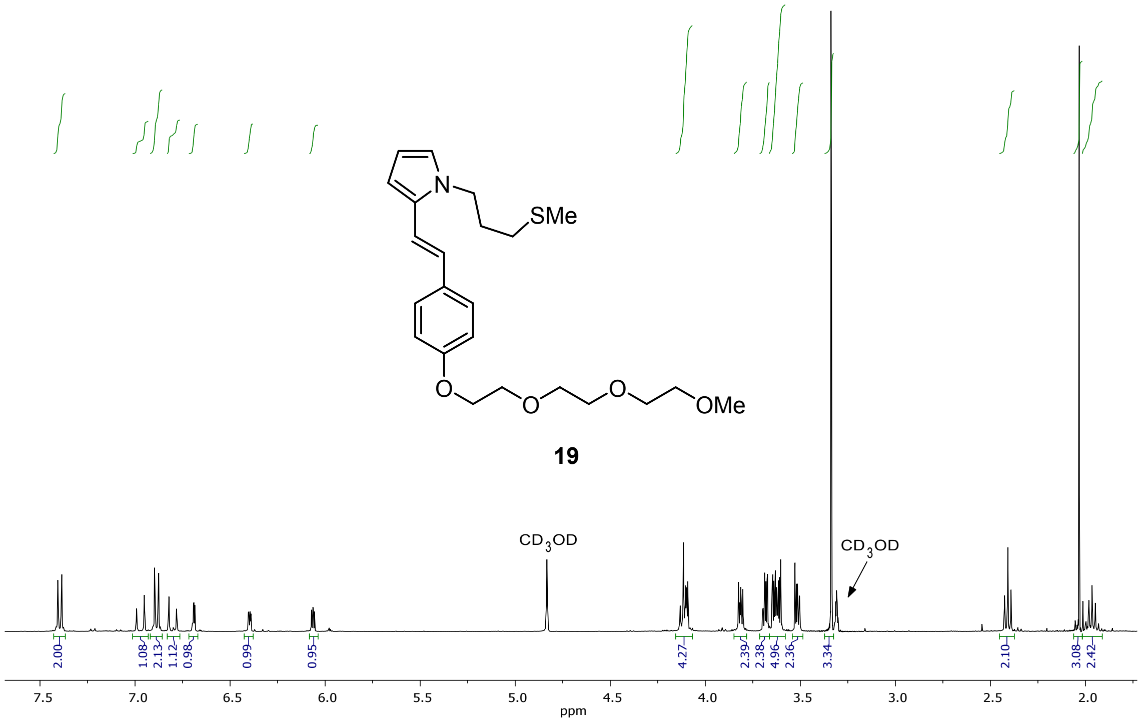

^{1}H-NMR (400 MHz, CD_3OD)

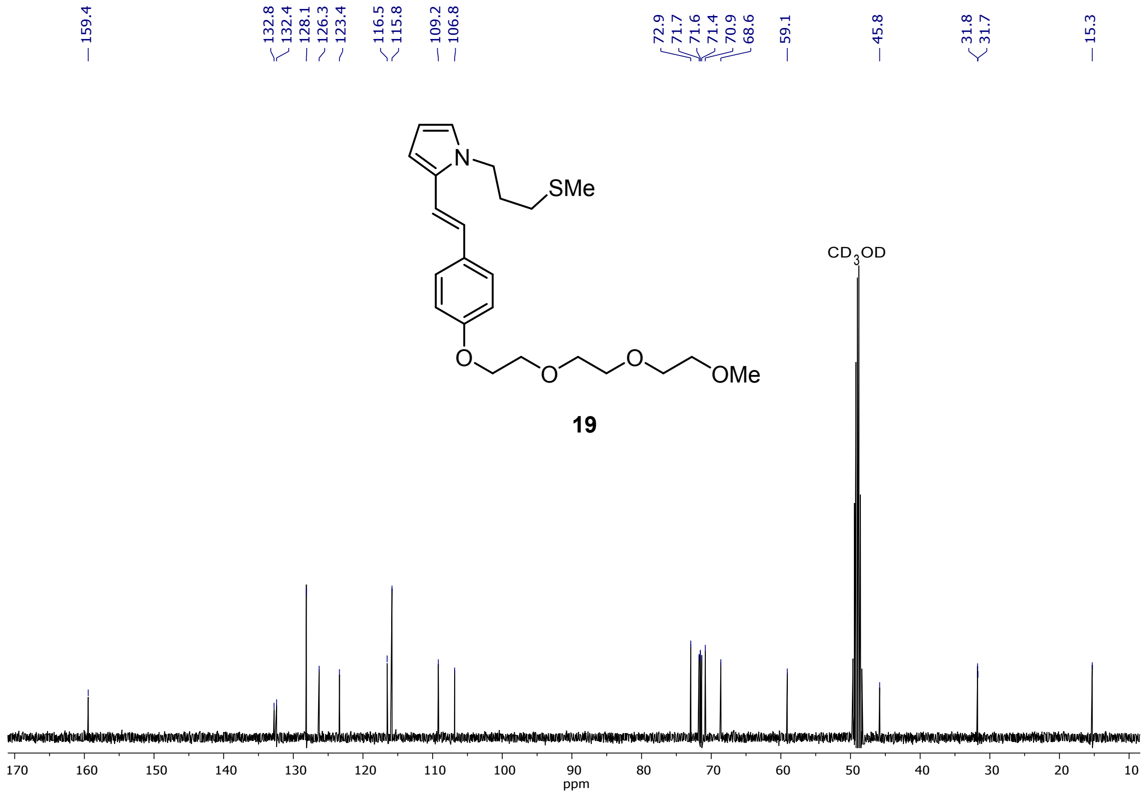

^{13}C-NMR (101 MHz, CD_3OD)

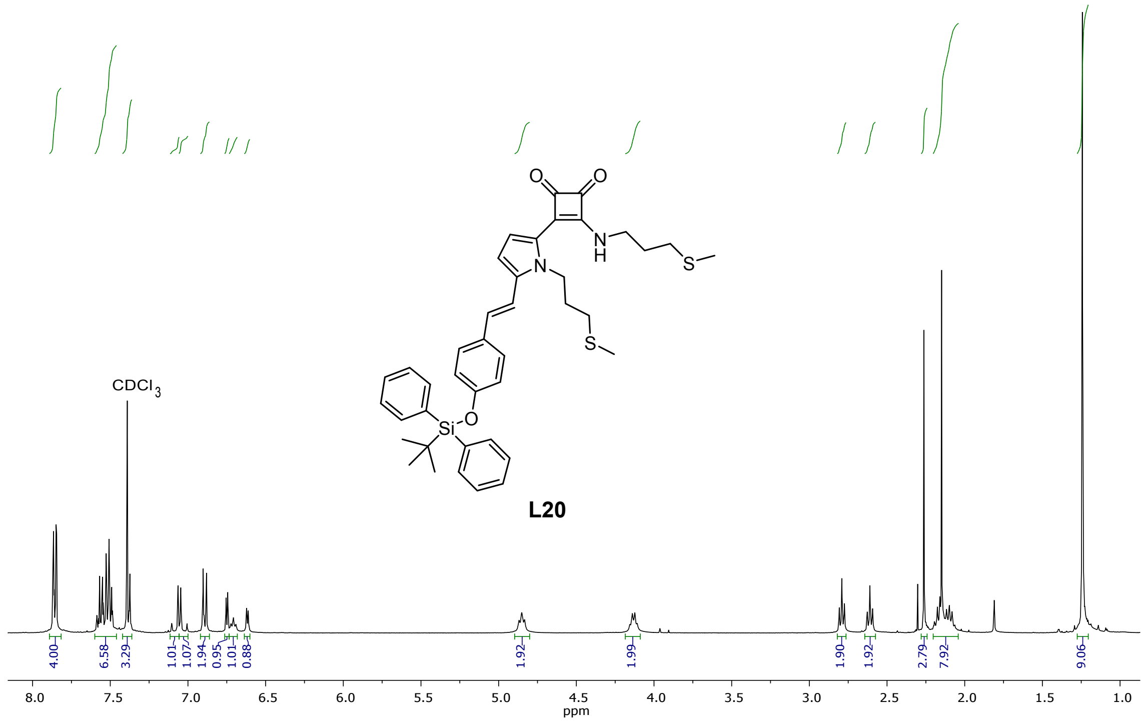

^{1}H-NMR (400 MHz, $CDCl_3$)

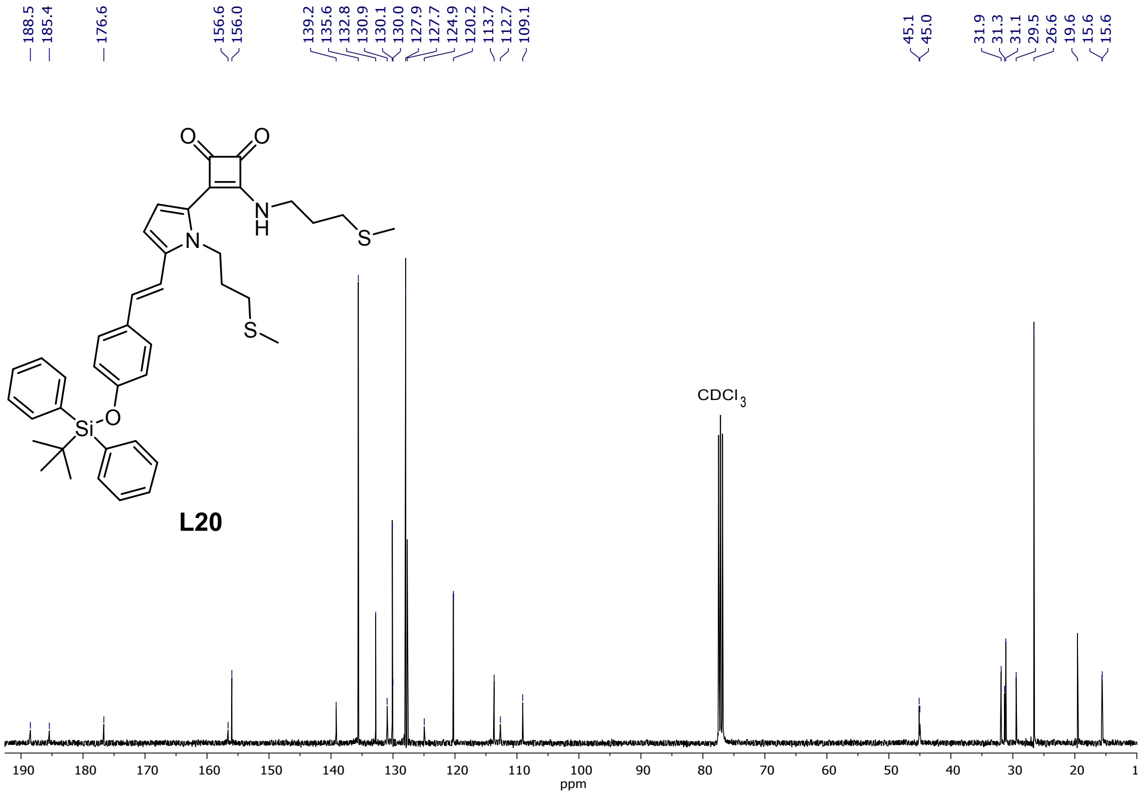

^{13}**C-NMR** (101 MHz, $CDCl_3$)

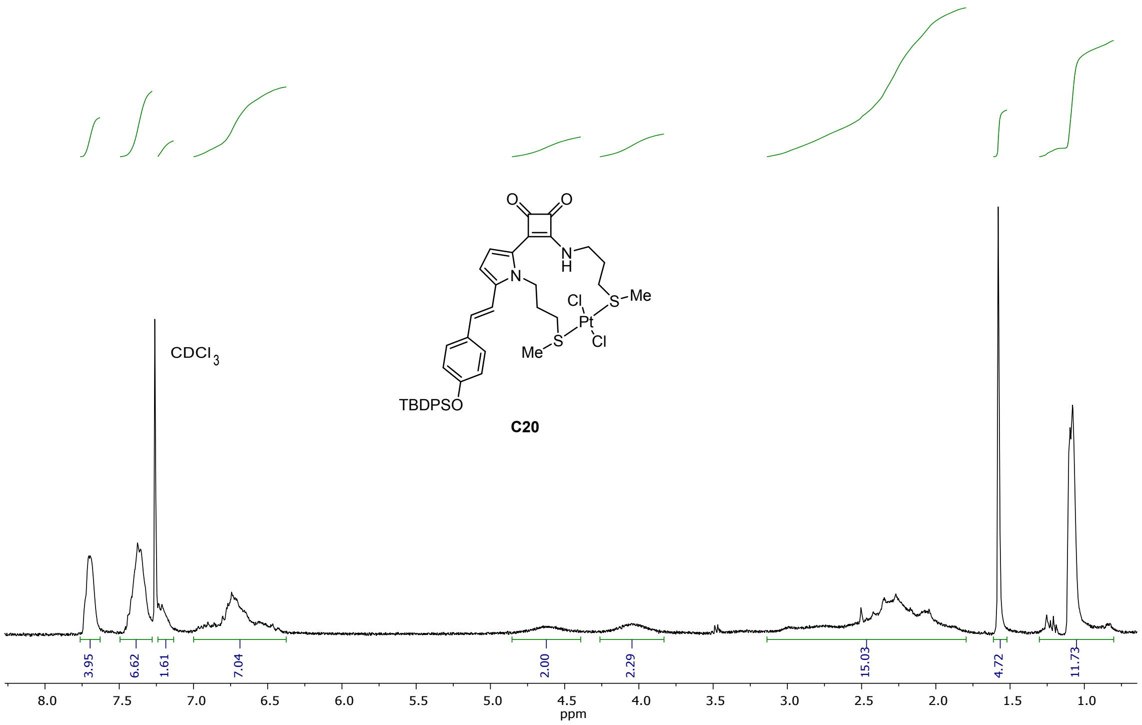

^{1}**H-NMR** (360 MHz, $CDCl_3$)

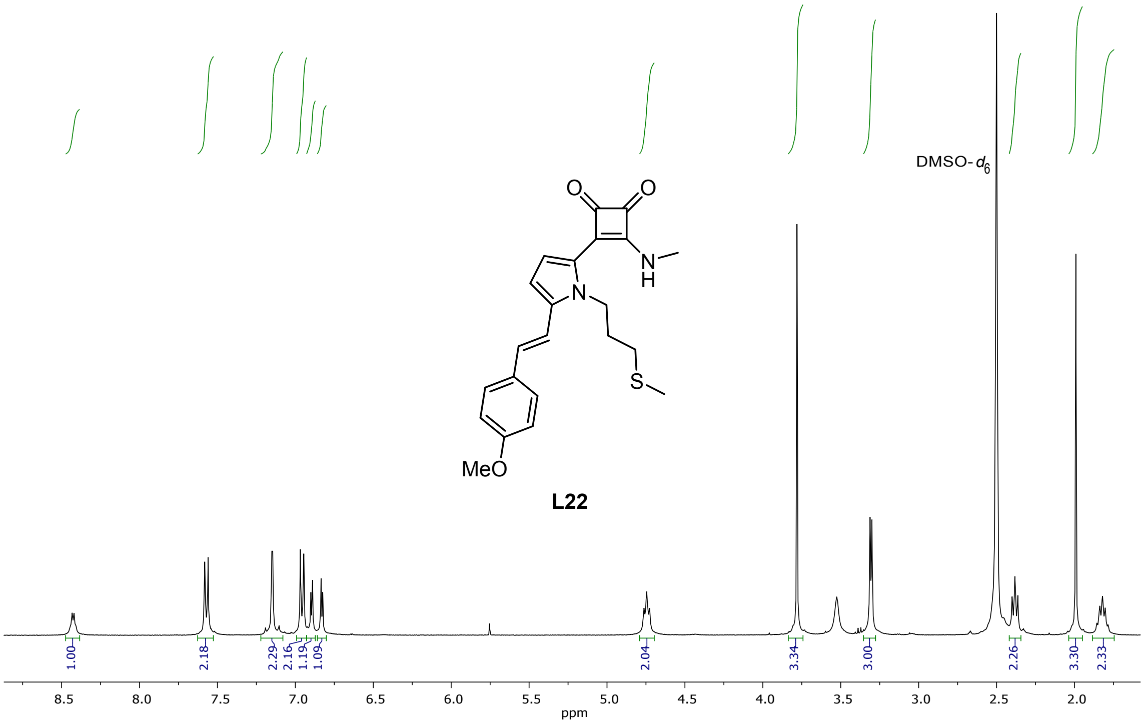

^{1}H-NMR (400 MHz, DMSO-d_6)

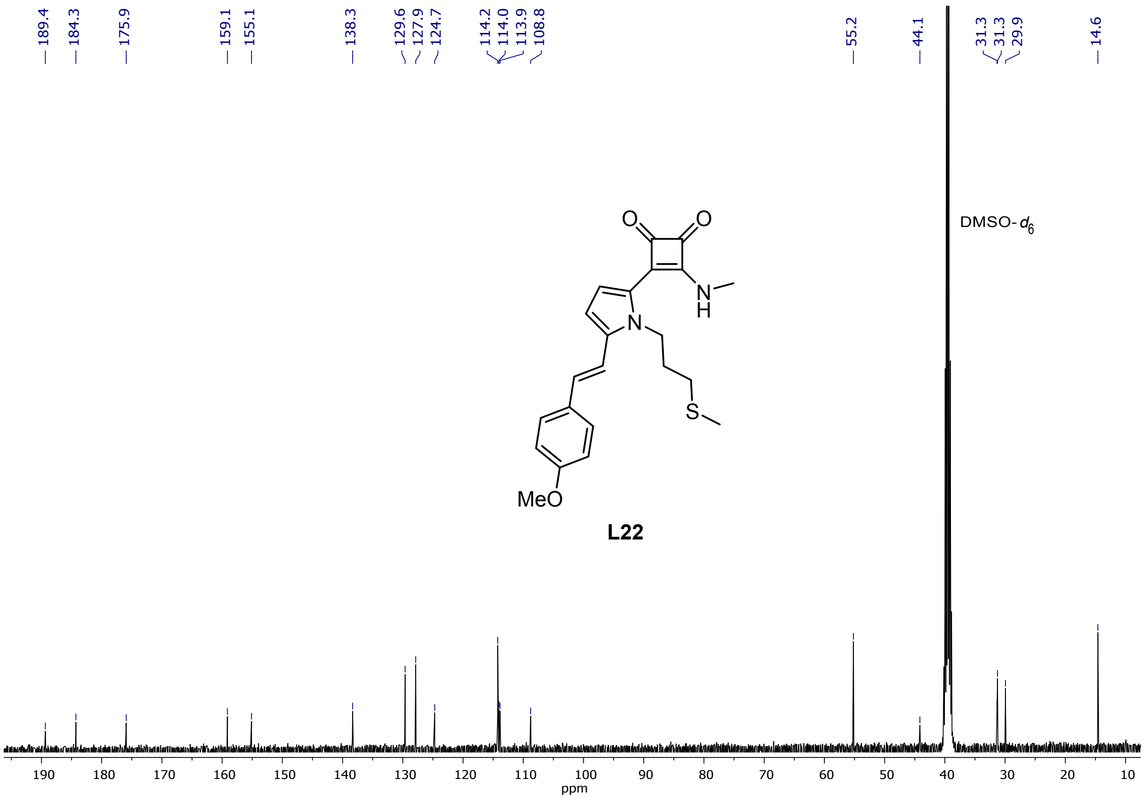

^{13}C-NMR (101 MHz, DMSO-d_6)

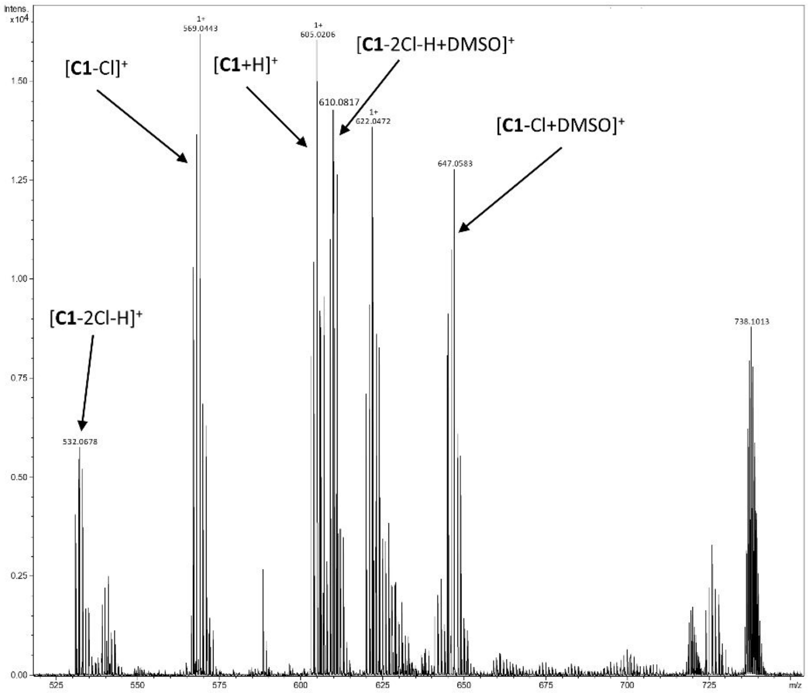

ESI-HRMS spectrum (positive mode) for **C1**

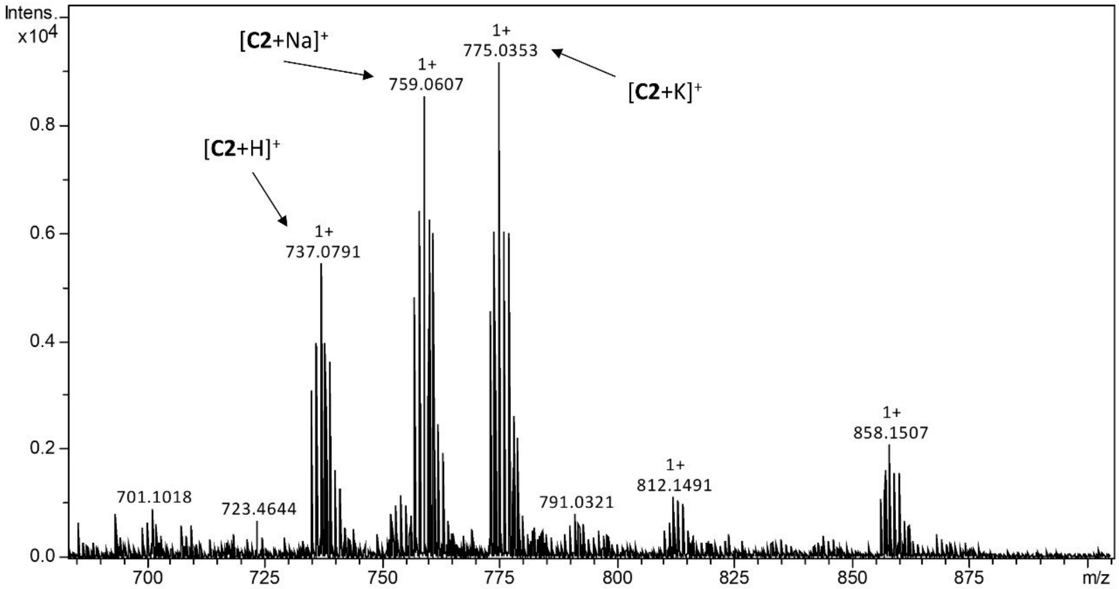

ESI-HRMS spectrum (positive mode) for **C2**

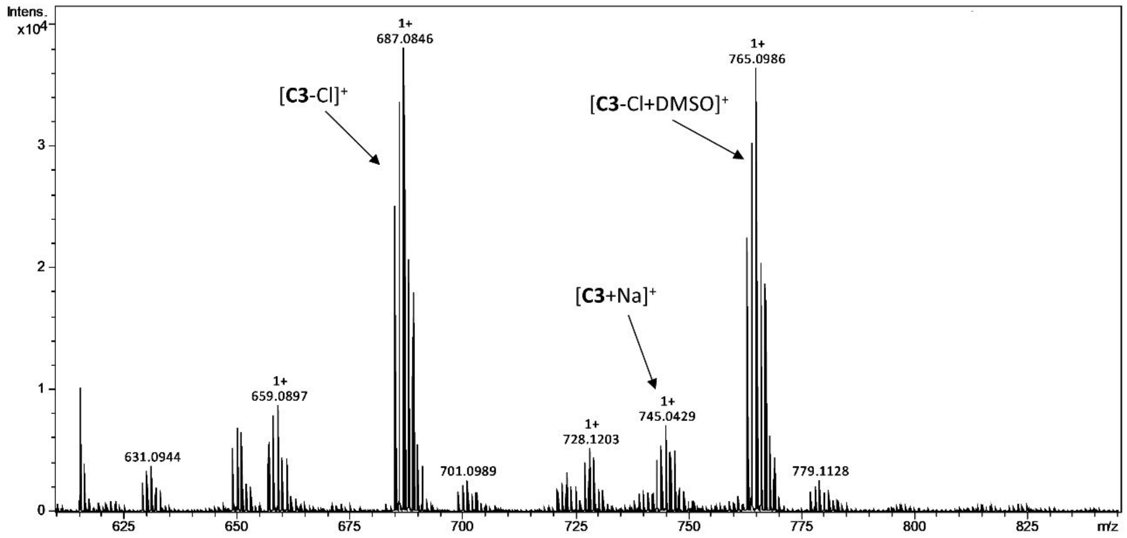

ESI-MS spectrum (positive mode) for **C3**

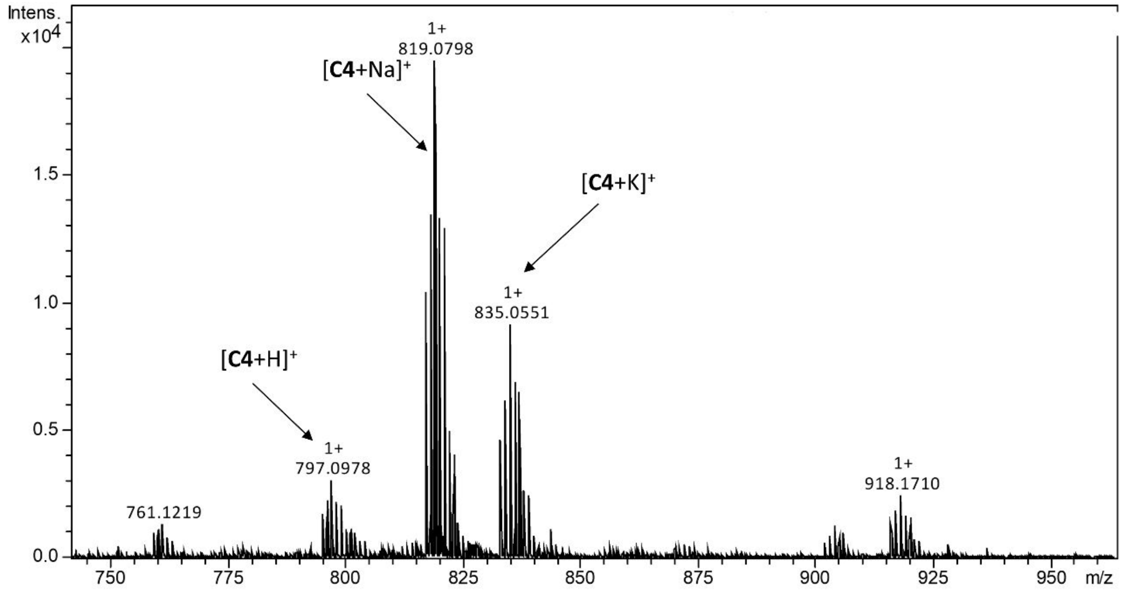

ESI-HRMS spectrum (positive mode) for **C4**

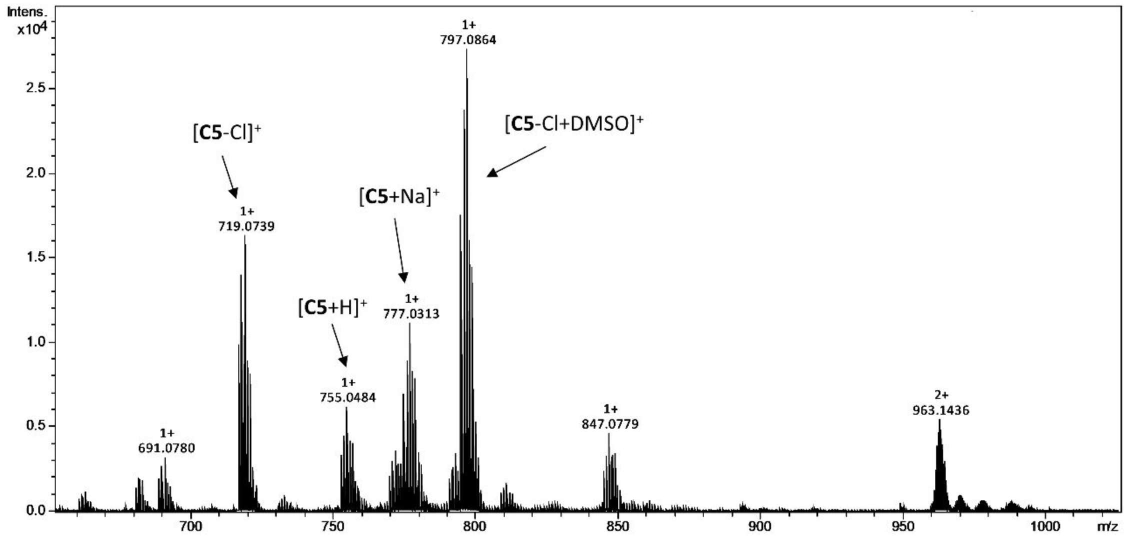

ESI-MS spectrum (positive mode) for **C5**

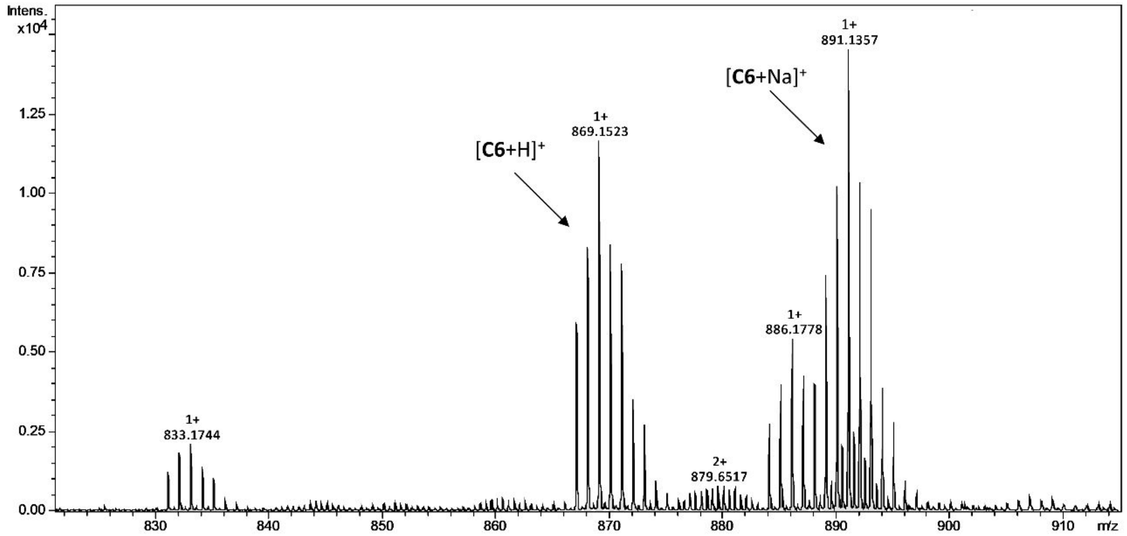

ESI-MS spectrum (positive mode) for **C6**

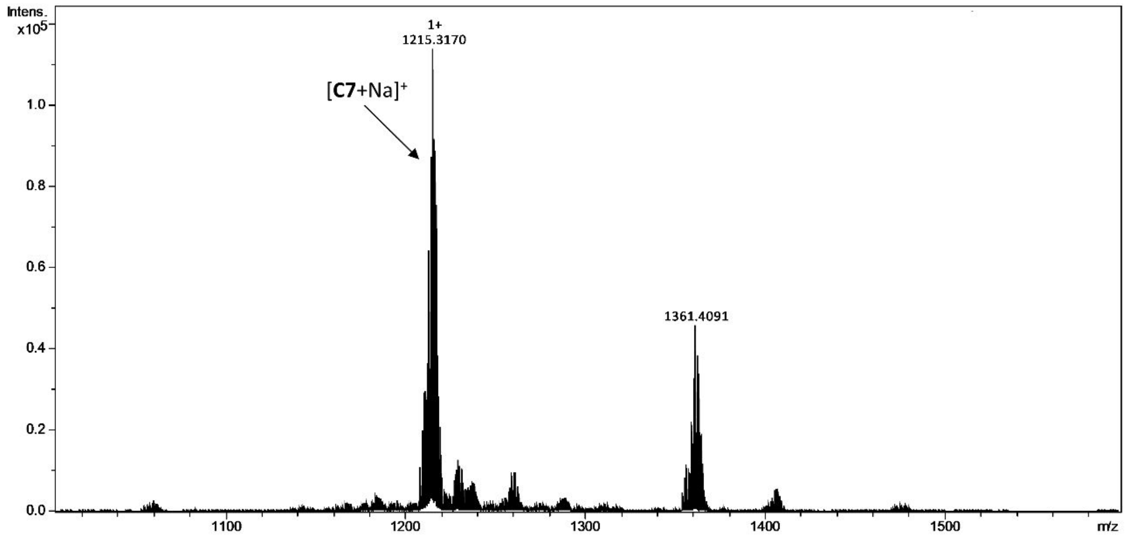

ESI-MS spectrum (positive mode) for **C7**

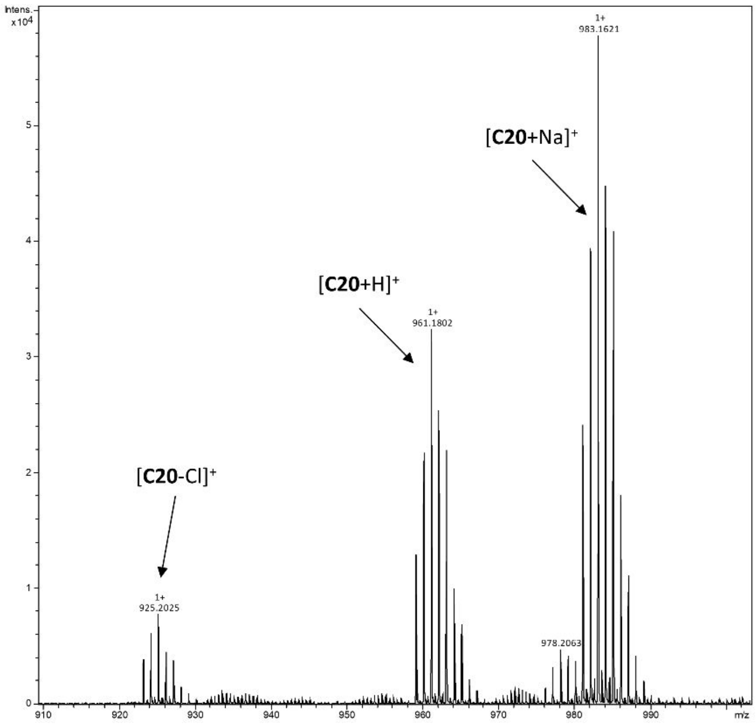

ESI-MS spectrum (positive mode) for **C20**

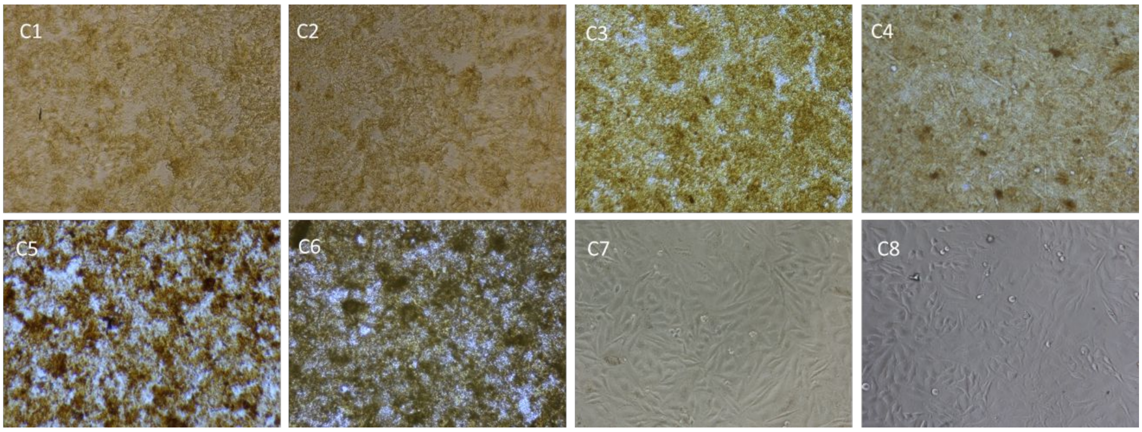

Figure S12. Micrographs of complex-treated (**C1-8**) A2780 cells with 10% FBS-containing cell culture medium.

Table S4. CIF File for compound **L2**.

```
data_L2
_audit_creation_date              2021-07-22
_audit_creation_method
;
Olex2 1.3
(compiled 2020.11.12 svn.r5f609507 for OlexSys, GUI svn.r6272)
;
_shelx_SHELXL_version_number      '2018/3'
_audit_contact_author_address     ?
_audit_contact_author_email       ?
_audit_contact_author_name        ''
_audit_contact_author_phone       ?
_publ_contact_author_id_orcid     ?
_publ_section_references
;
Dolomanov, O.V., Bourhis, L.J., Gildea, R.J, Howard, J.A.K. &
Puschmann, H.
 (2009), J. Appl. Cryst. 42, 339-341.

Sheldrick, G.M. (2008). Acta Cryst. A64, 112-122.
;
_chemical_name_common             ?
_chemical_name_systematic         ?
_chemical_formula_moiety          'C25 H30 N2 O3 S2'
_chemical_formula_sum             'C25 H30 N2 O3 S2'
_chemical_formula_weight          470.63
_chemical_melting_point           ?
loop_
  _atom_type_symbol
  _atom_type_description
  _atom_type_scat_dispersion_real
  _atom_type_scat_dispersion_imag
  _atom_type_scat_source
 'C' 'C' 0.0181 0.0091 'International Tables Vol C Tables 4.2.6.8
and 6.1.1.4'
 'H' 'H' 0.0000 0.0000 'International Tables Vol C Tables 4.2.6.8
and 6.1.1.4'
 'N' 'N' 0.0311 0.0180 'International Tables Vol C Tables 4.2.6.8
and 6.1.1.4'
 'O' 'O' 0.0492 0.0322 'International Tables Vol C Tables 4.2.6.8
and 6.1.1.4'
 'S' 'S' 0.3331 0.5567 'International Tables Vol C Tables 4.2.6.8
and 6.1.1.4'

_shelx_space_group_comment
;
The symmetry employed for this shelxl refinement is uniquely
defined
by the following loop, which should always be used as a source of
symmetry information in preference to the above space-group names.
They are only intended as comments.
;
_space_group_crystal_system       'monoclinic'
_space_group_IT_number            14
_space_group_name_H-M_alt         'P2(1)/c'
_space_group_name_Hall            '-P 2ybc'
```

```
loop_
  _space_group_symop_operation_xyz
 'x, y, z'
 '-x, y+1/2, -z+1/2'
 '-x, -y, -z'
 'x, -y-1/2, z-1/2'

_cell_length_a                    6.92670(10)
_cell_length_b                    15.2298(3)
_cell_length_c                    23.2875(4)
_cell_angle_alpha                 90
_cell_angle_beta                  90.7950(10)
_cell_angle_gamma                 90
_cell_volume                      2456.41(7)
_cell_formula_units_Z             4
_cell_measurement_reflns_used     8902
_cell_measurement_temperature     298(2)
_cell_measurement_theta_max       66.271
_cell_measurement_theta_min       3.467
_shelx_estimated_absorpt_T_max    0.811
_shelx_estimated_absorpt_T_min    0.779
_exptl_absorpt_coefficient_mu     2.193
_exptl_absorpt_correction_T_max   0.7528
_exptl_absorpt_correction_T_min   0.6087
_exptl_absorpt_correction_type    multi-scan
_exptl_absorpt_process_details
;
SADABS-2016/2 (Bruker,2016/2) was used for absorption correction.
wR2(int) was 0.1456 before and 0.0532 after correction.
The Ratio of minimum to maximum transmission is 0.8086.
The \l/2 correction factor is Not present.
;
_exptl_absorpt_special_details     ?
_exptl_crystal_colour             yellow
_exptl_crystal_colour_primary     yellow
_exptl_crystal_density_diffrn     1.273
_exptl_crystal_density_meas       ?
_exptl_crystal_density_method     ?
_exptl_crystal_description        block
_exptl_crystal_F_000              1000
_exptl_crystal_size_max           0.12
_exptl_crystal_size_mid           0.11
_exptl_crystal_size_min           0.1
_exptl_transmission_factor_max    ?
_exptl_transmission_factor_min    ?
_diffrn_reflns_av_R_equivalents   0.0447
_diffrn_reflns_av_unetI/netI      0.0274
_diffrn_reflns_Laue_measured_fraction_full  0.995
_diffrn_reflns_Laue_measured_fraction_max  0.995
_diffrn_reflns_limit_h_max        8
_diffrn_reflns_limit_h_min        -7
_diffrn_reflns_limit_k_max        16
_diffrn_reflns_limit_k_min        -18
_diffrn_reflns_limit_l_max        27
_diffrn_reflns_limit_l_min        -27
_diffrn_reflns_number             27200
_diffrn_reflns_point_group_measured_fraction_full  0.995
_diffrn_reflns_point_group_measured_fraction_max  0.995
```

```
_diffrn_reflns_theta_full          66.657
_diffrn_reflns_theta_max           66.657
_diffrn_reflns_theta_min           3.468
_diffrn_ambient_temperature        298(2)
_diffrn_detector_area_resol_mean   ?
_diffrn_measured_fraction_theta_full  0.995
_diffrn_measured_fraction_theta_max  0.995
_diffrn_measurement_device_type    'Bruker D8 Venture'
_diffrn_measurement_method         '\f and \w scans'
_diffrn_radiation_monochromator    'multilayer'
_diffrn_radiation_type             'CuK\a'
_diffrn_radiation_wavelength       1.54178
_diffrn_source                     'high brilliance microfocus
sealed tube'
_diffrn_source_current             1.0
_diffrn_source_power               0.05
_diffrn_source_voltage             50.0
_diffrn_standards_number           0
_reflns_Friedel_coverage           0.000
_reflns_Friedel_fraction_full      .
_reflns_Friedel_fraction_max       .
_reflns_number_gt                  3210
_reflns_number_total               4329
_reflns_special_details
;
 Reflections were merged by SHELXL according to the crystal
 class for the calculation of statistics and refinement.

 _reflns_Friedel_fraction is defined as the number of unique
 Friedel pairs measured divided by the number that would be
 possible theoretically, ignoring centric projections and
 systematic absences.
;
_reflns_threshold_expression       'I > 2\s(I)'
_computing_cell_refinement         'SAINT V8.40B (Bruker, 2016)'
_computing_data_collection         'Bruker Apex3, 2019'
_computing_data_reduction          'SAINT V8.40B (Bruker, 2016)'
_computing_molecular_graphics      'Olex2 1.3 (Dolomanov et al.,
2009)'
_computing_publication_material    'Olex2 1.3 (Dolomanov et al.,
2009)'
_computing_structure_refinement    'XL  (Sheldrick, 2008)'
_computing_structure_solution      'XT (Sheldrick, 2015)'
_refine_diff_density_max           0.363
_refine_diff_density_min           -0.263
_refine_diff_density_rms           0.035
_refine_ls_extinction_coef         0.0013(3)
_refine_ls_extinction_expression
 'Fc^*^=kFc[1+0.001xFc^2^\l^3^/sin(2\q)]^-1/4^'
_refine_ls_extinction_method       'SHELXL-2018/3 (Sheldrick 2018)'
_refine_ls_goodness_of_fit_ref     1.047
_refine_ls_hydrogen_treatment      constr
_refine_ls_matrix_type             full
_refine_ls_number_parameters       312
_refine_ls_number_reflns           4329
_refine_ls_number_restraints       16
_refine_ls_R_factor_all            0.0647
_refine_ls_R_factor_gt             0.0449
```

```
_refine_ls_restrained_S_all        1.048
_refine_ls_shift/su_max            0.001
_refine_ls_shift/su_mean           0.000
_refine_ls_structure_factor_coef   Fsqd
_refine_ls_weighting_details
 'w=1/[\s^2^(Fo^2^)+(0.0583P)^2^+0.7486P] where P=(Fo^2^+2Fc^2^)/3'
_refine_ls_weighting_scheme        calc
_refine_ls_wR_factor_gt            0.1136
_refine_ls_wR_factor_ref           0.1259
_refine_special_details            ?
_olex2_refinement_description
;
1. Fixed Uiso
 At 1.2 times of:
  All C(H) groups, All C(H,H) groups, All C(H,H,H,H) groups, All
N(H) groups
 At 1.5 times of:
  All C(H,H,H) groups, All C(H,H,H,H,H,H) groups
2. Restrained distances
 C30B-C29 \\sim C30A-C29
 with sigma of 0.02
 S31A-C30A \\sim S31B-C30B
 with sigma of 0.02
3. Rigid body (RIGU) restrains
 C29, S31B, C30B
 with sigma for 1-2 distances of 0.004 and sigma for 1-3 distances
of 0.004
 C29, S31A, C30A
 with sigma for 1-2 distances of 0.004 and sigma for 1-3 distances
of 0.004
4. Others

Sof(H29C)=Sof(H29D)=Sof(H32D)=Sof(H32E)=Sof(H32F)=Sof(S31B)=Sof(C30
B)=
 Sof(H30C)=Sof(H30D)=1-FVAR(1)

Sof(H29A)=Sof(H29B)=Sof(H32A)=Sof(H32B)=Sof(H32C)=Sof(S31A)=Sof(C30
A)=
 Sof(H30A)=Sof(H30B)=FVAR(1)
5.a Secondary CH2 refined with riding coordinates:
 C16(H16A,H16B), C17(H17A,H17B), C18(H18A,H18B), C28(H28A,H28B),
C29(H29C,
 H29D), C29(H29A,H29B), C30A(H30A,H30B), C30B(H30C,H30D)
5.b Aromatic/amide H refined with riding coordinates:
 N27(H27), C4(H4), C5(H5), C7(H7), C8(H8), C9(H9), C10(H10),
C12(H12), C13(H13)
5.c Idealised Me refined as rotating group:
 C1(H1A,H1B,H1C), C20(H20A,H20B,H20C), C32(H32D,H32E,H32F),
C32(H32A,H32B,H32C)
;
_atom_sites_solution_hydrogens     geom
_atom_sites_solution_primary       ?
_atom_sites_solution_secondary     ?
loop_
  _atom_site_label
  _atom_site_type_symbol
  _atom_site_fract_x
  _atom_site_fract_y
```

```
 _atom_site_fract_z
 _atom_site_U_iso_or_equiv
 _atom_site_adp_type
 _atom_site_occupancy
 _atom_site_site_symmetry_order
 _atom_site_calc_flag
 _atom_site_refinement_flags_posn
 _atom_site_refinement_flags_adp
 _atom_site_refinement_flags_occupancy
 _atom_site_disorder_assembly
 _atom_site_disorder_group
S19 S 0.73093(12) 0.40957(5) 0.65292(3) 0.0861(3) Uani 1 1 d . . .
. .
O2 O 0.4233(3) 0.82818(13) 0.70106(9) 0.0991(7) Uani 1 1 d . . . .
.
O24 O 0.5029(2) -0.02721(11) 0.41644(7) 0.0654(4) Uani 1 1 d . . .
. .
O26 O 0.72740(18) 0.14006(11) 0.46833(8) 0.0679(5) Uani 1 1 d . .
. . .
N15 N 0.4013(2) 0.31047(11) 0.51728(7) 0.0440(4) Uani 1 1 d . . .
. .
N27 N 0.0905(2) 0.06608(12) 0.43819(8) 0.0549(5) Uani 1 1 d . . .
. .
H27 H 0.001169 0.103350 0.445836 0.066 Uiso 1 1 calc R U . . .
C1 C 0.6163(5) 0.8507(2) 0.71390(14) 0.0932(9) Uani 1 1 d . . . .
.
H1A H 0.682687 0.863421 0.678952 0.140 Uiso 1 1 calc R U . . .
H1B H 0.678746 0.802475 0.733173 0.140 Uiso 1 1 calc R U . . .
H1C H 0.619189 0.901438 0.738331 0.140 Uiso 1 1 calc R U . . .
C3 C 0.3890(4) 0.75223(16) 0.67099(11) 0.0681(7) Uani 1 1 d . . .
. .
C4 C 0.1999(4) 0.7254(2) 0.66739(13) 0.0880(9) Uani 1 1 d . . . .
.
H4 H 0.104201 0.758876 0.684473 0.106 Uiso 1 1 calc R U . . .
C5 C 0.1510(4) 0.64941(18) 0.63866(12) 0.0771(8) Uani 1 1 d . . .
. .
H5 H 0.022398 0.631888 0.637133 0.092 Uiso 1 1 calc R U . . .
C6 C 0.2898(3) 0.59817(15) 0.61184(9) 0.0543(5) Uani 1 1 d . . . .
.
C7 C 0.4793(3) 0.62754(15) 0.61553(9) 0.0556(5) Uani 1 1 d . . . .
.
H7 H 0.575363 0.595254 0.597696 0.067 Uiso 1 1 calc R U . . .
C8 C 0.5296(3) 0.70310(15) 0.64480(9) 0.0592(6) Uani 1 1 d . . . .
.
H8 H 0.657966 0.720799 0.646831 0.071 Uiso 1 1 calc R U . . .
C9 C 0.2323(3) 0.51737(15) 0.58257(10) 0.0574(6) Uani 1 1 d . . .
. .
H9 H 0.100682 0.505371 0.581021 0.069 Uiso 1 1 calc R U . . .
C10 C 0.3471(3) 0.45911(14) 0.55790(9) 0.0515(5) Uani 1 1 d . . .
. .
H10 H 0.479132 0.470319 0.558549 0.062 Uiso 1 1 calc R U . . .
C11 C 0.2813(3) 0.37949(14) 0.53011(9) 0.0497(5) Uani 1 1 d . . .
. .
C12 C 0.0979(3) 0.35611(16) 0.51202(12) 0.0674(7) Uani 1 1 d . . .
. .
H12 H -0.012226 0.390633 0.515099 0.081 Uiso 1 1 calc R U . . .
C13 C 0.1051(3) 0.27299(16) 0.48859(11) 0.0630(6) Uani 1 1 d . . .
. .
```

H13 H 0.000858 0.241910 0.473305 0.076 Uiso 1 1 calc R U . . .
C14 C 0.2948(2) 0.24374(14) 0.49179(8) 0.0453(5) Uani 1 1 d . . .
. .
C16 C 0.6081(3) 0.30661(14) 0.53123(9) 0.0511(5) Uani 1 1 d . . .
. .
H16A H 0.657780 0.366036 0.533903 0.061 Uiso 1 1 calc R U . . .
H16B H 0.674073 0.277564 0.500048 0.061 Uiso 1 1 calc R U . . .
C17 C 0.6548(4) 0.25922(17) 0.58627(11) 0.0691(7) Uani 1 1 d . . .
. .
H17A H 0.588549 0.203106 0.585896 0.083 Uiso 1 1 calc R U . . .
H17B H 0.792416 0.247437 0.587761 0.083 Uiso 1 1 calc R U . . .
C18 C 0.6009(4) 0.30762(19) 0.63926(11) 0.0823(8) Uani 1 1 d . . .
. .
H18A H 0.621267 0.269000 0.671906 0.099 Uiso 1 1 calc R U . . .
H18B H 0.463998 0.320792 0.637014 0.099 Uiso 1 1 calc R U . . .
C20 C 0.9518(5) 0.3725(3) 0.68297(17) 0.1223(13) Uani 1 1 d . . .
. .
H20A H 1.023794 0.342073 0.654194 0.184 Uiso 1 1 calc R U . . .
H20B H 0.926460 0.333348 0.714300 0.184 Uiso 1 1 calc R U . . .
H20C H 1.025113 0.421831 0.696753 0.184 Uiso 1 1 calc R U . . .
C21 C 0.3689(2) 0.16318(13) 0.47027(8) 0.0427(5) Uani 1 1 d . . .
. .
C22 C 0.2715(3) 0.08931(13) 0.44717(8) 0.0437(5) Uani 1 1 d . . .
. .
C23 C 0.4558(3) 0.04290(15) 0.43615(8) 0.0471(5) Uani 1 1 d . . .
. .
C25 C 0.5570(3) 0.12179(14) 0.46059(9) 0.0478(5) Uani 1 1 d . . .
. .
C28 C 0.0341(3) -0.01965(16) 0.41594(11) 0.0636(6) Uani 1 1 d . .
. . .
H28A H -0.071194 -0.042145 0.438679 0.076 Uiso 1 1 calc R U . . .
H28B H 0.142204 -0.059683 0.420482 0.076 Uiso 1 1 calc R U . . .
C29 C -0.0270(4) -0.01847(18) 0.35472(12) 0.0788(8) Uani 1 1 d D .
. . .
H29C H 0.079286 0.000321 0.330983 0.095 Uiso 0.809(4) 1 calc R U P
A 2
H29D H -0.133283 0.022253 0.349147 0.095 Uiso 0.809(4) 1 calc R U
P A 2
H29A H 0.025318 0.035371 0.339039 0.095 Uiso 0.191(4) 1 calc R U P
A 1
H29B H -0.166054 -0.010959 0.354845 0.095 Uiso 0.191(4) 1 calc R U
P A 1
C32 C -0.4167(5) -0.0764(2) 0.27656(14) 0.1052(11) Uani 1 1 d . .
. . .
H32D H -0.485101 -0.072037 0.240542 0.158 Uiso 0.809(4) 1 calc R U
P A 2
H32E H -0.405140 -0.019103 0.293481 0.158 Uiso 0.809(4) 1 calc R U
P A 2
H32F H -0.486156 -0.114276 0.301926 0.158 Uiso 0.809(4) 1 calc R U
P A 2
H32A H -0.359692 -0.025749 0.259022 0.158 Uiso 0.191(4) 1 calc R U
P A 1
H32B H -0.504608 -0.057872 0.305676 0.158 Uiso 0.191(4) 1 calc R U
P A 1
H32C H -0.485328 -0.109829 0.247926 0.158 Uiso 0.191(4) 1 calc R U
P A 1
S31A S -0.2024(8) -0.1531(3) 0.3133(4) 0.121(3) Uani 0.191(4) 1 d
D . P A 1

```
 C30A C 0.014(3) -0.0930(13) 0.3075(10) 0.130(9) Uani 0.191(4) 1 d
D . P A 1
 H30A H 0.126061 -0.128160 0.317518 0.156 Uiso 0.191(4) 1 calc R U
P A 1
 H30B H 0.029001 -0.068318 0.269458 0.156 Uiso 0.191(4) 1 calc R U
P A 1
 S31B S -0.18792(16) -0.11930(9) 0.26522(6) 0.0932(6) Uani 0.809(4)
1 d D . P A
 2
 C30B C -0.0902(6) -0.1114(2) 0.33783(16) 0.0772(11) Uani 0.809(4)
1 d D . P A
 2
 H30C H -0.187244 -0.131322 0.364494 0.093 Uiso 0.809(4) 1 calc R U
P A 2
 H30D H 0.019920 -0.150458 0.341443 0.093 Uiso 0.809(4) 1 calc R U
P A 2

loop_
  _atom_site_aniso_label
  _atom_site_aniso_U_11
  _atom_site_aniso_U_22
  _atom_site_aniso_U_33
  _atom_site_aniso_U_23
  _atom_site_aniso_U_13
  _atom_site_aniso_U_12
 S19 0.1202(6) 0.0672(5) 0.0702(4) -0.0096(3) -0.0245(4) 0.0051(4)
 O2 0.1082(16) 0.0691(13) 0.1193(16) -0.0401(12) -0.0226(12)
0.0117(11)
 O24 0.0531(9) 0.0564(10) 0.0869(11) -0.0158(9) 0.0078(8) 0.0085(7)
 O26 0.0300(7) 0.0733(11) 0.1004(12) -0.0225(9) -0.0012(7)
0.0037(7)
 N15 0.0300(7) 0.0493(10) 0.0526(9) -0.0065(8) -0.0008(6) 0.0016(7)
 N27 0.0340(8) 0.0562(12) 0.0745(12) -0.0182(9) -0.0029(8)
0.0021(8)
 C1 0.113(2) 0.0663(19) 0.099(2) -0.0154(16) -0.0267(18) -
0.0081(17)
 C3 0.0832(17) 0.0515(15) 0.0694(15) -0.0073(12) -0.0099(13)
0.0068(12)
 C4 0.0753(18) 0.082(2) 0.106(2) -0.0398(17) 0.0032(15) 0.0187(15)
 C5 0.0578(14) 0.0750(18) 0.099(2) -0.0243(15) 0.0077(13)
0.0077(13)
 C6 0.0548(12) 0.0496(13) 0.0587(12) -0.0021(10) 0.0035(10)
0.0056(10)
 C7 0.0566(12) 0.0521(14) 0.0582(13) -0.0005(10) 0.0040(10)
0.0038(10)
 C8 0.0648(14) 0.0521(14) 0.0607(13) 0.0030(11) -0.0033(10) -
0.0007(11)
 C9 0.0439(11) 0.0551(14) 0.0734(14) -0.0050(11) 0.0042(10) -
0.0004(10)
 C10 0.0389(10) 0.0505(13) 0.0650(13) -0.0041(10) 0.0003(9)
0.0017(9)
 C11 0.0355(10) 0.0486(12) 0.0651(13) -0.0073(10) 0.0029(9)
0.0050(9)
 C12 0.0328(10) 0.0605(15) 0.1087(19) -0.0194(14) -0.0038(11)
0.0087(10)
 C13 0.0301(10) 0.0596(15) 0.0991(18) -0.0203(13) -0.0054(10)
0.0003(9)
```

```
 C14 0.0316(9) 0.0508(12) 0.0534(11) -0.0069(9) -0.0012(8)
0.0002(8)
 C16 0.0335(9) 0.0543(13) 0.0652(13) -0.0120(10) -0.0082(9)
0.0036(9)
 C17 0.0658(14) 0.0590(15) 0.0819(17) 0.0001(13) -0.0235(12)
0.0014(12)
 C18 0.097(2) 0.0790(19) 0.0711(16) 0.0113(14) 0.0008(14)
0.0026(16)
 C20 0.123(3) 0.119(3) 0.123(3) 0.028(2) -0.046(2) -0.005(2)
 C21 0.0302(9) 0.0532(12) 0.0448(10) -0.0026(9) -0.0004(7)
0.0029(8)
 C22 0.0354(9) 0.0507(12) 0.0450(10) -0.0036(9) -0.0003(8)
0.0030(8)
 C23 0.0382(10) 0.0537(14) 0.0496(11) -0.0018(10) 0.0032(8)
0.0063(9)
 C25 0.0336(10) 0.0562(13) 0.0535(11) -0.0044(10) -0.0005(8)
0.0051(9)
 C28 0.0448(11) 0.0554(15) 0.0904(17) -0.0125(12) -0.0062(11) -
0.0032(10)
 C29 0.0816(17) 0.0691(17) 0.0854(18) -0.0206(14) -0.0096(14) -
0.0057(14)
 C32 0.119(3) 0.107(3) 0.089(2) -0.0229(19) -0.0175(18) 0.014(2)
 S31A 0.115(4) 0.068(3) 0.181(9) -0.024(4) -0.011(4) -0.006(3)
 C30A 0.126(16) 0.125(16) 0.140(18) -0.043(13) 0.049(14) -0.018(14)
 S31B 0.1037(8) 0.0964(9) 0.0791(8) -0.0415(7) -0.0127(5) 0.0094(6)
 C30B 0.085(3) 0.054(2) 0.092(2) -0.0197(18) -0.030(2) 0.0025(18)

_geom_special_details
;
 All esds (except the esd in the dihedral angle between two l.s.
planes)
 are estimated using the full covariance matrix.  The cell esds are
taken
 into account individually in the estimation of esds in distances,
angles
 and torsion angles; correlations between esds in cell parameters
are only
 used when they are defined by crystal symmetry.  An approximate
(isotropic)
 treatment of cell esds is used for estimating esds involving l.s.
planes.
;
loop_
  _geom_bond_atom_site_label_1
  _geom_bond_atom_site_label_2
  _geom_bond_distance
  _geom_bond_site_symmetry_2
  _geom_bond_publ_flag
 S19 C18 1.821(3) . ?
 S19 C20 1.766(3) . ?
 O2 C1 1.408(3) . ?
 O2 C3 1.371(3) . ?
 O24 C23 1.209(2) . ?
 O26 C25 1.223(2) . ?
 N15 C11 1.375(2) . ?
 N15 C14 1.385(2) . ?
 N15 C16 1.465(2) . ?
 N27 H27 0.8600 . ?
```

N27 C22 1.317(2) . ?
N27 C28 1.456(3) . ?
C1 H1A 0.9600 . ?
C1 H1B 0.9600 . ?
C1 H1C 0.9600 . ?
C3 C4 1.373(4) . ?
C3 C8 1.377(3) . ?
C4 H4 0.9300 . ?
C4 C5 1.377(4) . ?
C5 H5 0.9300 . ?
C5 C6 1.393(3) . ?
C6 C7 1.388(3) . ?
C6 C9 1.460(3) . ?
C7 H7 0.9300 . ?
C7 C8 1.380(3) . ?
C8 H8 0.9300 . ?
C9 H9 0.9300 . ?
C9 C10 1.328(3) . ?
C10 H10 0.9300 . ?
C10 C11 1.445(3) . ?
C11 C12 1.380(3) . ?
C12 H12 0.9300 . ?
C12 C13 1.380(3) . ?
C13 H13 0.9300 . ?
C13 C14 1.389(3) . ?
C14 C21 1.424(3) . ?
C16 H16A 0.9700 . ?
C16 H16B 0.9700 . ?
C16 C17 1.502(3) . ?
C17 H17A 0.9700 . ?
C17 H17B 0.9700 . ?
C17 C18 1.489(4) . ?
C18 H18A 0.9700 . ?
C18 H18B 0.9700 . ?
C20 H20A 0.9600 . ?
C20 H20B 0.9600 . ?
C20 H20C 0.9600 . ?
C21 C22 1.414(3) . ?
C21 C25 1.468(3) . ?
C22 C23 1.485(3) . ?
C23 C25 1.499(3) . ?
C28 H28A 0.9700 . ?
C28 H28B 0.9700 . ?
C28 C29 1.482(4) . ?
C29 H29C 0.9700 . ?
C29 H29D 0.9700 . ?
C29 H29A 0.9700 . ?
C29 H29B 0.9700 . ?
C29 C30A 1.607(15) . ?
C29 C30B 1.531(4) . ?
C32 H32D 0.9600 . ?
C32 H32E 0.9600 . ?
C32 H32F 0.9600 . ?
C32 H32A 0.9600 . ?
C32 H32B 0.9600 . ?
C32 H32C 0.9600 . ?
C32 S31A 2.065(7) . ?
C32 S31B 1.738(4) . ?

```
 S31A C30A 1.762(15) . ?
 C30A H30A 0.9700 . ?
 C30A H30B 0.9700 . ?
 S31B C30B 1.816(3) . ?
 C30B H30C 0.9700 . ?
 C30B H30D 0.9700 . ?

loop_
  _geom_angle_atom_site_label_1
  _geom_angle_atom_site_label_2
  _geom_angle_atom_site_label_3
  _geom_angle
  _geom_angle_site_symmetry_1
  _geom_angle_site_symmetry_3
  _geom_angle_publ_flag
 C20 S19 C18 102.71(16) . . ?
 C3 O2 C1 118.1(2) . . ?
 C11 N15 C14 109.52(15) . . ?
 C11 N15 C16 125.05(16) . . ?
 C14 N15 C16 125.36(16) . . ?
 C22 N27 H27 118.4 . . ?
 C22 N27 C28 123.19(18) . . ?
 C28 N27 H27 118.4 . . ?
 O2 C1 H1A 109.5 . . ?
 O2 C1 H1B 109.5 . . ?
 O2 C1 H1C 109.5 . . ?
 H1A C1 H1B 109.5 . . ?
 H1A C1 H1C 109.5 . . ?
 H1B C1 H1C 109.5 . . ?
 O2 C3 C4 116.1(2) . . ?
 O2 C3 C8 124.4(2) . . ?
 C4 C3 C8 119.4(2) . . ?
 C3 C4 H4 119.7 . . ?
 C3 C4 C5 120.5(2) . . ?
 C5 C4 H4 119.7 . . ?
 C4 C5 H5 119.3 . . ?
 C4 C5 C6 121.5(2) . . ?
 C6 C5 H5 119.3 . . ?
 C5 C6 C9 119.7(2) . . ?
 C7 C6 C5 116.7(2) . . ?
 C7 C6 C9 123.52(19) . . ?
 C6 C7 H7 119.0 . . ?
 C8 C7 C6 122.1(2) . . ?
 C8 C7 H7 119.0 . . ?
 C3 C8 C7 119.8(2) . . ?
 C3 C8 H8 120.1 . . ?
 C7 C8 H8 120.1 . . ?
 C6 C9 H9 116.4 . . ?
 C10 C9 C6 127.2(2) . . ?
 C10 C9 H9 116.4 . . ?
 C9 C10 H10 117.7 . . ?
 C9 C10 C11 124.56(19) . . ?
 C11 C10 H10 117.7 . . ?
 N15 C11 C10 123.41(17) . . ?
 N15 C11 C12 107.05(18) . . ?
 C12 C11 C10 129.54(19) . . ?
 C11 C12 H12 125.7 . . ?
 C13 C12 C11 108.62(19) . . ?
```

C13 C12 H12 125.7 . . ?
C12 C13 H13 125.9 . . ?
C12 C13 C14 108.21(19) . . ?
C14 C13 H13 125.9 . . ?
N15 C14 C13 106.60(17) . . ?
N15 C14 C21 126.24(16) . . ?
C13 C14 C21 127.07(18) . . ?
N15 C16 H16A 108.8 . . ?
N15 C16 H16B 108.8 . . ?
N15 C16 C17 114.00(18) . . ?
H16A C16 H16B 107.6 . . ?
C17 C16 H16A 108.8 . . ?
C17 C16 H16B 108.8 . . ?
C16 C17 H17A 108.6 . . ?
C16 C17 H17B 108.6 . . ?
H17A C17 H17B 107.6 . . ?
C18 C17 C16 114.6(2) . . ?
C18 C17 H17A 108.6 . . ?
C18 C17 H17B 108.6 . . ?
S19 C18 H18A 108.3 . . ?
S19 C18 H18B 108.3 . . ?
C17 C18 S19 116.0(2) . . ?
C17 C18 H18A 108.3 . . ?
C17 C18 H18B 108.3 . . ?
H18A C18 H18B 107.4 . . ?
S19 C20 H20A 109.5 . . ?
S19 C20 H20B 109.5 . . ?
S19 C20 H20C 109.5 . . ?
H20A C20 H20B 109.5 . . ?
H20A C20 H20C 109.5 . . ?
H20B C20 H20C 109.5 . . ?
C14 C21 C25 138.51(18) . . ?
C22 C21 C14 130.35(16) . . ?
C22 C21 C25 91.13(16) . . ?
N27 C22 C21 136.24(18) . . ?
N27 C22 C23 131.60(19) . . ?
C21 C22 C23 92.16(15) . . ?
O24 C23 C22 136.32(19) . . ?
O24 C23 C25 136.46(18) . . ?
C22 C23 C25 87.22(15) . . ?
O26 C25 C21 137.4(2) . . ?
O26 C25 C23 133.09(19) . . ?
C21 C25 C23 89.49(15) . . ?
N27 C28 H28A 108.8 . . ?
N27 C28 H28B 108.8 . . ?
N27 C28 C29 113.8(2) . . ?
H28A C28 H28B 107.7 . . ?
C29 C28 H28A 108.8 . . ?
C29 C28 H28B 108.8 . . ?
C28 C29 H29C 110.1 . . ?
C28 C29 H29D 110.1 . . ?
C28 C29 H29A 105.6 . . ?
C28 C29 H29B 105.6 . . ?
C28 C29 C30A 126.8(11) . . ?
C28 C29 C30B 108.2(2) . . ?
H29C C29 H29D 108.4 . . ?
H29A C29 H29B 106.1 . . ?
C30A C29 H29A 105.6 . . ?

```
 C30A C29 H29B 105.6 . . ?
 C30B C29 H29C 110.1 . . ?
 C30B C29 H29D 110.1 . . ?
 H32D C32 H32E 109.5 . . ?
 H32D C32 H32F 109.5 . . ?
 H32E C32 H32F 109.5 . . ?
 H32A C32 H32B 109.5 . . ?
 H32A C32 H32C 109.5 . . ?
 H32B C32 H32C 109.5 . . ?
 S31A C32 H32A 109.5 . . ?
 S31A C32 H32B 109.5 . . ?
 S31A C32 H32C 109.5 . . ?
 S31B C32 H32D 109.5 . . ?
 S31B C32 H32E 109.5 . . ?
 S31B C32 H32F 109.5 . . ?
 C30A S31A C32 106.3(10) . . ?
 C29 C30A S31A 99.1(9) . . ?
 C29 C30A H30A 112.0 . . ?
 C29 C30A H30B 112.0 . . ?
 S31A C30A H30A 112.0 . . ?
 S31A C30A H30B 112.0 . . ?
 H30A C30A H30B 109.6 . . ?
 C32 S31B C30B 99.35(18) . . ?
 C29 C30B S31B 113.7(2) . . ?
 C29 C30B H30C 108.8 . . ?
 C29 C30B H30D 108.8 . . ?
 S31B C30B H30C 108.8 . . ?
 S31B C30B H30D 108.8 . . ?
 H30C C30B H30D 107.7 . . ?

loop_
  _geom_hbond_atom_site_label_D
  _geom_hbond_atom_site_label_H
  _geom_hbond_atom_site_label_A
  _geom_hbond_distance_DH
  _geom_hbond_distance_HA
  _geom_hbond_distance_DA
  _geom_hbond_angle_DHA
  _geom_hbond_site_symmetry_A
  _geom_hbond_publ_flag
 N27 H27 O26 0.86 2.05 2.852(2) 154.5 1_455 yes
 C16 H16B O26 0.97 2.25 3.049(3) 138.6 . yes

loop_
  _geom_torsion_atom_site_label_1
  _geom_torsion_atom_site_label_2
  _geom_torsion_atom_site_label_3
  _geom_torsion_atom_site_label_4
  _geom_torsion
  _geom_torsion_site_symmetry_1
  _geom_torsion_site_symmetry_2
  _geom_torsion_site_symmetry_3
  _geom_torsion_site_symmetry_4
  _geom_torsion_publ_flag
 O2 C3 C4 C5 -179.3(3) . . . . ?
 O2 C3 C8 C7 -180.0(2) . . . . ?
 O24 C23 C25 O26 0.1(4) . . . . ?
 O24 C23 C25 C21 -179.7(3) . . . . ?
```

N15 C11 C12 C13 0.4(3) . . . . ?
N15 C14 C21 C22 175.36(19) . . . . ?
N15 C14 C21 C25 -6.8(4) . . . . ?
N15 C16 C17 C18 -71.7(3) . . . . ?
N27 C22 C23 O24 0.2(4) . . . . ?
N27 C22 C23 C25 -179.6(2) . . . . ?
N27 C28 C29 C30A 143.1(7) . . . . ?
N27 C28 C29 C30B -177.6(2) . . . . ?
C1 O2 C3 C4 169.9(3) . . . . ?
C1 O2 C3 C8 -10.4(4) . . . . ?
C3 C4 C5 C6 -0.9(5) . . . . ?
C4 C3 C8 C7 -0.3(4) . . . . ?
C4 C5 C6 C7 0.0(4) . . . . ?
C4 C5 C6 C9 179.1(3) . . . . ?
C5 C6 C7 C8 0.8(3) . . . . ?
C5 C6 C9 C10 -175.9(2) . . . . ?
C6 C7 C8 C3 -0.6(3) . . . . ?
C6 C9 C10 C11 179.2(2) . . . . ?
C7 C6 C9 C10 3.1(4) . . . . ?
C8 C3 C4 C5 1.0(4) . . . . ?
C9 C6 C7 C8 -178.3(2) . . . . ?
C9 C10 C11 N15 -163.7(2) . . . . ?
C9 C10 C11 C12 15.7(4) . . . . ?
C10 C11 C12 C13 -179.1(2) . . . . ?
C11 N15 C14 C13 0.4(2) . . . . ?
C11 N15 C14 C21 177.22(19) . . . . ?
C11 N15 C16 C17 95.5(2) . . . . ?
C11 C12 C13 C14 -0.2(3) . . . . ?
C12 C13 C14 N15 -0.2(3) . . . . ?
C12 C13 C14 C21 -176.9(2) . . . . ?
C13 C14 C21 C22 -8.5(4) . . . . ?
C13 C14 C21 C25 169.4(2) . . . . ?
C14 N15 C11 C10 179.0(2) . . . . ?
C14 N15 C11 C12 -0.5(2) . . . . ?
C14 N15 C16 C17 -81.1(2) . . . . ?
C14 C21 C22 N27 -1.8(4) . . . . ?
C14 C21 C22 C23 178.8(2) . . . . ?
C14 C21 C25 O26 1.7(5) . . . . ?
C14 C21 C25 C23 -178.5(2) . . . . ?
C16 N15 C11 C10 2.0(3) . . . . ?
C16 N15 C11 C12 -177.6(2) . . . . ?
C16 N15 C14 C13 177.48(19) . . . . ?
C16 N15 C14 C21 -5.7(3) . . . . ?
C16 C17 C18 S19 -65.3(3) . . . . ?
C20 S19 C18 C17 -80.0(2) . . . . ?
C21 C22 C23 O24 179.7(3) . . . . ?
C21 C22 C23 C25 -0.16(15) . . . . ?
C22 N27 C28 C29 -104.2(3) . . . . ?
C22 C21 C25 O26 -179.9(3) . . . . ?
C22 C21 C25 C23 -0.16(15) . . . . ?
C22 C23 C25 O26 179.9(3) . . . . ?
C22 C23 C25 C21 0.16(15) . . . . ?
C25 C21 C22 N27 179.6(2) . . . . ?
C25 C21 C22 C23 0.16(15) . . . . ?
C28 N27 C22 C21 -176.1(2) . . . . ?
C28 N27 C22 C23 3.1(3) . . . . ?
C28 C29 C30A S31A 94.2(12) . . . . ?
C28 C29 C30B S31B 175.4(2) . . . . ?

```
C32 S31A C30A C29 72.0(14) . . . . ?
C32 S31B C30B C29 -77.6(3) . . . . ?
```

Table S5. Final structures obtained from the DFT calculations in pdb format.

****

TITLE   *cis*-Pt-MeR"S"'

REMARK   1 File created by GaussView 6.0.16

```
HETATM    1  C     0    -2.314   2.556  -0.518      C
HETATM    2  C     0    -1.203   3.300  -0.071      C
HETATM    3  C     0    -2.058   4.423   0.406      C
HETATM    4  C     0    -3.290   3.581   0.013      C
HETATM    5  O     0    -1.856   5.541   0.879      O
HETATM    6  O     0    -4.517   3.644   0.106      O
HETATM    7  N     0    -2.581   1.408  -1.172      N
HETATM    8  H     0    -1.851   0.811  -1.560      H
HETATM    9  C     0    -3.961   1.090  -1.597      C
HETATM   10  H     0    -3.885   0.416  -2.452      H
HETATM   11  H     0    -4.445   2.014  -1.925      H
HETATM   12  C     0    -4.607  -1.016  -0.178      C
HETATM   13  H     0    -4.402  -1.605  -1.074      H
HETATM   14  H     0    -5.467  -1.432   0.353      H
HETATM   15  S     0    -3.155  -1.432   0.985      S
HETATM   16  C     0     1.077   4.383  -0.147      C
HETATM   17  C     0     2.406   3.907  -0.268      C
HETATM   18  H     0     0.738   5.408  -0.131      H
HETATM   19  C     0     2.361   2.505  -0.264      C
HETATM   20  H     0     3.297   4.504  -0.384      H
HETATM   21  C     0     0.228   3.267  -0.101      C
HETATM   22  N     0     1.028   2.125  -0.162      N
HETATM   23  C     0     0.608   0.765   0.178      C
HETATM   24  H     0    -0.482   0.716   0.104      H
HETATM   25  H     0     1.007   0.060  -0.558      H
HETATM   26  C     0     1.066   0.379   1.612      C
HETATM   27  H     0     1.915   1.022   1.875      H
HETATM   28  H     0     0.275   0.614   2.338      H
HETATM   29  C     0     1.565  -1.059   1.776      C
HETATM   30  H     0     2.235  -1.352   0.964      H
```

```
HETATM   31  H          0      2.078  -1.191   2.732                    H
HETATM   32  S          0      0.244  -2.416   1.759                    S
HETATM   33  C          0      3.417   1.507  -0.347                    C
HETATM   34  H          0      3.111   0.505  -0.645                    H
HETATM   35  C          0      4.726   1.752  -0.101                    C
HETATM   36  H          0      5.015   2.750   0.228                    H
HETATM   37  C          0      5.819   0.772  -0.214                    C
HETATM   38  C          0      7.106   1.138   0.230                    C
HETATM   39  C          0      5.635  -0.525  -0.745                    C
HETATM   40  C          0      8.178   0.239   0.161                    C
HETATM   41  H          0      7.264   2.136   0.634                    H
HETATM   42  C          0      6.704  -1.425  -0.812                    C
HETATM   43  H          0      4.665  -0.832  -1.125                    H
HETATM   44  C          0      7.980  -1.049  -0.358                    C
HETATM   45  H          0      9.161   0.540   0.508                    H
HETATM   46  H          0      6.546  -2.415  -1.226                    H
HETATM   47  H          0      8.807  -1.749  -0.416                    H
HETATM   48 Pt          0     -1.083  -1.924  -0.210                    Pt
HETATM   49  C          0     -4.846   0.462  -0.508                    C
HETATM   50  H          0     -5.879   0.513  -0.881                    H
HETATM   51  H          0     -4.836   1.097   0.385                    H
HETATM   52 Cl          0     -2.129  -1.587  -2.342                    Cl
HETATM   53 Cl          0      0.998  -2.446  -1.308                    Cl
HETATM   54  C          0     -0.627  -2.075   3.380                    C
HETATM   55  H          0     -1.668  -2.388   3.280                    H
HETATM   56  H          0     -0.132  -2.672   4.145                    H
HETATM   57  H          0     -0.564  -1.017   3.635                    H
HETATM   58  C          0     -2.928   0.155   1.943                    C
HETATM   59  H          0     -2.618   0.960   1.280                    H
HETATM   60  H          0     -3.871   0.398   2.435                    H
HETATM   61  H          0     -2.165  -0.035   2.696                    H
END
CONECT    1    2    4    7
CONECT    2    1    3   21
CONECT    3    2    4    5
CONECT    4    1    3    6
CONECT    5    3
CONECT    6    4
CONECT    7    1    8    9
CONECT    8    7
```

```
CONECT    9    7   10   11   49
CONECT   10    9
CONECT   11    9
CONECT   12   13   14   49
CONECT   13   12
CONECT   14   12
CONECT   15   58
CONECT   16   17   18   21
CONECT   17   16   19   20
CONECT   18   16
CONECT   19   17   22   33
CONECT   20   17
CONECT   21    2   16   22
CONECT   22   19   21   23
CONECT   23   22   24   25   26
CONECT   24   23
CONECT   25   23
CONECT   26   23   27   28   29
CONECT   27   26
CONECT   28   26
CONECT   29   26   30   31
CONECT   30   29
CONECT   31   29
CONECT   32   48   54
CONECT   33   19   34   35
CONECT   34   33
CONECT   35   33   36   37
CONECT   36   35
CONECT   37   35   38   39
CONECT   38   37   40   41
CONECT   39   37   42   43
CONECT   40   38   44   45
CONECT   41   38
CONECT   42   39   44   46
CONECT   43   39
CONECT   44   40   42   47
CONECT   45   40
CONECT   46   42
CONECT   47   44
CONECT   48   32   52   53
```

```
CONECT   49   12    9   50   51
CONECT   50   49
CONECT   51   49
CONECT   52   48
CONECT   53   48
CONECT   54   32   55   56   57
CONECT   55   54
CONECT   56   54
CONECT   57   54
CONECT   58   15   59   60   61
CONECT   59   58
CONECT   60   58
CONECT   61   58
*****************************************
```

TITLE *cis*-Pt-MeR"R'"

```
REMARK   1 File created by GaussView 6.0.16
HETATM    1  C     0    -0.643   2.841  -0.547    C
HETATM    2  C     0     0.677   3.250  -0.253    C
HETATM    3  C     0     0.236   4.635   0.088    C
HETATM    4  C     0    -1.207   4.195  -0.189    C
HETATM    5  O     0     0.798   5.677   0.430    O
HETATM    6  O     0    -2.339   4.683  -0.143    O
HETATM    7  N     0    -1.324   1.763  -0.979    N
HETATM    8  H     0    -0.866   0.901  -1.270    H
HETATM    9  C     0    -2.794   1.813  -1.121    C
HETATM   10  H     0    -3.087   0.930  -1.692    H
HETATM   11  H     0    -3.064   2.696  -1.708    H
HETATM   12  C     0    -4.763   0.936   0.324    C
HETATM   13  H     0    -5.142   0.658  -0.663    H
HETATM   14  H     0    -5.562   1.366   0.931    H
HETATM   15  S     0    -4.310  -0.686   1.195    S
HETATM   16  C     0     3.134   3.681  -0.053    C
HETATM   17  C     0     4.309   2.900  -0.082    C
HETATM   18  H     0     3.048   4.750   0.065    H
HETATM   19  C     0     3.933   1.560  -0.264    C
HETATM   20  H     0     5.326   3.255  -0.024    H
HETATM   21  C     0     2.040   2.816  -0.235    C
HETATM   22  N     0     2.545   1.519  -0.364    N
HETATM   23  C     0     1.735   0.302  -0.358    C
HETATM   24  H     0     1.075   0.278  -1.228    H
```

```
HETATM   25  H          0       2.407  -0.552  -0.470                      H
HETATM   26  C          0       0.937   0.155   0.963                      C
HETATM   27  H          0       1.487   0.680   1.752                      H
HETATM   28  H          0      -0.027   0.666   0.853                      H
HETATM   29  C          0       0.733  -1.317   1.365                      C
HETATM   30  H          0       0.952  -2.003   0.546                      H
HETATM   31  H          0       1.330  -1.590   2.239                      H
HETATM   32  S          0      -1.040  -1.763   1.831                      S
HETATM   33  C          0       4.782   0.380  -0.370                      C
HETATM   34  H          0       4.397  -0.471  -0.928                      H
HETATM   35  C          0       6.027   0.317   0.159                      C
HETATM   36  H          0       6.377   1.159   0.757                      H
HETATM   37  C          0       6.972  -0.805   0.026                      C
HETATM   38  C          0       8.134  -0.811   0.825                      C
HETATM   39  C          0       6.768  -1.879  -0.869                      C
HETATM   40  C          0       9.057  -1.862   0.750                      C
HETATM   41  H          0       8.308   0.013   1.512                      H
HETATM   42  C          0       7.688  -2.930  -0.944                      C
HETATM   43  H          0       5.901  -1.886  -1.523                      H
HETATM   44  C          0       8.836  -2.928  -0.133                      C
HETATM   45  H          0       9.944  -1.848   1.376                      H
HETATM   46  H          0       7.519  -3.745  -1.640                      H
HETATM   47  H          0       9.550  -3.743  -0.197                      H
HETATM   48 Pt          0      -2.444  -1.435  -0.140                      Pt
HETATM   49  C          0      -3.541   1.869   0.239                      C
HETATM   50  H          0      -3.868   2.898   0.415                      H
HETATM   51  H          0      -2.832   1.639   1.047                      H
HETATM   52 Cl          0      -3.904  -1.426  -2.048                      Cl
HETATM   53 Cl          0      -0.556  -1.864  -1.568                      Cl
HETATM   54  C          0      -1.409  -0.422   3.079                      C
HETATM   55  H          0      -2.425  -0.582   3.436                      H
HETATM   56  H          0      -0.699  -0.526   3.901                      H
HETATM   57  H          0      -1.323   0.564   2.621                      H
HETATM   58  C          0      -5.772  -1.765   0.750                      C
HETATM   59  H          0      -5.578  -2.752   1.170                      H
HETATM   60  H          0      -6.664  -1.326   1.200                      H
HETATM   61  H          0      -5.841  -1.821  -0.336                      H
END
CONECT    1    2    4    7
CONECT    2    1    3   21
```

```
CONECT    3    2    4    5
CONECT    4    1    3    6
CONECT    5    3
CONECT    6    4
CONECT    7    1    8    9
CONECT    8    7
CONECT    9    7   10   11   49
CONECT   10    9
CONECT   11    9
CONECT   12   13   14   49
CONECT   13   12
CONECT   14   12
CONECT   15   48   58
CONECT   16   17   18   21
CONECT   17   16   19   20
CONECT   18   16
CONECT   19   17   22   33
CONECT   20   17
CONECT   21    2   16   22
CONECT   22   21   19   23
CONECT   23   22   24   25   26
CONECT   24   23
CONECT   25   23
CONECT   26   23   27   28   29
CONECT   27   26
CONECT   28   26
CONECT   29   26   30   31
CONECT   30   29
CONECT   31   29
CONECT   32   54
CONECT   33   19   34   35
CONECT   34   33
CONECT   35   33   36   37
CONECT   36   35
CONECT   37   35   38   39
CONECT   38   37   40   41
CONECT   39   37   42   43
CONECT   40   38   44   45
CONECT   41   38
CONECT   42   39   44   46
```

```
CONECT   43   39
CONECT   44   42   40   47
CONECT   45   40
CONECT   46   42
CONECT   47   44
CONECT   48   15   52   53
CONECT   49   12    9   50   51
CONECT   50   49
CONECT   51   49
CONECT   52   48
CONECT   53   48
CONECT   54   32   55   56   57
CONECT   55   54
CONECT   56   54
CONECT   57   54
CONECT   58   15   59   60   61
CONECT   59   58
CONECT   60   58
CONECT   61   58
```

****

TITLE *cis*-Pt-MeS"S'"

```
REMARK   1 File created by GaussView 6.0.16
HETATM    1  C     0    -0.746   2.939  -0.480     C
HETATM    2  C     0     0.567   3.368  -0.185     C
HETATM    3  C     0     0.102   4.731   0.204     C
HETATM    4  C     0    -1.336   4.268  -0.069     C
HETATM    5  O     0     0.646   5.772   0.574     O
HETATM    6  O     0    -2.477   4.726   0.013     O
HETATM    7  N     0    -1.405   1.861  -0.944     N
HETATM    8  H     0    -0.931   1.021  -1.265     H
HETATM    9  C     0    -2.880   1.860  -1.020     C
HETATM   10  H     0    -3.167   0.993  -1.617     H
HETATM   11  H     0    -3.212   2.758  -1.549     H
HETATM   12  C     0    -4.790   0.920   0.448     C
HETATM   13  H     0    -5.258   0.775  -0.529     H
HETATM   14  H     0    -5.535   1.299   1.153     H
HETATM   15  S     0    -4.443  -0.848   1.049     S
HETATM   16  C     0     3.020   3.815   0.047     C
HETATM   17  C     0     4.200   3.040   0.006     C
```

HETATM	18	H	0	2.926	4.879	0.202	H
HETATM	19	C	0	3.834	1.707	-0.229	C
HETATM	20	H	0	5.215	3.398	0.090	H
HETATM	21	C	0	1.935	2.951	-0.179	C
HETATM	22	N	0	2.448	1.663	-0.352	N
HETATM	23	C	0	1.646	0.443	-0.398	C
HETATM	24	H	0	0.946	0.483	-1.236	H
HETATM	25	H	0	2.314	-0.392	-0.616	H
HETATM	26	C	0	0.920	0.179	0.946	C
HETATM	27	H	0	1.514	0.627	1.748	H
HETATM	28	H	0	-0.051	0.692	0.948	H
HETATM	29	C	0	0.752	-1.329	1.207	C
HETATM	30	H	0	0.965	-1.920	0.315	H
HETATM	31	H	0	1.367	-1.668	2.043	H
HETATM	32	S	0	-1.017	-1.781	1.690	S
HETATM	33	C	0	4.690	0.534	-0.358	C
HETATM	34	H	0	4.325	-0.296	-0.960	H
HETATM	35	C	0	5.920	0.453	0.204	C
HETATM	36	H	0	6.247	1.270	0.847	H
HETATM	37	C	0	6.871	-0.661	0.054	C
HETATM	38	C	0	7.998	-0.709	0.902	C
HETATM	39	C	0	6.709	-1.686	-0.905	C
HETATM	40	C	0	8.925	-1.755	0.813	C
HETATM	41	H	0	8.140	0.079	1.637	H
HETATM	42	C	0	7.634	-2.732	-0.994	C
HETATM	43	H	0	5.874	-1.655	-1.597	H
HETATM	44	C	0	8.745	-2.774	-0.134	C
HETATM	45	H	0	9.784	-1.773	1.476	H
HETATM	46	H	0	7.497	-3.509	-1.739	H
HETATM	47	H	0	9.463	-3.584	-0.209	H
HETATM	48	Pt	0	-2.474	-1.424	-0.233	Pt
HETATM	49	C	0	-3.546	1.819	0.379	C
HETATM	50	H	0	-3.830	2.839	0.661	H
HETATM	51	H	0	-2.795	1.497	1.113	H
HETATM	52	Cl	0	-3.999	-1.340	-2.083	Cl
HETATM	53	Cl	0	-0.605	-1.815	-1.697	Cl
HETATM	54	C	0	-0.859	-3.647	1.589	C
HETATM	55	H	0	-0.540	-3.906	0.578	H
HETATM	56	H	0	-0.138	-3.976	2.338	H
HETATM	57	H	0	-1.843	-4.071	1.792	H

```
HETATM   58  C          0      -3.936  -0.477   2.809                   C
HETATM   59  H          0      -3.100   0.222   2.836                   H
HETATM   60  H          0      -4.804  -0.065   3.325                   H
HETATM   61  H          0      -3.640  -1.419   3.271                   H
END
CONECT    1    2    4    7
CONECT    2    1    3   21
CONECT    3    2    4    5
CONECT    4    1    3    6
CONECT    5    3
CONECT    6    4
CONECT    7    1    8    9
CONECT    8    7
CONECT    9    7   10   11   49
CONECT   10    9
CONECT   11    9
CONECT   12   13   14   49
CONECT   13   12
CONECT   14   12
CONECT   15   48   58
CONECT   16   17   18   21
CONECT   17   16   19   20
CONECT   18   16
CONECT   19   17   22   33
CONECT   20   17
CONECT   21    2   16   22
CONECT   22   21   19   23
CONECT   23   22   24   25   26
CONECT   24   23
CONECT   25   23
CONECT   26   23   27   28   29
CONECT   27   26
CONECT   28   26
CONECT   29   26   30   31
CONECT   30   29
CONECT   31   29
CONECT   32   54
CONECT   33   19   34   35
CONECT   34   33
CONECT   35   33   36   37
```

CONECT 36 35
CONECT 37 35 38 39
CONECT 38 37 40 41
CONECT 39 37 42 43
CONECT 40 38 44 45
CONECT 41 38
CONECT 42 39 44 46
CONECT 43 39
CONECT 44 40 42 47
CONECT 45 40
CONECT 46 42
CONECT 47 44
CONECT 48 15 52 53
CONECT 49 9 12 50 51
CONECT 50 49
CONECT 51 49
CONECT 52 48
CONECT 53 48
CONECT 54 32 55 56 57
CONECT 55 54
CONECT 56 54
CONECT 57 54
CONECT 58 15 59 60 61
CONECT 59 58
CONECT 60 58
CONECT 61 58

****

TITLE *cis*-Pt-MeS"R'"
REMARK 1 File created by GaussView 6.0.16
HETATM 1 C 0 0.648 2.982 0.393 C
HETATM 2 C 0 -0.678 3.385 0.128 C
HETATM 3 C 0 -0.251 4.765 -0.243 C
HETATM 4 C 0 1.202 4.331 0.002 C
HETATM 5 O 0 -0.825 5.801 -0.582 O
HETATM 6 O 0 2.331 4.817 -0.079 O
HETATM 7 N 0 1.337 1.911 0.829 N
HETATM 8 H 0 0.883 1.052 1.127 H
HETATM 9 C 0 2.808 1.959 0.941 C
HETATM 10 H 0 3.108 1.096 1.538 H

HETATM	11	H	0	3.093	2.861	1.490	H
HETATM	12	C	0	4.753	1.035	-0.478	C
HETATM	13	H	0	5.160	0.836	0.518	H
HETATM	14	H	0	5.535	1.424	-1.135	H
HETATM	15	S	0	4.289	-0.645	-1.218	S
HETATM	16	C	0	-3.147	3.776	-0.024	C
HETATM	17	C	0	-4.307	2.973	0.041	C
HETATM	18	H	0	-3.082	4.845	-0.161	H
HETATM	19	C	0	-3.902	1.644	0.236	C
HETATM	20	H	0	-5.332	3.308	-0.002	H
HETATM	21	C	0	-2.035	2.934	0.148	C
HETATM	22	N	0	-2.511	1.631	0.312	N
HETATM	23	C	0	-1.680	0.428	0.291	C
HETATM	24	H	0	-0.953	0.455	1.106	H
HETATM	25	H	0	-2.321	-0.430	0.503	H
HETATM	26	C	0	-1.005	0.232	-1.089	C
HETATM	27	H	0	-1.676	0.640	-1.851	H
HETATM	28	H	0	-0.082	0.825	-1.140	H
HETATM	29	C	0	-0.735	-1.253	-1.391	C
HETATM	30	H	0	-0.949	-1.891	-0.532	H
HETATM	31	H	0	-1.297	-1.595	-2.263	H
HETATM	32	S	0	1.070	-1.578	-1.833	S
HETATM	33	C	0	-4.725	0.448	0.369	C
HETATM	34	H	0	-4.318	-0.387	0.935	H
HETATM	35	C	0	-5.974	0.352	-0.148	C
HETATM	36	H	0	-6.344	1.178	-0.755	H
HETATM	37	C	0	-6.895	-0.786	0.008	C
HETATM	38	C	0	-8.061	-0.826	-0.785	C
HETATM	39	C	0	-6.665	-1.842	0.917	C
HETATM	40	C	0	-8.963	-1.894	-0.689	C
HETATM	41	H	0	-8.255	-0.016	-1.482	H
HETATM	42	C	0	-7.563	-2.910	1.013	C
HETATM	43	H	0	-5.795	-1.821	1.567	H
HETATM	44	C	0	-8.716	-2.943	0.209	C
HETATM	45	H	0	-9.853	-1.907	-1.309	H
HETATM	46	H	0	-7.374	-3.711	1.720	H
HETATM	47	H	0	-9.413	-3.771	0.289	H
HETATM	48	Pt	0	2.438	-1.344	0.170	Pt
HETATM	49	C	0	3.525	1.959	-0.435	C
HETATM	50	H	0	3.836	2.981	-0.669	H

```
HETATM   51  H          0       2.808   1.673  -1.217                    H
HETATM   52 Cl          0       3.895  -1.270   2.076                   Cl
HETATM   53 Cl          0       0.547  -1.892   1.548                   Cl
HETATM   54  C          0       0.990  -3.448  -1.929                    C
HETATM   55  H          0       0.643  -3.829  -0.966                    H
HETATM   56  H          0       0.313  -3.725  -2.738                    H
HETATM   57  H          0       1.997  -3.808  -2.137                    H
HETATM   58  C          0       5.764  -1.675  -0.704                    C
HETATM   59  H          0       5.581  -2.693  -1.046                    H
HETATM   60  H          0       6.647  -1.262  -1.195                    H
HETATM   61  H          0       5.847  -1.650   0.383                    H
END
CONECT    1    2    4    7
CONECT    2    1    3   21
CONECT    3    2    4    5
CONECT    4    3    1    6
CONECT    5    3
CONECT    6    4
CONECT    7    1    8    9
CONECT    8    7
CONECT    9    7   10   11   49
CONECT   10    9
CONECT   11    9
CONECT   12   13   14   49
CONECT   13   12
CONECT   14   12
CONECT   15   48   58
CONECT   16   17   18   21
CONECT   17   16   19   20
CONECT   18   16
CONECT   19   17   22   33
CONECT   20   17
CONECT   21   16    2   22
CONECT   22   19   21   23
CONECT   23   22   24   25   26
CONECT   24   23
CONECT   25   23
CONECT   26   23   27   28   29
CONECT   27   26
CONECT   28   26
```

```
CONECT   29   26   30   31   32
CONECT   30   29
CONECT   31   29
CONECT   32   29   48   54
CONECT   33   19   34   35
CONECT   34   33
CONECT   35   33   36   37
CONECT   36   35
CONECT   37   35   38   39
CONECT   38   37   40   41
CONECT   39   37   42   43
CONECT   40   38   44   45
CONECT   41   38
CONECT   42   39   44   46
CONECT   43   39
CONECT   44   40   42   47
CONECT   45   40
CONECT   46   42
CONECT   47   44
CONECT   48   32   15   52   53
CONECT   49   12    9   50   51
CONECT   50   49
CONECT   51   49
CONECT   52   48
CONECT   53   48
CONECT   54   32   55   56   57
CONECT   55   54
CONECT   56   54
CONECT   57   54
CONECT   58   15   59   60   61
CONECT   59   58
CONECT   60   58
CONECT   61   58
```

****

TITLE *trans*-Pt-MeR"S'"

```
REMARK   1 File created by GaussView 6.0.16
HETATM    1  C           0       1.182   3.272   0.365                    C
HETATM    2  C           0      -0.106   3.691  -0.038                    C
```

HETATM	3	C	0	0.395	5.030	-0.465	C
HETATM	4	C	0	1.795	4.604	-0.003	C
HETATM	5	O	0	-0.103	6.032	-0.980	O
HETATM	6	O	0	2.926	5.088	0.058	O
HETATM	7	N	0	1.805	2.193	0.875	N
HETATM	8	H	0	1.318	1.339	1.142	H
HETATM	9	C	0	3.245	2.224	1.203	C
HETATM	10	H	0	3.430	1.385	1.882	H
HETATM	11	H	0	3.480	3.151	1.733	H
HETATM	12	C	0	3.902	0.894	-0.894	C
HETATM	13	H	0	4.564	0.855	-1.762	H
HETATM	14	H	0	2.866	0.828	-1.233	H
HETATM	15	S	0	4.252	-0.692	0.099	S
HETATM	16	C	0	-2.436	3.999	-0.891	C
HETATM	17	C	0	-3.640	3.262	-0.868	C
HETATM	18	H	0	-2.251	4.958	-1.349	H
HETATM	19	C	0	-3.410	2.086	-0.140	C
HETATM	20	H	0	-4.584	3.555	-1.299	H
HETATM	21	C	0	-1.466	3.265	-0.183	C
HETATM	22	N	0	-2.085	2.107	0.290	N
HETATM	23	C	0	-1.475	1.027	1.059	C
HETATM	24	H	0	-0.601	1.412	1.590	H
HETATM	25	H	0	-2.177	0.715	1.840	H
HETATM	26	C	0	-1.108	-0.178	0.162	C
HETATM	27	H	0	-1.810	-0.208	-0.680	H
HETATM	28	H	0	-0.103	-0.029	-0.257	H
HETATM	29	C	0	-1.178	-1.489	0.954	C
HETATM	30	H	0	-0.809	-1.371	1.974	H
HETATM	31	H	0	-2.194	-1.895	0.974	H
HETATM	32	S	0	-0.090	-2.849	0.243	S
HETATM	33	C	0	-4.307	0.983	0.166	C
HETATM	34	H	0	-4.008	0.304	0.963	H
HETATM	35	C	0	-5.474	0.750	-0.482	C
HETATM	36	H	0	-5.741	1.393	-1.320	H
HETATM	37	C	0	-6.423	-0.334	-0.177	C
HETATM	38	C	0	-7.440	-0.632	-1.108	C
HETATM	39	C	0	-6.360	-1.093	1.013	C
HETATM	40	C	0	-8.352	-1.670	-0.873	C
HETATM	41	H	0	-7.508	-0.049	-2.022	H
HETATM	42	C	0	-7.268	-2.131	1.248	C

```
HETATM   43  H          0      -5.618  -0.857   1.770                   H
HETATM   44  C          0      -8.267  -2.427   0.305                   C
HETATM   45  H          0      -9.125  -1.885  -1.603                   H
HETATM   46  H          0      -7.209  -2.700   2.170                   H
HETATM   47  H          0      -8.974  -3.229   0.492                   H
HETATM   48  C          0       4.152   2.131  -0.035                   C
HETATM   49  H          0       5.194   2.150   0.305                   H
HETATM   50  H          0       4.004   3.021  -0.656                   H
HETATM   51 Cl          0       1.692  -0.736   2.278                  Cl
HETATM   52 Pt          0       2.090  -1.781   0.112                  Pt
HETATM   53 Cl          0       2.439  -2.702  -2.080                  Cl
HETATM   54  C          0       5.363  -1.613  -1.094                   C
HETATM   55  H          0       5.491  -2.619  -0.696                   H
HETATM   56  H          0       4.886  -1.667  -2.071                   H
HETATM   57  H          0       6.315  -1.083  -1.122                   H
HETATM   58  C          0      -0.764  -2.931  -1.502                   C
HETATM   59  H          0      -1.828  -3.162  -1.431                   H
HETATM   60  H          0      -0.589  -1.986  -2.016                   H
HETATM   61  H          0      -0.218  -3.722  -2.011                   H
END
CONECT    1    2    4    7
CONECT    2    1    3   21
CONECT    3    2    4    5
CONECT    4    1    3    6
CONECT    5    3
CONECT    6    4
CONECT    7    1    8    9
CONECT    8    7
CONECT    9    7   10   11   48
CONECT   10    9
CONECT   11    9
CONECT   12   13   14   48
CONECT   13   12
CONECT   14   12
CONECT   15   52   54
CONECT   16   17   18   21
CONECT   17   16   19   20
CONECT   18   16
CONECT   19   17   22   33
CONECT   20   17
```

```
CONECT   21   16    2   22
CONECT   22   19   21   23
CONECT   23   22   24   25   26
CONECT   24   23
CONECT   25   23
CONECT   26   23   27   28   29
CONECT   27   26
CONECT   28   26
CONECT   29   26   30   31   32
CONECT   30   29
CONECT   31   29
CONECT   32   29   52   58
CONECT   33   19   34   35
CONECT   34   33
CONECT   35   33   36   37
CONECT   36   35
CONECT   37   35   38   39
CONECT   38   37   40   41
CONECT   39   37   42   43
CONECT   40   38   44   45
CONECT   41   38
CONECT   42   39   44   46
CONECT   43   39
CONECT   44   40   42   47
CONECT   45   40
CONECT   46   42
CONECT   47   44
CONECT   48   12    9   49   50
CONECT   49   48
CONECT   50   48
CONECT   52   32   15   53
CONECT   53   52
CONECT   54   15   55   56   57
CONECT   55   54
CONECT   56   54
CONECT   57   54
CONECT   58   32   59   60   61
CONECT   59   58
CONECT   60   58
CONECT   61   58
```

****

TITLE *trans*-Pt-MeR"R'"

REMARK 1 File created by GaussView 6.0.16

```
HETATM    1  C     0    -1.267   3.197  -0.330    C
HETATM    2  C     0     0.015   3.659   0.040    C
HETATM    3  C     0    -0.515   4.995   0.444    C
HETATM    4  C     0    -1.909   4.521   0.013    C
HETATM    5  O     0    -0.035   6.019   0.931    O
HETATM    6  O     0    -3.054   4.973  -0.045    O
HETATM    7  N     0    -1.872   2.088  -0.799    N
HETATM    8  H     0    -1.371   1.233  -1.032    H
HETATM    9  C     0    -3.312   2.081  -1.116    C
HETATM   10  H     0    -3.473   1.237  -1.788    H
HETATM   11  H     0    -3.572   2.998  -1.653    H
HETATM   12  C     0    -3.901   0.833   1.075    C
HETATM   13  H     0    -4.493   0.900   1.989    H
HETATM   14  H     0    -2.841   0.811   1.336    H
HETATM   15  S     0    -4.265  -0.933   0.436    S
HETATM   16  C     0     2.349   4.026   0.859    C
HETATM   17  C     0     3.569   3.318   0.817    C
HETATM   18  H     0     2.149   4.981   1.320    H
HETATM   19  C     0     3.355   2.136   0.093    C
HETATM   20  H     0     4.514   3.633   1.233    H
HETATM   21  C     0     1.386   3.269   0.168    C
HETATM   22  N     0     2.024   2.125  -0.316    N
HETATM   23  C     0     1.427   1.037  -1.083    C
HETATM   24  H     0     0.535   1.408  -1.597    H
HETATM   25  H     0     2.123   0.747  -1.877    H
HETATM   26  C     0     1.102  -0.180  -0.190    C
HETATM   27  H     0     1.839  -0.221   0.621    H
HETATM   28  H     0     0.116  -0.045   0.276    H
HETATM   29  C     0     1.142  -1.477  -1.005    C
HETATM   30  H     0     0.703  -1.352  -1.996    H
HETATM   31  H     0     2.162  -1.863  -1.101    H
HETATM   32  S     0     0.130  -2.866  -0.242    S
HETATM   33  C     0     4.274   1.056  -0.230    C
HETATM   34  H     0     3.979   0.369  -1.022    H
HETATM   35  C     0     5.458   0.853   0.397    C
HETATM   36  H     0     5.723   1.502   1.231    H
```

```
HETATM  37 C      0     6.429  -0.207   0.077      C
HETATM  38 C      0     7.469  -0.479   0.991      C
HETATM  39 C      0     6.365  -0.968  -1.112      C
HETATM  40 C      0     8.403  -1.492   0.741      C
HETATM  41 H      0     7.537   0.106   1.905      H
HETATM  42 C      0     7.296  -1.982  -1.362      C
HETATM  43 H      0     5.605  -0.751  -1.856      H
HETATM  44 C      0     8.318  -2.252  -0.435      C
HETATM  45 H      0     9.193  -1.687   1.458      H
HETATM  46 H      0     7.237  -2.553  -2.283      H
HETATM  47 H      0     9.042  -3.036  -0.634      H
HETATM  48 C      0    -4.210   1.990   0.131      C
HETATM  49 H      0    -5.259   1.978  -0.188      H
HETATM  50 H      0    -4.091   2.914   0.709      H
HETATM  51 Cl     0    -1.847  -0.875  -2.180      Cl
HETATM  52 Pt     0    -2.056  -1.845   0.056      Pt
HETATM  53 Cl     0    -2.190  -2.659   2.306      Cl
HETATM  54 C      0    -5.141  -0.754  -1.211      C
HETATM  55 H      0    -4.418  -0.682  -2.021      H
HETATM  56 H      0    -5.742  -1.657  -1.315      H
HETATM  57 H      0    -5.794   0.118  -1.172      H
HETATM  58 C      0     0.929  -2.968   1.448      C
HETATM  59 H      0     1.990  -3.171   1.294      H
HETATM  60 H      0     0.766  -2.042   1.996      H
HETATM  61 H      0     0.441  -3.785   1.975      H
END
CONECT   1   2   4   7
CONECT   2   1   3  21
CONECT   3   2   4   5
CONECT   4   1   3   6
CONECT   5   3
CONECT   6   4
CONECT   7   1   8   9
CONECT   8   7
CONECT   9   7  10  11  48
CONECT  10   9
CONECT  11   9
CONECT  12  13  14  48
CONECT  13  12
CONECT  14  12
```

```
CONECT   15   52   54
CONECT   16   17   18   21
CONECT   17   16   19   20
CONECT   18   16
CONECT   19   17   22   33
CONECT   20   17
CONECT   21    2   16   22
CONECT   22   21   19   23
CONECT   23   22   24   25   26
CONECT   24   23
CONECT   25   23
CONECT   26   23   27   28   29
CONECT   27   26
CONECT   28   26
CONECT   29   26   30   31   32
CONECT   30   29
CONECT   31   29
CONECT   32   29   52   58
CONECT   33   19   34   35
CONECT   34   33
CONECT   35   33   36   37
CONECT   36   35
CONECT   37   35   38   39
CONECT   38   37   40   41
CONECT   39   37   42   43
CONECT   40   38   44   45
CONECT   41   38
CONECT   42   39   44   46
CONECT   43   39
CONECT   44   42   40   47
CONECT   45   40
CONECT   46   42
CONECT   47   44
CONECT   48    9   12   49   50
CONECT   49   48
CONECT   50   48
CONECT   52   15   32   53
CONECT   53   52
CONECT   54   15   55   56   57
CONECT   55   54
```

CONECT 56 54
CONECT 57 54
CONECT 58 32 59 60 61
CONECT 59 58
CONECT 60 58
CONECT 61 58

****

TITLE *trans*-Pt-MeS"S'"
REMARK 1 File created by GaussView 6.0.16
HETATM 1 C 0 0.947 3.263 0.475 C
HETATM 2 C 0 -0.344 3.636 0.038 C
HETATM 3 C 0 0.088 5.037 -0.242 C
HETATM 4 C 0 1.488 4.658 0.256 C
HETATM 5 O 0 -0.449 6.049 -0.693 O
HETATM 6 O 0 2.579 5.206 0.420 O
HETATM 7 N 0 1.618 2.185 0.927 N
HETATM 8 H 0 1.174 1.288 1.113 H
HETATM 9 C 0 3.040 2.285 1.315 C
HETATM 10 H 0 3.265 1.404 1.923 H
HETATM 11 H 0 3.189 3.174 1.934 H
HETATM 12 C 0 3.874 1.199 -0.861 C
HETATM 13 H 0 4.568 1.291 -1.700 H
HETATM 14 H 0 2.860 1.078 -1.247 H
HETATM 15 S 0 4.330 -0.431 0.010 S
HETATM 16 C 0 -2.652 3.891 -0.886 C
HETATM 17 C 0 -3.813 3.094 -0.974 C
HETATM 18 H 0 -2.501 4.891 -1.264 H
HETATM 19 C 0 -3.544 1.872 -0.341 C
HETATM 20 H 0 -4.739 3.354 -1.461 H
HETATM 21 C 0 -1.666 3.148 -0.210 C
HETATM 22 N 0 -2.233 1.917 0.127 N
HETATM 23 C 0 -1.619 0.837 0.892 C
HETATM 24 H 0 -0.825 1.249 1.523 H
HETATM 25 H 0 -2.372 0.438 1.581 H
HETATM 26 C 0 -1.082 -0.291 -0.017 C
HETATM 27 H 0 -1.725 -0.383 -0.899 H
HETATM 28 H 0 -0.081 -0.027 -0.383 H
HETATM 29 C 0 -1.048 -1.610 0.762 C
HETATM 30 H 0 -0.674 -1.465 1.777 H

```
HETATM   31  H         0     -2.031  -2.089   0.785                  H
HETATM   32  S         0      0.122  -2.837  -0.060                  S
HETATM   33  C         0     -4.393   0.705  -0.157                  C
HETATM   34  H         0     -3.905  -0.230   0.115                  H
HETATM   35  C         0     -5.739   0.716  -0.310                  C
HETATM   36  H         0     -6.231   1.667  -0.515                  H
HETATM   37  C         0     -6.626  -0.453  -0.196                  C
HETATM   38  C         0     -8.015  -0.246  -0.057                  C
HETATM   39  C         0     -6.146  -1.781  -0.225                  C
HETATM   40  C         0     -8.896  -1.328   0.073                  C
HETATM   41  H         0     -8.400   0.770  -0.044                  H
HETATM   42  C         0     -7.024  -2.862  -0.094                  C
HETATM   43  H         0     -5.088  -1.971  -0.380                  H
HETATM   44  C         0     -8.403  -2.641   0.060                  C
HETATM   45  H         0     -9.960  -1.147   0.183                  H
HETATM   46  H         0     -6.640  -3.877  -0.125                  H
HETATM   47  H         0     -9.083  -3.481   0.157                  H
HETATM   48  C         0      3.992   2.372   0.109                  C
HETATM   49  H         0      5.017   2.445   0.491                  H
HETATM   50  H         0      3.792   3.299  -0.440                  H
HETATM   51 Cl         0      1.776  -0.843   2.213                  Cl
HETATM   52 Pt         0      2.234  -1.643  -0.046                  Pt
HETATM   53 Cl         0      2.607  -2.379  -2.293                  Cl
HETATM   54  C         0      5.472  -1.194  -1.263                  C
HETATM   55  H         0      5.657  -2.221  -0.949                  H
HETATM   56  H         0      4.985  -1.195  -2.236                  H
HETATM   57  H         0      6.394  -0.613  -1.256                  H
HETATM   58  C         0      0.346  -4.038   1.361                  C
HETATM   59  H         0     -0.611  -4.525   1.554                  H
HETATM   60  H         0      1.087  -4.770   1.043                  H
HETATM   61  H         0      0.701  -3.485   2.233                  H
END
CONECT    1    2    4    7
CONECT    2    1    3   21
CONECT    3    2    4    5
CONECT    4    1    3    6
CONECT    5    3
CONECT    6    4
CONECT    7    1    8    9
CONECT    8    7
```

```
CONECT    9    7   10   11   48
CONECT   10    9
CONECT   11    9
CONECT   12   13   14   48
CONECT   13   12
CONECT   14   12
CONECT   15   52   54
CONECT   16   17   18   21
CONECT   17   16   19   20
CONECT   18   16
CONECT   19   17   22   33
CONECT   20   17
CONECT   21   16    2   22
CONECT   22   19   21   23
CONECT   23   22   24   25   26
CONECT   24   23
CONECT   25   23
CONECT   26   23   27   28   29
CONECT   27   26
CONECT   28   26
CONECT   29   26   30   31   32
CONECT   30   29
CONECT   31   29
CONECT   32   29   52   58
CONECT   33   19   34   35
CONECT   34   33
CONECT   35   33   36   37
CONECT   36   35
CONECT   37   35   38   39
CONECT   38   37   40   41
CONECT   39   37   42   43
CONECT   40   38   44   45
CONECT   41   38
CONECT   42   39   44   46
CONECT   43   39
CONECT   44   42   40   47
CONECT   45   40
CONECT   46   42
CONECT   47   44
CONECT   48   12    9   49   50
```

```
CONECT   49   48
CONECT   50   48
CONECT   52   32   15   53
CONECT   53   52
CONECT   54   15   55   56   57
CONECT   55   54
CONECT   56   54
CONECT   57   54
CONECT   58   32   59   60   61
CONECT   59   58
CONECT   60   58
CONECT   61   58
```

****

TITLE *trans*-Pt-MeS"R"'

```
REMARK   1 File created by GaussView 6.0.16
HETATM    1  C     0   -0.686   2.947  -0.579     C
HETATM    2  C     0    0.600   3.425  -0.272     C
HETATM    3  C     0    0.197   4.847  -0.479     C
HETATM    4  C     0   -1.187   4.329  -0.929     C
HETATM    5  O     0    0.728   5.944  -0.317     O
HETATM    6  O     0   -2.231   4.785  -1.395     O
HETATM    7  N     0   -1.347   1.767  -0.579     N
HETATM    8  H     0   -1.013   0.992  -0.007     H
HETATM    9  C     0   -2.712   1.670  -1.131     C
HETATM   10  H     0   -2.897   0.618  -1.376     H
HETATM   11  H     0   -2.750   2.239  -2.064     H
HETATM   12  C     0   -3.879   1.555   1.183     C
HETATM   13  H     0   -4.630   2.041   1.811     H
HETATM   14  H     0   -2.921   1.556   1.709     H
HETATM   15  S     0   -4.376  -0.275   1.176     S
HETATM   16  C     0    2.861   3.683   0.823     C
HETATM   17  C     0    4.012   2.872   0.940     C
HETATM   18  H     0    2.713   4.683   1.200     H
HETATM   19  C     0    3.759   1.662   0.276     C
HETATM   20  H     0    4.927   3.126   1.453     H
HETATM   21  C     0    1.895   2.953   0.116     C
HETATM   22  N     0    2.461   1.720  -0.221     N
HETATM   23  C     0    1.844   0.722  -1.093     C
HETATM   24  H     0    1.029   1.211  -1.638     H
```

HETATM 25 H 0 2.577 0.396 -1.840 H
HETATM 26 C 0 1.296 -0.480 -0.304 C
HETATM 27 H 0 2.114 -1.031 0.175 H
HETATM 28 H 0 0.666 -0.119 0.515 H
HETATM 29 C 0 0.513 -1.410 -1.233 C
HETATM 30 H 0 -0.228 -0.873 -1.834 H
HETATM 31 H 0 1.175 -1.974 -1.897 H
HETATM 32 S 0 -0.428 -2.698 -0.221 S
HETATM 33 C 0 4.623 0.514 0.053 C
HETATM 34 H 0 4.169 -0.369 -0.393 H
HETATM 35 C 0 5.943 0.489 0.355 C
HETATM 36 H 0 6.402 1.393 0.754 H
HETATM 37 C 0 6.850 -0.657 0.179 C
HETATM 38 C 0 8.240 -0.448 0.304 C
HETATM 39 C 0 6.392 -1.963 -0.105 C
HETATM 40 C 0 9.147 -1.502 0.132 C
HETATM 41 H 0 8.607 0.549 0.530 H
HETATM 42 C 0 7.296 -3.016 -0.277 C
HETATM 43 H 0 5.327 -2.163 -0.169 H
HETATM 44 C 0 8.679 -2.791 -0.163 C
HETATM 45 H 0 10.212 -1.319 0.229 H
HETATM 46 H 0 6.927 -4.014 -0.490 H
HETATM 47 H 0 9.378 -3.610 -0.294 H
HETATM 48 C 0 -3.798 2.220 -0.189 C
HETATM 49 H 0 -4.764 2.171 -0.706 H
HETATM 50 H 0 -3.603 3.287 -0.023 H
HETATM 51 Cl 0 -3.639 -2.313 -1.552 Cl
HETATM 52 Pt 0 -2.445 -1.478 0.364 Pt
HETATM 53 Cl 0 -1.230 -0.470 2.201 Cl
HETATM 54 C 0 -5.726 -0.301 -0.115 C
HETATM 55 H 0 -5.330 -0.043 -1.096 H
HETATM 56 H 0 -6.104 -1.321 -0.144 H
HETATM 57 H 0 -6.500 0.396 0.210 H
HETATM 58 C 0 -0.844 -3.910 -1.585 C
HETATM 59 H 0 0.070 -4.455 -1.823 H
HETATM 60 H 0 -1.613 -4.578 -1.201 H
HETATM 61 H 0 -1.242 -3.373 -2.445 H
END
CONECT 1 2 4 7
CONECT 2 1 3 21

```
CONECT    3    2    4    5
CONECT    4    1    3    6
CONECT    5    3
CONECT    6    4
CONECT    7    1    8    9
CONECT    8    7
CONECT    9    7   10   11   48
CONECT   10    9
CONECT   11    9
CONECT   12   13   14   48
CONECT   13   12
CONECT   14   12
CONECT   15   52   54
CONECT   16   17   18   21
CONECT   17   16   19   20
CONECT   18   16
CONECT   19   17   22   33
CONECT   20   17
CONECT   21    2   16   22
CONECT   22   21   19   23
CONECT   23   22   24   25   26
CONECT   24   23
CONECT   25   23
CONECT   26   23   27   28   29
CONECT   27   26
CONECT   28   26
CONECT   29   26   30   31
CONECT   30   29
CONECT   31   29
CONECT   32   52   58
CONECT   33   19   34   35
CONECT   34   33
CONECT   35   33   36   37
CONECT   36   35
CONECT   37   35   38   39
CONECT   38   37   40   41
CONECT   39   37   42   43
CONECT   40   38   44   45
CONECT   41   38
CONECT   42   39   44   46
```

CONECT 43 39
CONECT 44 42 40 47
CONECT 45 40
CONECT 46 42
CONECT 47 44
CONECT 48 12 9 49 50
CONECT 49 48
CONECT 50 48
CONECT 51 52
CONECT 52 51 15 32
CONECT 54 15 55 56 57
CONECT 55 54
CONECT 56 54
CONECT 57 54
CONECT 58 32 59 60 61
CONECT 59 58
CONECT 60 58
CONECT 61 58

****

5. References

[ ] Arunkumar, E.; Fu, N.; Smith, B. D. Squaraine-Derived Rotaxanes: Highly Stable, Fluorescent near-IR Dyes. *Chem. - Eur. J.* **2006**, *12*, 4684–4690. https://doi.org/10.1002/chem.200501541.

[2] Demeter, A. First Steps in Photophysics. I. Fluorescence Yield and Radiative Rate Coefficient of 9,10-Bis(phenylethynyl)anthracene in Paraffins. *J. Phys. Chem. A* **2014**, *118*, 9985–9993. https://doi.org/10.1021/jp507626h.

[3] Bassotti, E.; Carbone, P.; Credi, A.; Di Stefano, M.; Masiero, S.; Negri, F.; Orlandi, G.; Spada, G. P. Effect of Strain on the Photoisomerization and Stability of a Congested Azobenzenophane: A Combined Experimental and Computational Study. *J. Phys. Chem. A* **2006**, *110*, 12385–12394. https://doi.org/10.1021/jp062428b.

[4] Higashiguchi, K.; Matsuda, K.; Asano, Y.; Murakami, A.; Nakamura, S.; Irie, M. Photochromism of Dithienylethenes Containing Fluorinated Thiophene Rings. *Eur. J. Org. Chem.* **2005**, *2005*, 91–97. https://doi.org/10.1002/ejoc.200400441.
